# Peripheral Dendritic Functionalization Effects on the Self-Assembly and Thermoresponsive Behavior of Nonionic Amphiphilic Linear–Dendritic Block Copolymers in Aqueous Media

**DOI:** 10.1021/acs.macromol.6c00544

**Published:** 2026-07-09

**Authors:** Javier Martín-Martín, Luis Folch-Cirujeda, Cristina Yus, Aurora Nogales, Tiberio A. Ezquerra, Miriam Abad, Víctor Sebastián, Luis Oriol, Milagros Piñol

**Affiliations:** † Instituto de Nanociencia y Materiales de Aragón (INMA), CSIC-Universidad de Zaragoza, Zaragoza 50009, Spain; ‡ Departamento de Química Orgánica, Facultad de Ciencias, Universidad de Zaragoza, Zaragoza 50009, Spain; § Department of Chemical Engineering and Environmental Technology, Universidad de Zaragoza, Zaragoza 50018, Spain; ∥ Instituto de Estructura de la Materia (IEM-CSIC), Madrid 28006, Spain; ⊥ Networking Research Center on Bioengineering, Biomaterials and Nanomedicine (CIBER-BBN), Madrid 28029, Spain; # Laboratorio de Microscopías Avanzadas, Universidad de Zaragoza, Zaragoza 50018, Spain

## Abstract

Nonionic amphiphilic block copolymers with a precise linear–dendritic architecture that exhibit thermoresponsive behavior in water are described. These copolymers consist of a hydrophilic poly­(ethylene glycol) (PEG) block connected to a thermoresponsive block based on the fourth generation of 2,2-bis­(hydroxymethyl)­propionic acid (bis-MPA) polyester dendron, functionalized at the periphery with either ureido or glycinamide groups. In water, these linear–dendritic block copolymers (LDBCs) self-assemble into different nanostructures whose size, morphology, and thermoresponse are strongly dependent on the peripheral groups and PEG chain length. The LDBC bearing peripheral ureido groups self-assembles into small micelles that, above the cloud point temperature (*T*_cp_), evolve into a coacervate phase rather than fully dissolve. In contrast, the glycinamide-functionalized LDBCs display characteristic UCST-type behavior. The results demonstrate that subtle structural variations in the dendron periphery within a precise linear–dendritic architecture induce significant differences in the self-assembly and thermoresponse in water. Preliminary studies on the encapsulation and thermally triggered release of Nile Red, together with high cytocompatibility of these LDBCs toward several eukaryotic cell lines, suggest their potential for drug delivery systems.

## Graphical abstract

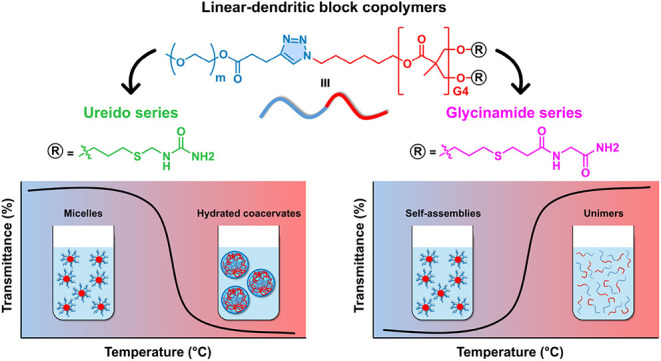

## Introduction

Temperature is one of the most commonly applied stimuli in smart polymers due to its ease of control and manipulation.^1^ In biomedical applications, the usefulness of thermoresponsive polymers largely depends on their ability to undergo substantial changes in water solubility with temperature, which arise primarily from changes in the balance between polymer–polymer and polymer–water interactions as a function of temperature.^2–4^ Depending on the temperature behavior, two types of polymers can be distinguished: those with a lower critical solution temperature (LCST) and those with an upper critical solution temperature (UCST). LCST polymers are soluble in water below a specific temperature known as the cloud point temperature (*T*_cp_), but become insoluble when this temperature is exceeded. UCST polymers show the opposite behavior, as they become soluble when the temperature rises above *T*_cp_.^5^

Amphiphilic block copolymers (BCs) with UCST thermoresponsiveness, specifically consisting of a hydrophilic block and a UCST-thermoresponsive block, spontaneously self-assemble in water into stable nanostructures below their *T*_cp_.^6^ This property makes them potentially attractive for drug delivery applications, as they are expected to encapsulate therapeutic molecules below *T*_cp_ and release them in a controlled manner upon heating above *T*_cp_.^7^ Several hydrogen bond-forming groups have been incorporated into amphiphilic BCs, mostly with a linear–linear architecture, to induce UCST behavior, notably amides,^8^ ureido,^9^ and glycinamides.^10^ Deng et al. prepared poly­(ethylene glycol)-*b*-poly­(*N*-acryloylglycinamide-*co*-acrylonitrile) (PEG-*b*-P­(NAGA-*co*-AN)) BCs with a *T*_cp_ slightly higher than physiological temperature. In water, these amphiphilic BCs formed micelles that, when loaded with doxorubicin (DOX) and IR780, underwent NIR-triggered disassembly with complete drug release, yielding synergistic chemo-photothermal effects.^10^ Fujihara et al. reported ureido-containing amphiphilic linear–linear BCs that self-assembled into micelles capable of encapsulating *N*-phenyl-1-naphthylamine below *T*_cp_ and disassembled above physiological temperature.^9^ Recently, we reported amphiphilic linear–linear BCs composed of a hydrophilic PEG block and a hydrophobic degradable polycarbonate block bearing UCST-promoting ureido pendant groups, which exhibited a complex thermoresponsive behavior. Below the *T*_cp_, the BCs self-assembled into nanostructures, which transformed into hydrated physical nanogel-like condensates upon being heated above the *T*_cp_.^11^

Compared with linear polymers, dendrimers and dendrons have a perfectly defined hyperbranched architecture, providing highly precise control over structural elements, including the number of peripheral functional groups, which can serve as attachment points for functional moieties, thereby allowing fine-tuned modulation of their properties. However, despite these advantages, the development of dendritic polymers exhibiting UCST behavior has remained largely unexplored. Zhou et al. synthesized enzymatically degradable star polypeptides with poly­(l-ornithine-*co*-l-citrulline) chains grown from the periphery of the fourth-generation poly­(amido amine) (PAMAM) dendrimer for functional surface coatings.^12^ Tamaki and Kojima published several works on ionic PAMAM dendrimers with pH-dependent UCST behavior, which were used to encapsulate Rose Bengal below the *T*_cp_.^13–15^

Among dendritic architectures, linear–dendritic BCs (LDBCs), which integrate the distinct properties of both macromolecular structures, are a notable platform for designing responsive nanostructures with precise control over functionality and self-assembly behavior. In this context, we reported a series of amphiphilic LDBCs comprising a PEG block and the fourth generation of a 2,2-bis­(hydroxymethyl)­propionic acid (bis-MPA) dendron functionalized at the periphery with 4-isobutyloxyazobenzene moieties, which self-assembled in aqueous solution to form light-responsive vesicles capable of encapsulating both hydrophobic and hydrophilic fluorescent model compounds. Upon UV irradiation, these vesicles undergo structural disruption, triggering the release of their encapsulated cargo.^16,17^

Here, we report the preparation and thermoresponsive behavior of polymer self-assemblies in water from amphiphilic LDBCs composed of a hydrophilic block and a nonionic thermoresponsive dendron with peripheral UCST-promoting ureido (**U**) or glycinamide (**GA**) groups. The linear segment is a hydrophilic PEG block. The dendritic segment is the fourth generation of a bis-MPA polyester dendron with 16 peripheral groups, featuring their nonimmunogenic, nontoxic, and potentially biodegradable properties.^18^ In addition, the ureido- and glycinamide-functionalized dendrons are reported for comparison with the LDBCs. As a proof of concept, Nile Red, a model hydrophobic probe, was used to evaluate the possible potential of these LDBC self-assemblies as temperature-triggered delivery systems. Additionally, the cytocompatibility of LDBCs was assessed through cytotoxicity studies on several eukaryotic cell lines.

## Results and Discussion

### Synthesis and Characterization of Dendritic Structures

Building on previous work, the target dendrons (**C**_**6**_ **-d16U** and **C**_**6**_ **-d16GA**) and LDBCs (**PEG** *_**m**_* **-** ***b*** **-d16U** and **PEG** *_**m**_* **-** ***b*** **-d16GA**, where **m** denotes the DP of PEG) were synthesized using two orthogonal click reactions, namely, Cu­(I) azide–alkyne cycloaddition (CuAAC) and photoinduced thiol–ene reaction, starting from the fourth generation bis-MPA dendron bearing peripheral allyl groups and an azido group at the focal point (**N**_**3**_ **-d16Allyl**) (Scheme 1).^19,20^ The chosen sequence of click reactions is critical. The CuAAC reaction was performed first, followed by the thiol–ene reaction, to avoid azide degradation under UV irradiation and to prevent coordination of Cu­(II) ions with ureido or glycinamide groups, which would hinder purification and significantly reduce the reaction efficiency. One ureido-functionalized polymer (**PEG**_**45**_ **-** ***b*** **-d16U**) was obtained, whereas three glycinamide-functionalized LDBCs (**PEG** *_**m**_* **-** ***b*** **-d16GA**) were prepared with different PEG lengths (*m* = 45, 113, and 226). Therefore, **N**_**3**_ **-d16Allyl** was coupled to either hex-1-yne or to alkyne-terminated PEG, **PEG** *_**m**_* **-Alkyne** (*m* = 45, 113, and 226), to give the **C**_**6**_ **-d16Allyl** dendron or the **PEG** *_**m**_* **-** ***b*** **-d16Allyl** LDBCs, respectively. Then, the peripheral allyl groups were functionalized with ureido or glycinamide groups through a light-induced thiol–ene reaction to yield the target model **C**_**6**_ **-d16U** and **C**_**6**_ **-d16GA** dendrons and **PEG**_**45**_ **-** ***b*** **-d16U** and **PEG** *_**m**_* **-** ***b*** **-d16GA** (with *m* = 45, 113, and 226) LDBCs, respectively. Full experimental details are given in the Supporting Information section (Table S1 and Figures S1–S19).

**Scheme 1:**
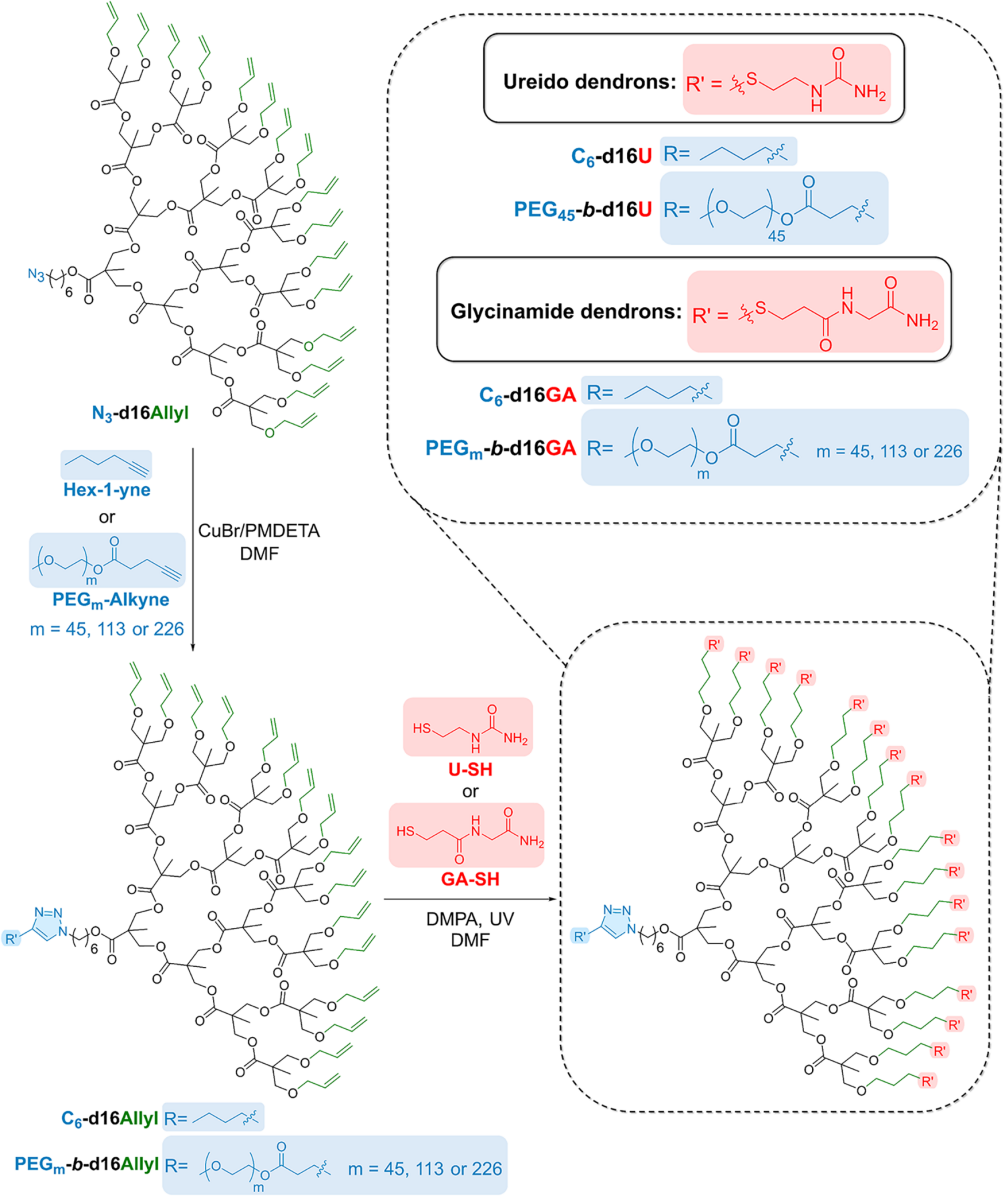
Synthetic Strategy for the Preparation of Model **C**_**6**_ **-d16U** and **C**_**6**_ **-d16GA** Dendrons and **PEG**_**45**_ **-** ***b*** **-d16U** and **PEG** *_**m**_* **-** ***b*** **-d16GA** LDBCs

**C**_**6**_ **-d16Allyl** and **PEG** *_**m**_* **-** ***b*** **-d16Allyl** were obtained by CuAAC in deoxygenated DMF, using CuBr and *N*,*N*,*N*′,*N*″,*N*″-pentamethyldiethylenetriamine (PMDETA) as a catalytic system. For **C**_**6**_ **-d16Allyl**, a slight excess of hex-1-yne was employed (approximately alkyne/azide = 1.2:1 molar ratio) that, upon completion of the reaction, was removed by simple evaporation. In contrast, a slight excess of **N**_**3**_ **-d16Allyl** was used for the synthesis of **PEG** *_**m**_* **-** ***b*** **-d16Allyl** (approximately alkyne/azide was 1:1.1 molar ratio) to facilitate the purification by precipitating **PEG** *_**m**_* **-** ***b*** **-d16Allyl** in cold hexane, where **N**_**3**_ **-d16Allyl** remains soluble. The chemical structure of the LDBCs, ensuring the absence of residual blocks, was verified by ^1^H NMR and FTIR spectroscopies, SEC, and MALDI-TOF MS (Figures 1, S3–S5, S12, and S14–S19).

**Figure 1:**
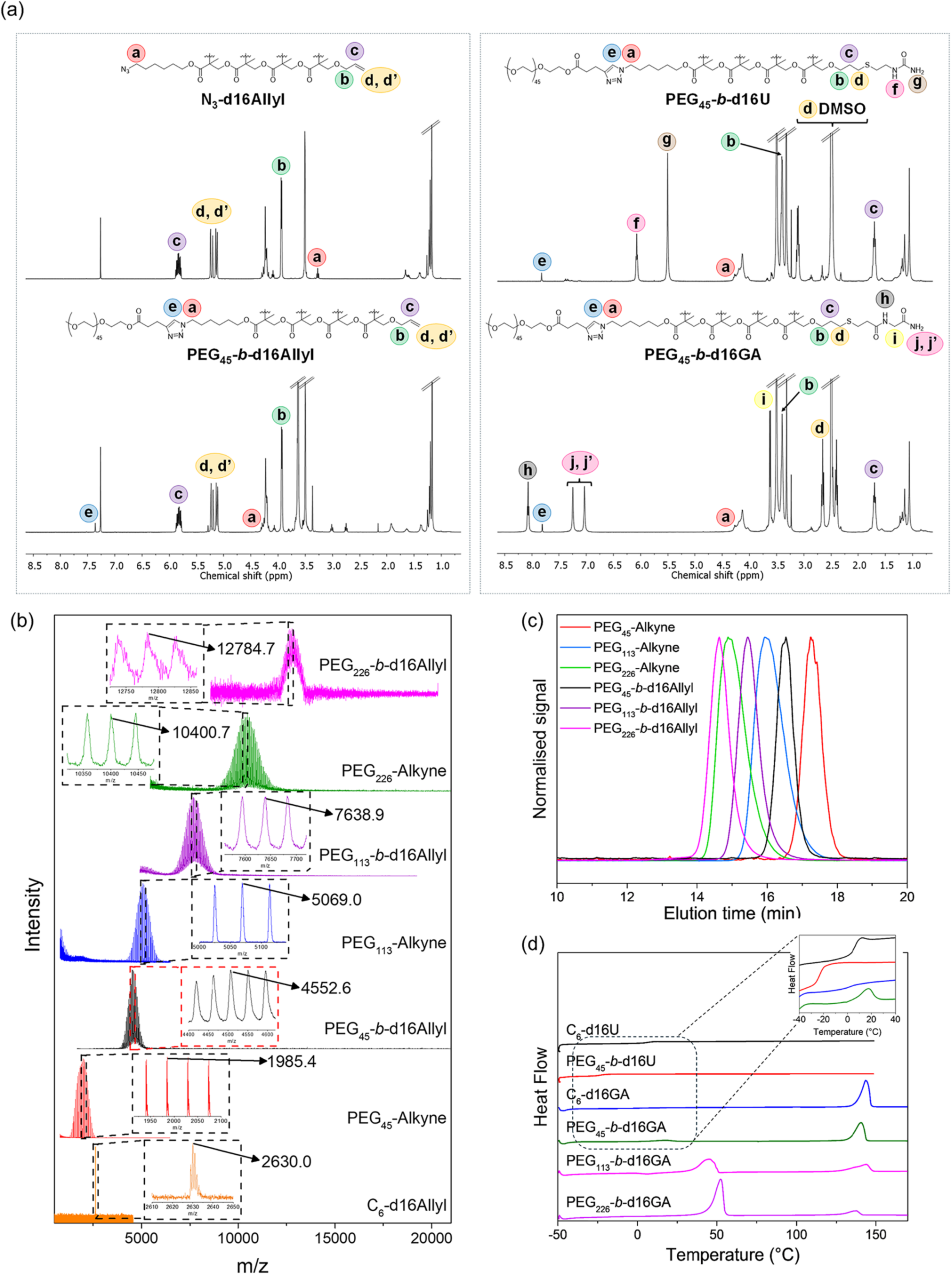
(a) ^1^H NMR (400 MHz, CDCl_3_) spectra of **N**_**3**_-d16Allyl and **PEG**_**45**_-***b***-**d16Allyl** and ^1^H NMR (400 MHz, DMSO-d_6_) spectra of **PEG**_**45**_-***b***-**d16U** and **PEG**_**45**_-***b*** **-d16GA**. (b) MALDI-TOF mass spectra with expanded insets, and (c) SEC traces of **PEG** *_**m**_*-Alkyne and **PEG** *_**m**_*-***b*** **-d16Allyl**. (d) DSC curves corresponding to the second heating scan of model dendrons and LDBCs registered at a 10 °C min^–1^ rate (exo down).

The periphery of the dendrons was functionalized with ureido or glycinamide units via thiol–ene reaction under UV light, using 2,2-dimethoxy-2-phenylacetophenone (DMPA) as the radical initiator. Complete functionalization of the allyl groups was verified by ^1^H NMR through the disappearance of the alkene signals (labeled as *c*, *d*, and *d*′ for **PEG**_**45**_ **-** ***b*** **-d16Allyl**) and the appearance of new resonances corresponding to the ureido (labeled as *f* and *g*) and glycinamide moieties (labeled as *h*, *i*, and *j*, *j*′) (Figure 1a). Also, FTIR spectroscopy revealed new bands in the 3400–3100 cm^–1^ region, corresponding to peripheral ureido and glycinamide units (Figure S12).

The thermal characterization of LDBCs was performed by thermogravimetric analysis (TGA) and differential scanning calorimetry (DSC) (Table 1), where thermal data of the commercial **PEG** *_**m**_* **–OH** precursors are also included as reference. TGA performed under a nitrogen atmosphere showed that glycinamide-functionalized LDBCs exhibited higher thermal stability, with the onset of mass loss above 290 °C, than the ureido-functionalized LDBC, which presented an initial mass loss above 190 °C (Figure S20).

**Table 1:** Thermal Properties of Commercial PEG Precursors, Model Dendrons, and LDBCs

**polymer**	**weight ratio PEG/dendron^a^**	**TGA (°C)^b^**	***T***_**g**_ **(°C)^c^**	***T***_**m**_ **(°C) [Δ*H***_**m**_ **(J g** ^**–1**^ **)]** ^d^
**PEG**_**45**_ **–OH**		382		48 [161.0]
**PEG**_**113**_ **–OH**		376		54 [159.0]
**PEG**_**226**_ **–OH**		372		62 [180.0]
**C**_**6**_ **-d16U**		194	6	
**PEG**_**45**_ **-** ***b*** **-d16U**	32:68	186	–23	
**C**_**6**_ **-d16GA**		297	2	144 [55.6]
**PEG**_**45**_ **-** ***b*** **-d16GA**	28:72	301		18 [5.2], 141 [41.0]
**PEG**_**113**_ **-** ***b*** **-d16GA^e^**	49:51	292		46 [48.8], 144 [28.2]
**PEG**_**226**_ **-** ***b*** **-d16GA**	66:34	289		52 [77.4], 138 [12.6]

a Weight ratio of the PEG and dendritic blocks in LDBCs.

b Decomposition temperature associated with mass loss determined by TGA as the onset point in the weight loss curve recorded at 10 °C min^–1^.

c Glass transition temperature (*T*_g_) determined at the half height of baseline jump by DSC during the second heating scan at 10 °C min^–1^.

d Melting temperature (*T*_m_) calculated at the maximum of the corresponding peak and associated enthalpy (Δ*H*_m_) determined by DSC during the second heating scan at 10 °C min^–1^.

e Cold crystallization at *T*_c_ = 7 °C with associated Δ*H*_c_ = −2 J g^–1^.

Thermal transitions were assessed by DSC (Figures 1d and S21). The ureido-functionalized model dendron, **C**_**6**_ **-d16U**, was a semicrystalline material, exhibiting a glass transition temperature (*T*_g_) at 6 °C and a melting temperature (*T*_m_) at 61 °C (Δ*H*_m_ = 29.5 J g^–1^) during the heating of the pristine sample (Figure S21a). The melted dendron did not crystallize on cooling (Figure S21b), and only a glass transition was observed at 6 °C during the second heating scan (Figure 1d). The **PEG**_**45**_ **-** ***b*** **-d16U** LDBC showed a glass transition at −22 °C and a minor crystalline fraction that melted at 47 °C (Δ*H*_m_ = 0.4 J g^–1^) during the first heating (Figure S21a). The lower *T*_g_ compared to that of **C**_**6**_ **-d16U** can be related to the partial miscibility between the PEG and dendritic blocks in the bulk. On subsequent heating scans, only a reproducible *T*_g_ was registered at −23 °C (Figure 1d).^21,22^ Conversely, the **C**_**6**_ **-d16GA** dendron was a semicrystalline solid that displayed an endothermic transition at approximately 140 °C in every heating scan (Table 1, Figure 1d). For **PEG** *_**m**_* **-** ***b*** **-d16GA**, crystallization from the molten state depended on the PEG-to-dendron ratio. Upon cooling, crystallization of the PEG block was strongly hindered in **PEG**_**45**_ **-** ***b*** **-d16GA** (the shortest PEG), while crystallization of the PEG was observed for **PEG**_**113**_ **-** ***b*** **-d16GA** and **PEG**_**226**_ **-** ***b*** **-d16GA** close to their precursor. In contrast, dendron crystallization was evident in **PEG**_**45**_ **-** ***b*** **-d16GA** but barely observed in **PEG**_**113**_ **-** ***b*** **-d16GA** and **PEG**_**226**_ **-** ***b*** **-d16GA** (Figure S21b). Upon subsequent heating, two endothermic transitions were observed, corresponding to the melting of the PEG and dendritic blocks. Dendron melting occurred around 140 °C, with Δ*H*_m_ decreasing as its weight fraction decreased, while PEG melted at lower temperatures (Figure 1d). Overall, it can be inferred that the glycinamide groups appear to favor the crystallization of the dendron by providing an additional hydrogen bond acceptor within the functional unit, which strengthens supramolecular interactions.

### Self-Assembly Properties of LDBCs

Given their amphiphilic nature, it would be expected that LDBCs self-assemble in water above the critical aggregation concentration (CAC) with dendron blocks confined within hydrophobic domains and the PEG chains forming a highly swollen, solvated stabilizing corona. Therefore, self-assembly was explored by nanoprecipitation using the cosolvent technique in which water acts as a selective solvent and DMSO as a common solvent for both blocks. Once the organic solvent was removed, the resulting **PEG**_**45**_ **-** ***b-*** **d16U** aqueous dispersion was nearly transparent and stable. In contrast, the aqueous dispersion of **PEG**_**45**_ **-** ***b*** **-d16GA** was initially turbid and eventually precipitated in a few hours. To address this issue, the PEG chain length was increased (*m* = 113 and 226) to enhance the dispersibility of the glycinamide LDBC self-assemblies. Indeed, dispersibility of **PEG**_**113**_ **-** ***b*** **-d16GA** and **PEG**_**226**_ **-** ***b*** **-d16GA** improved substantially, yielding noticeably less cloudy dispersions. The CAC of the LDBCs was determined by fluorescence spectroscopy using Nile Red, with calculated values ranging from 35 to 55 μg mL^–1^ (Figure S22). These values are typical for this type of amphiphilic copolymers.^16^

The morphology and size of **PEG**_**45**_ **-** ***b*** **-d16U** self-assemblies were examined by transmission electron microscopy (TEM), dynamic light scattering (DLS), and small-angle X-ray scattering (SAXS) (Figures 2a,i, S23, and S24). TEM images of the **PEG**_**45**_ **-** ***b-*** **d16U** dispersion revealed the coexistence of spherical micelles, with an average diameter of 11 ± 3 nm, and cylindrical micelles with a similar cross-sectional diameter of 10 ± 3 nm that might result from the fusion of the spherical micelles (Figure 2a).^23,24^ The DLS measurements were broadly consistent with the predominantly spherical micelles population observed by TEM, showing a main size distribution with an average hydrodynamic diameter (*D*_h_) of 12 ± 4 nm (PDI = 0.44) at 25 °C (Figure S23a). In addition, the intensity size distribution revealed minor populations at larger apparent *D*_h_ values, most likely arising from cylindrical micelles observed by TEM and/or from the presence of larger micellar aggregates (Figure S23b).^21,25,26^ The SAXS profile (Figure 2i) showed a low-q plateau that fits well with a spherical model, yielding a diameter (2*R*) of approximately 12 ± 3 nm, as determined using the Guinier approximation (Figure S24).^27^ This is in good agreement with the TEM images and DLS curves.

**Figure 2:**
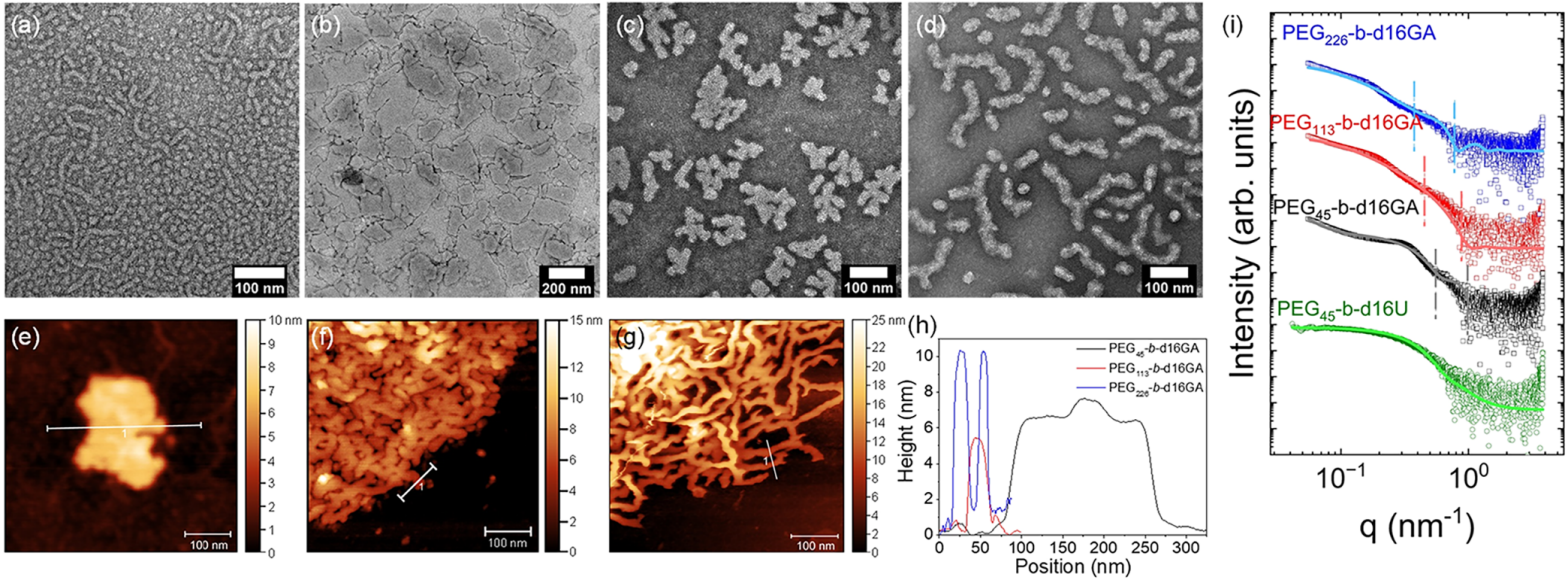
TEM images (samples stained with uranyl acetate) of self-assemblies formed by (a) **PEG**_**45**_-***b*** **-d16U**, (b) **PEG**_**45**_-***b*** **-d16GA**, (c) **PEG**_**113**_-***b*** **-d16GA**, and (d) **PEG**_**226**_-***b*** **-d16GA** at RT and a polymer concentration of 1.0 mg mL^–1^. AFM height images of self-assemblies formed by (e) **PEG**_**45**_-***b*** **-d16GA**, (f) **PEG**_**113**_-***b*** **-d16GA**, and (g) **PEG**_**226**_-***b*** **-d16GA** at RT and a polymer concentration of 1.0 mg mL^–1^. (h) Linear height profiles from panels (e–g). (i) SAXS intensity from all LDBC self-assemblies at 20 °C. Continuous lines correspond to the model that describes the scattering in each case.

**PEG** *_**m**_* **-** ***b*** **-d16GA** self-assemblies were studied by TEM, atomic force microscopy (AFM), and SAXS (Figures 2b–i and S24). By TEM, clear morphological differences were observed compared to those of **PEG**_**45**_ **-** ***b*** **-d16U**. **PEG**_**45**_ **-** ***b*** **-d16GA** exhibited platelet-like morphologies, whereas **PEG**_**113**_ **-** ***b*** **-d16GA** and **PEG**_**223**_ **-** ***b*** **-d16GA** exhibited elongated and branched-like morphologies, seemingly formed by nearly globular subunits (Figure 2b–d). Unlike **PEG**_**45**_ **-** ***b*** **-d16U**, SAXS profiles of all **PEG** *_**m**_* **-** ***b*** **-d16GA** self-assemblies exhibited a pronounced slope at low q, indicative of aggregation or the presence of large-scale structures. In the intermediate-*q* region, the profiles reveal multiple changes in slope, suggesting structural transitions or hierarchical organization. In addition, these intermediate regions were different, depending on the length of the PEG segment. At high *q*, within the range highlighted by the dashed vertical lines in Figure 2i, the three samples showed comparable scattering behavior, pointing to similar local structural motifs at short length scales. These features can correspond to the small globular subunits observed by the TEM. According to the SAXS results, the radius of gyration (*R*_g_) of these small entities is approximately 4 nm (Figure S24).

**PEG**_**113**_ **-** ***b*** **-d16GA** self-assembled into a dendritic morphology characterized by branched structures as observed by both TEM and AFM (Figure 2c,f,h). These nanostructures had an average cross-sectional width of 21 ± 8 nm as determined by TEM and an average height of 5.2 ± 0.8 nm as determined by AFM. The experimental SAXS data were fitted using the Mass Fractal model (eq S2).^28^ Further details of the fitting can be found in the Supporting Information. From the mass fractal analysis, a primary particle radius of 4.1 nm is obtained, in agreement with the *R*_g_ obtained from the Guinier approximation for both **PEG**_**113**_ **-** ***b*** **-d16GA** and **PEG**_**226**_ **-** ***b*** **-d16GA**.

**PEG**_**226**_ **-** ***b*** **-d16GA**, with the longest PEG chain, gave more elongated branched morphologies, with a cross-sectional width of ca. 26 ± 9 nm and a height of 8.9 ± 2.9 nm, as observed by TEM and AFM images, respectively (Figure 2d,g,h). The SAXS profile was well fitted employing the pearl-necklace model.^29^ This model describes flexible chain-like structures with discrete spherical subunits of 11 nm in diameter (*R*_g_ around 3.47 nm). In the present case, the subunits are arranged in close proximity along the direction of elongation, resulting in the formation of branched worm-like nanostructures. The fit yielded an average of about 25 spherical subunits per chain, although the polydispersity is relatively high (around 0.5).

Finally, **PEG**_**45**_ **-** ***b*** **-d16GA**, which had the shortest PEG chain, formed platelet-like nanostructures with dimensions of 100 nm and a height of 6.0 ± 1.4 nm as observed by TEM and AFM (Figure 2b,e,h). Consistently, the SAXS curve showed a slope close to −2 in the low *q* region, which fits with the presence of flat nano-objects. Modeling the scattering using a disk form factor (eq S3)^27,30^ yielded an estimated thickness of 11 ± 2 nm, which correlates with the diameter of the mentioned globular subunits. Therefore, it can be assumed that these platelets are also formed by a monolayer of these subunits. Further information on the SAXS analysis of the platelet units is given in the Supporting Information.

Although structurally similar, **PEG**_**45**_ **-** ***b-*** **d16U** and **PEG**_**45**_ **-** ***b-*** **d16GA** formed self-assemblies with markedly different morphologies and sizes, which could be related to differences in the dendron block crystallinity. The morphology of self-assemblies formed by amorphous BCs can be tuned by factors such as hydrophobicity, molecular weight, polymer architecture, or self-assembly methodology, among others. However, self-assembly of semicrystalline BCs is strongly governed by the crystallization of the hydrophobic block, enabling the formation of complex supramolecular structures that are difficult to achieve with amorphous BCs.^31,32^ Commonly, semicrystalline BCs self-assemble into low curvature nanostructures with 2D morphologies.^33^ DSC scans of **PEG** *_**m**_* **-** ***b*** **-d16GA** after melting showed that the polymers are semicrystalline, with the dendron partially crystallizing upon cooling to RT. This tendency of the glycinamide dendron to crystallize can influence self-assembly in solution, promoting the formation of planar structures. Therefore, to determine whether the semicrystalline character of the glycinamide dendron block was affecting the self-assembly properties, X-ray diffraction (XRD) experiments were performed on the **PEG** *_**m**_* **-** ***b*** **-d16GA** nanostructures and compared to those of the **PEG**_**45**_ **-** ***b*** **-d16U** micelles (Figure S25). Thereby, the **PEG**_**45**_ **-** ***b*** **-d16U** sample displayed only a broad amorphous halo, indicative of disordered local packing and the absence of coherent short-range correlations. In contrast, glycinamide-functionalized LDBCs exhibited distinct Bragg reflections superimposed on the amorphous background, revealing the presence of crystallinity. The reflections were located at *q* values of approximately 13.6, 14.9, 16.5, and 18.5 nm^–1^, corresponding to characteristic real-space distances of 0.46, 0.42, 0.38, and 0.34 nm, respectively. The three larger spacings (0.46–0.38 nm) fell within the range of characteristic intermolecular separations commonly reported for hydrogen-bonded amide networks in polyamide systems, which are typically associated with cooperative amide–amide correlations.^34^ Importantly, these reflections did not match the characteristic crystalline reflections of PEG, confirming that the PEG blocks remained noncrystalline in all self-assemblies studied. A clear dependence on PEG length was also observed as **PEG**_**45**_ **-** ***b*** **-d16GA** displayed a dominant reflection at *q* ≈ 14.9 nm^–1^, while increasing the PEG length to **PEG**_**113**_ and **PEG**_**226**_ led to broader and redistributed features. The unusually high intensity of the reflection in **PEG**_**45**_ **-** ***b*** **-d16GA** should be interpreted with caution, as the XRD measurements were performed on deposited samples and, as indicated by SAXS and TEM results, this copolymer assembles into platelet-like structures, which may give rise to preferential orientation effects and an associated enhancement of selected diffraction intensities.

From these results, it can be inferred that the rigidity of the semicrystalline glycinamide dendritic segment likely reduces self-assembly curvature and disfavors the formation of micelles or other curved structures.^35^ For **PEG**_**45**_ **-** ***b*** **-d16GA**, this might result in the stacking of crystalline domains into flat platelets, where the short PEG segment is insufficient to stabilize the structures, ultimately leading to precipitation. Increasing the length of the PEG chains may hinder crystallization of the dendritic segment, and the self-assembled morphologies evolved from nanoplatelets to branched worm-like nanostructures in concordance with the literature.^33,36,37^ Conversely, **PEG**_**45**_ **-** ***b*** **-d16U**, with an amorphous hydrophobic block having a *T*_g_ below RT, forms spherical micelles with a liquid-like core.

### Thermoresponsive Behavior of Ureido-Functionalized Dendritic Macromolecules

The model **C**_**6**_ **-d16U** dendron was insoluble in water over the temperature range from 20 to 90 °C. However, the micellar dispersion of **PEG**_**45**_ **-** ***b*** **-d16U** at 1.0 mg mL^–1^ visually underwent a nearly transparent-to-turbid transition upon heating. The transmittance curve displayed a sharp drop at a *T*_cp_ of 54 °C upon heating and recovery upon cooling, exhibiting a small hysteresis of 3–5 °C that remained reproducible over successive cycles (Figures 3a and S26). DLS measurements revealed a pronounced increase in the average *D*_h_ above *T*_cp_, reaching values above 750 nm (PDI ≥ 0.25) (Figure 3b), which is consistent with the formation of large spherical aggregates observed in the TEM images of a 0.5 mg mL^–1^ dispersion at 70 °C (average diameter of 220 ± 107 nm, Figure 3c). SAXS measurements collected between 20 and 80 °C showed an increase in the scattering intensity with an almost continuous slope in the low-*q* region above 40 °C, which also indicates the presence of large aggregates with dimensions exceeding the resolution of the SAXS experiments (Figure 3d). Upon cooling, the spherical micelles were recovered, as evidenced by the presence of a small slope region at low-*q* and a kink at around 0.6 nm^–1^.

**Figure 3:**
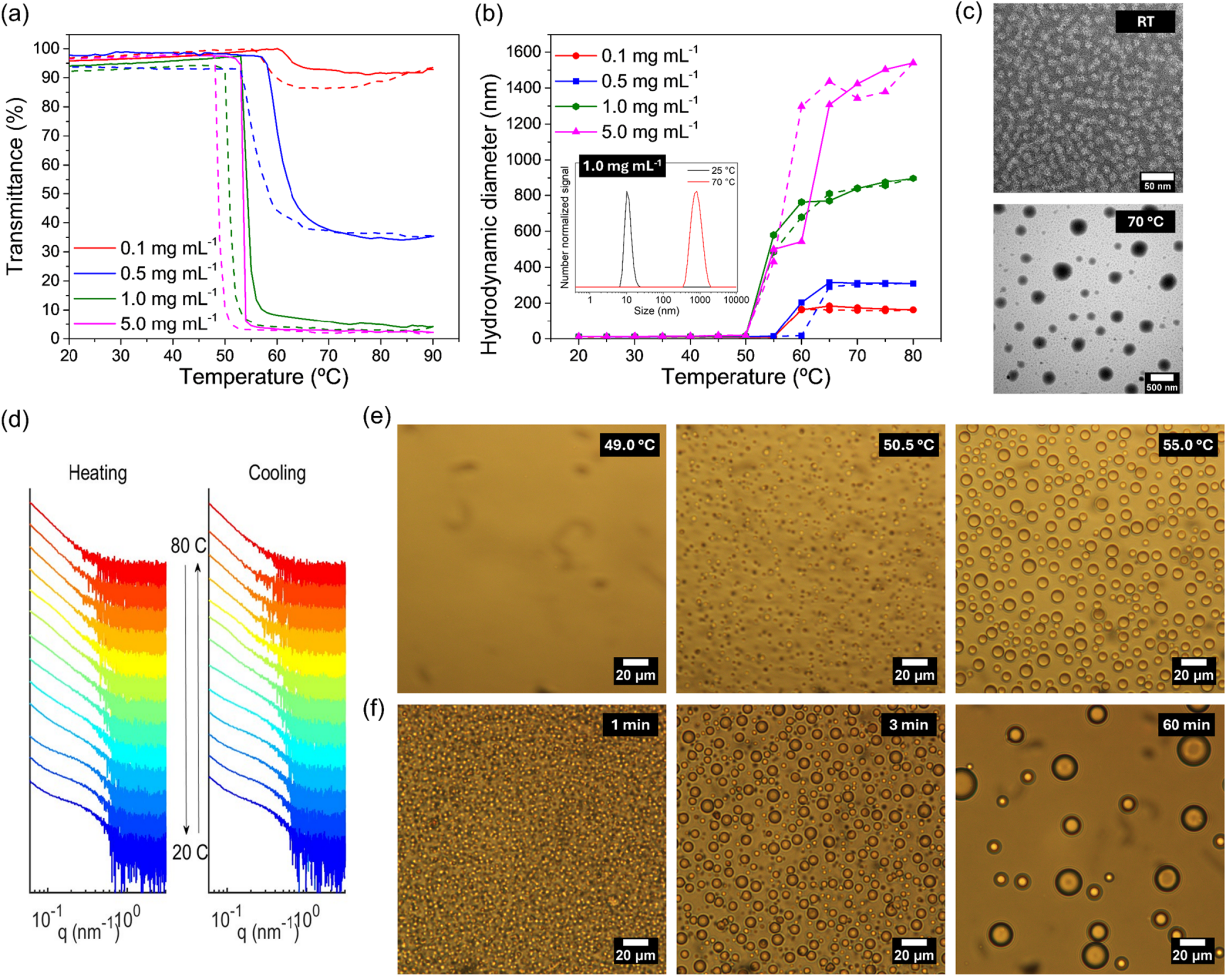
(a) Temperature-dependent transmittance curves and (b) temperature evolution of number-average *D*_h_ for **PEG**_**45**_ **-** ***b*** **-d16U** dispersions in water at different polymer concentrations upon first heating (solid line) and first cooling (dashed line). Inserted DLS number size distributions in water at 1.0 mg mL^–1^. (c) TEM images of **PEG**_**45**_ **-** ***b*** **-d16U** self-assemblies at RT (stained with uranyl acetate) and 70 °C (stained with phosphotungstic acid). (d) SAXS patterns from **PEG**_**45**_ **-** ***b*** **-d16U** at 1.0 mg mL^–1^ recorded on heating and subsequent cooling between 20 and 80 °C, with the measurements collected at 5 °C intervals. (e) Optical microscopy photographs of **PEG**_**45**_ **-** ***b*** **-d16U** at 5.0 mg mL^–1^ upon heating from 48 to 65 °C at 0.5 °C min^–1^ and (f) maintained at 60 °C for varying times.

*T*_cp_ and *D*_h_ were also measured as a function of polymer concentration. Increasing the concentration of **PEG**_**45**_ **-** ***b*** **-d16U** reduced *T*_cp_ from 62 °C at 0.1 mg mL^–1^ to 53 °C at 5.0 mg mL^–1^ (Figure 3a), while the aggregate size increased (Figure 3b). Above 1.0 mg mL^–1^, the aggregates reached the micrometer scale, whereas *T*_cp_ remained nearly constant. Overall, **PEG**_**45**_ **-** ***b*** **-d16U** showed a reversible thermoresponsive behavior, although it did not correspond to a typical UCST transition.

The thermoresponsive behavior of **PEG**_**45**_ **-** ***b*** **-d16U** was studied in phosphate buffer saline (PBS, 1×, pH 7.4) at a 1.0 mg mL^–1^ (Figure S27). Upon heating, the sharp decrease in transmittance occurred at *T*_cp_ of 47 °C, a temperature slightly lower than that measured in water at the same concentration. This transition was followed by a gradual increase in transmittance, which is associated with polymer precipitation at the bottom of the cuvette, likely induced by the salting-out effect of HPO_4_^2–^ ions.^38,39^ The thermoresponsive behavior was reversible on cooling, with the dispersion recovering its transparency at 40 °C.

Aqueous dispersions of **PEG**_**45**_ **-** ***b*** **-d16U** with concentrations in the range 5.0–10.0 mg mL^–1^ were further studied by optical microscopy (OM) and ^1^H NMR spectroscopy. OM revealed a homogeneous single phase below *T*_cp_, whereas heating above *T*_cp_ induced the formation of microdroplets (1–2 μm) that grew and fused upon further heating (Figures 3e and S28a and Video S1). Maintaining the sample at 60 °C confirmed their liquid-like behavior (Figures 3f and S28b). These observations indicated a reversible liquid–liquid phase separation or coacervation on heating above *T*_cp_. This liquid–liquid phase separation was also investigated by variable temperature ^1^H NMR spectroscopy in D_2_O using pyridine in C_2_D_2_Cl_4_ as an external standard. At 20 °C, the PEG signals (labeled as *a* and *b*) were well resolved, while those of the ureido dendritic block appeared broad and less intense, reflecting micellar self-assembly, with the dendritic block forming the micellar core shielded from the solvent, and the PEG chains forming a well-solvated shell (Figure 4a). As the temperature increased, the dendritic block signals became gradually sharper and more intense, indicating enhanced mobility and greater exposure of the micellar core to the aqueous medium, resulting from an increase in dendron hydrophilicity. Notably, above *T*_cp_, the PEG signal (labeled as *b*) splits into two, suggesting alterations in the local polarity or subtle interactions between the hydrophobic and hydrophilic blocks during liquid–liquid phase separation.

**Figure 4:**
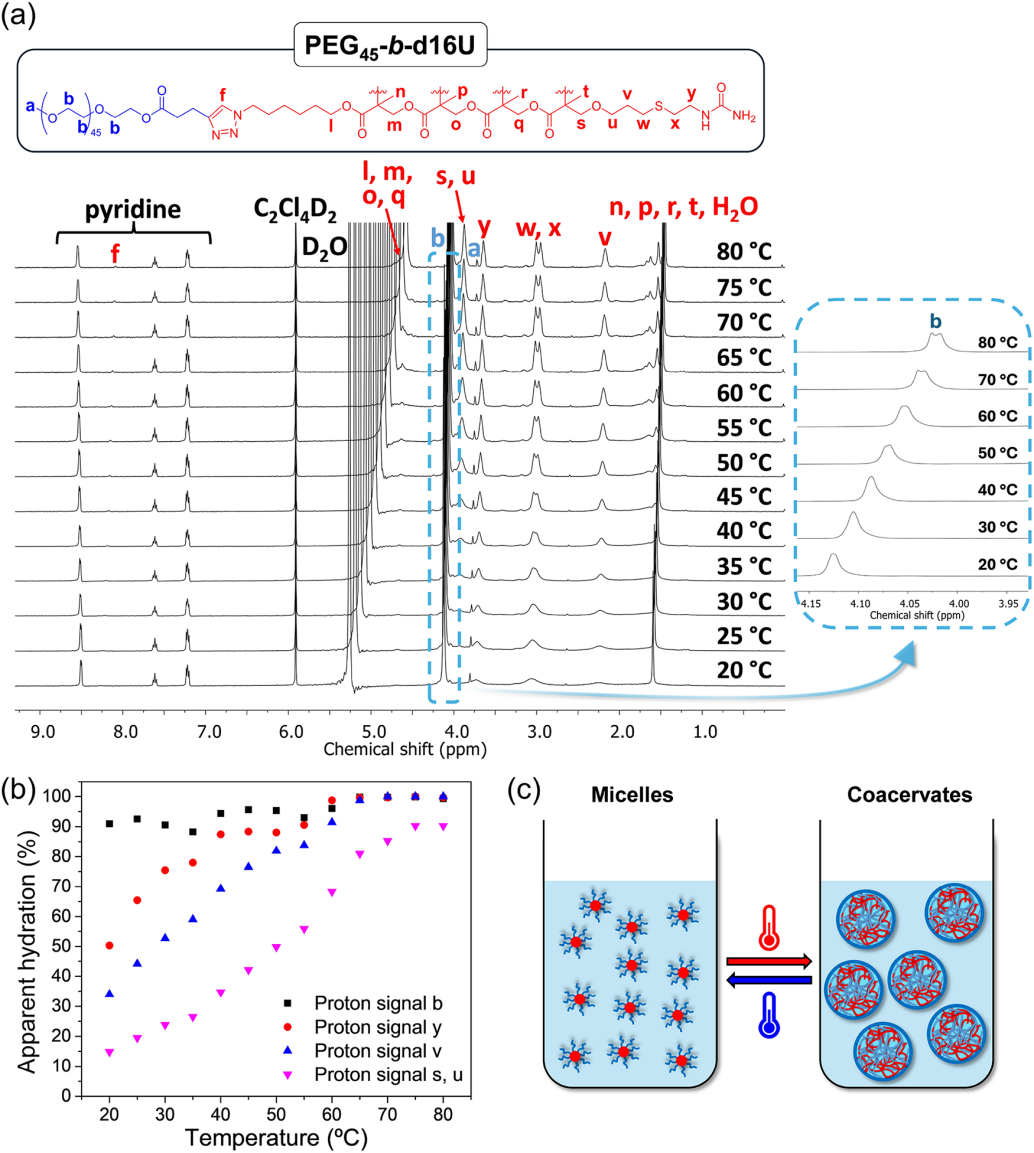
(a) ^1^H NMR (400 MHz, D_2_O) spectra of **PEG**_**45**_-***b*-d16U** (5.0 mg mL^–1^) concentration upon heating, using pyridine as an external standard. (b) Temperature-dependent evolution of the NMR normalized intensities in D_2_O of selected signals relative to normalized intensities in DMSO-*d*_6_ of **PEG**_**45**_-***b*** **-d16U** upon heating. (c) Schematic representation of the thermoresponse of **PEG**_**45**_-***b*** **-d16U** micelles in water promoted by the increased hydrophilicity of the ureido-functionalized dendritic block upon heating.

These changes in resonance intensities were quantified in terms of variations in the apparent degree of hydration of selected protons, determined by comparing the peak integrations in D_2_O at a given temperature with those in DMSO-*d*_6_, where the polymer is fully dissolved and the hydration degree is taken as 100% (Figure 4b).^40^ The methylenic protons of the PEG segments displayed consistently high apparent hydration degrees (90–100%) across the entire temperature range, despite the signal splitting observed above 50 °C, indicating that PEG remains well solvated throughout. In contrast, the apparent hydration of the dendritic block at 20 °C depended on the proton position. Methylenic protons at the α position of the ureido group (labeled as *y*) exhibited the highest hydration (≈50%), while more distal protons (labeled as *v* and *s* + *u*) showed lower values (34 and 15%, respectively), reflecting increasingly restricted mobility toward the dendron interior. The apparent hydration of the dendritic block increased with temperature, reaching maximum values above 60 °C, consistent with *T*_cp_ determined by turbidimetry and DLS, considering that *T*_cp_ increases in deuterated water.^41^ The hydration of the internal protons *s* + *u* occurred more slowly and at higher temperatures compared with the more external protons *y* and *v*. These results confirm that coacervation or phase separation above *T*_cp_ is accompanied by extensive hydration of the dendron segment, which initially forms the micellar core. This behavior is similar to that observed in amphiphilic BCs functionalized with ureido groups described in previous work,^11^ in which the hydrophobic chains become increasingly hydrated upon increasing temperature without achieving complete solubilization (Figure 4c).^23^

### Thermoresponsive Behavior of Glycinamide-Functionalized Dendritic Macromolecules

Unlike the **C**_**6**_ **-d16U**, the glycinamide **C**_**6**_ **-d16GA** model dendron was soluble in water upon heating, showing typical UCST behavior. Phase transition was sharp, with a *T*_cp_ of 80 °C and a broad hysteresis of 16 °C between heating and cooling at polymer concentration of 1.0 mg mL^–1^ (Figures 5a and S29a). The different water solubility of both model dendrons (**C**_**6**_ **-d16U** vs **C**_**6**_ **-d16GA**) upon heating can be explained in terms of hydrophobicity. The calculated log *P* values of ureido-thiol (−0.74) and glycinamide-thiol (−1.68) compounds indicated that the glycinamide is more hydrophilic. This higher hydrophilicity likely accounts for the enhanced solubility of the model **C**_**6**_ **-d16GA** dendron and consequently facilitates UCST behavior. The influence of the concentration on the thermoresponse of **C**_**6**_ **-d16GA** was examined, revealing an increase in *T*_cp_ from 75 to 83 °C, accompanied by a slight reduction in hysteresis as the dendron concentration rose from 0.1 to 5.0 mg mL^–1^ (Figure 5b). In PBS (1.0 mg mL^–1^), the behavior was essentially identical to that in water, indicating minimal salt effect (Figure S29b).

**Figure 5:**
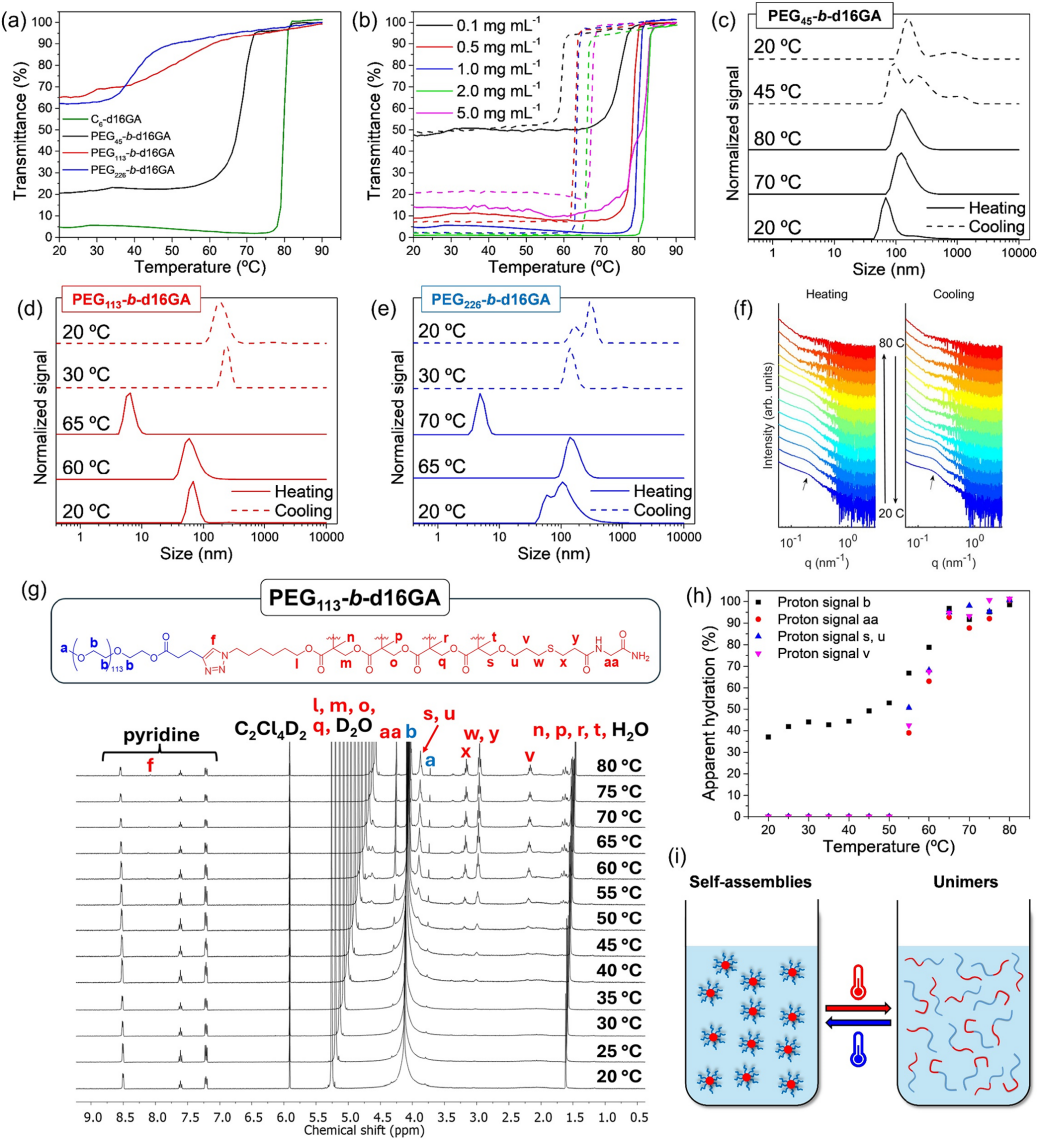
(a) Temperature-dependent transmittance curves of **C**_**6**_ **-d16GA** and **PEG** *_**m**_*-***b*** **-d16GA** (1.0 mg mL^–1^) in water upon first heating. (b) Temperature-dependent transmittance curves of **C**_**6**_ **-d16GA** at various dendron concentrations in water upon the first heating (solid line) and subsequent cooling (dashed line). (c–e) Temperature-dependent DLS number size distributions of **PEG** *_**m**_*-***b*** **-d16GA** (1.0 mg mL^–1^) in water upon heating and cooling. (f) SAXS patterns from **PEG**_**113**_-***b*** **-d16GA** (1.0 mg mL^–1^) in water upon heating and cooling between 20 and 80 °C, with the measurements collected at 5 °C intervals. (g) ^1^H NMR (400 MHz, D_2_O) spectra of **PEG**_**113**_-***b***-**d16GA** (5.0 mg mL^–1^) upon heating, using pyridine as an external standard. (h) Temperature-dependent evolution of the NMR normalized intensities in D_2_O of selected signals relative to normalized intensities in DMSO-*d*_6_ of **PEG**_**113**_-***b*** **-d16GA** upon heating. (i) Schematic representation of the thermoresponse of **PEG** *_**m**_*-***b*** **-d16GA** self-assemblies in water promoted by the UCST behavior of the dendritic block.

**PEG** *_**m**_* **-** ***b*** **-d16GA** LDBCs also exhibited typical UCST behavior, but the incorporation of the PEG block markedly lowered *T*_cp_ compared to **C**_**6**_ **-d16GA**, reflecting the hydrophilicity provided by the PEG segment (Figures 5a and S30). Upon first heating, **PEG**_**45**_ **-** ***b*** **-d16GA**, with the shortest PEG block, showed a sharp phase transition at 68 °C. Consequently, as the PEG length increased, *T*_cp_ decreased to 53 °C for **PEG**_**113**_ **-** ***b*** **-d16GA** and 39 °C for **PEG**_**226**_ **-** ***b*** **-d16GA**.^42,43^ In addition, LDBCs with longer PEG blocks exhibited a more gradual phase transition and a smaller change in transmittance. All of these glycinamide-functionalized LDBCs exhibited a pronounced hysteresis of 20–25 °C, larger than that of **C**_**6**_ **-d16GA**, suggesting that the hydrophilic PEG blocks hinder hydrogen bonding between glycinamide groups.^42^ This behavior was reversible with reproducible transitions across cycles except for the first heating scan (Figure S30), which could be due to variations in self-assembly that can affect the crystallinity of the dendritic block, influencing size, morphology, and final transmittance.^32,36^ To account for this effect, TEM images were acquired from the self-assembled samples after completion of the first heating–cooling cycle. **PEG**_**45**_ **-** ***b*** **-d16GA** self-assemblies exhibited no appreciable morphological changes, whereas those derived from **PEG**_**113**_ **-** ***b*** **-d16GA** and **PEG**_**226**_ **-** ***b*** **-d16GA** reorganized into narrower, more elongated, and shorter nanostructures (Figure S31).

DLS measurements of **PEG**_**45**_ **-** ***b*** **-d16GA** showed negligible changes in average *D*_h_ with temperature (Figure 5c), although the derived count rate, representing the scattered light intensity, decreased sharply and reversibly above 60 °C, coincident with the decrease in turbidity seen in transmittance curves (Figure S32a). This likely reflects a gradual, although incomplete, disassembly of self-assemblies into individual macromolecules or unimers upon heating, as reported by Doncom et al.^43^ In contrast, **PEG**_**113**_ **-** ***b*** **-d16GA** and **PEG**_**226**_ **-** ***b*** **-d16GA** fully solubilized into unimers species (*D*_h_ = 5 ± 1 nm) above 60 and 65 °C, respectively (Figure 5d,e), temperatures significantly higher than *T*_cp_ determined from transmittance. Nevertheless, the derived count rate decreased above 40 °C for **PEG**_**113**_ **-** ***b*** **-d16GA** and 30 °C for **PEG**_**226**_ **-** ***b*** **-d16GA** due to progressive solubilization of polymer self-assemblies, which is more consistent with the transmittance curves (Figure S32b,c). Upon cooling, the reconstitution of self-assemblies occurred around 30 °C.

**PEG**_**113**_ **-** ***b*** **-d16GA** was selected for a detailed study of its thermoresponsive behavior. In concordance with the transmittance curves and DLS measurements, the SAXS profile of **PEG**_**113**_ **-** ***b*** **-d16GA** showed that the fractal aggregates persisted up to temperatures of approximately 55 °C. Beyond this temperature, the kink marked with an arrow in Figure 5f, which is the signature of these aggregates, disappeared. Concurrently, the SAXS signal became highly noisy and difficult to analyze, resembling that of pure water, which indicates LDBC solubilization. Upon cooling to below 30 °C, the fractal kink was recovered at nearly the same position as before heating, indicating that the LDBC unimers reaggregate into large self-assemblies.

The effect of polymer concentration was assessed, revealing a gradual increase in *T*_cp_ upon increasing the concentration from 0.1 to 5.0 mg mL^–1^, as expected for UCST polymers (Figure S33).^44,45^ Variable temperature ^1^H NMR experiments in D_2_O were also conducted (Figures 5g,h and S34). At 20 °C, all dendritic resonances were suppressed, and the PEG ones were markedly reduced, corresponding to a hydration degree of 37% for signal *b*. The absence of dendritic signals below the *T*_cp_ might be due to the crystallization of the dendritic block, as observed in the bulk material and XRD measurements. Crystallization of the dendron should also restrict the mobility of the PEG segment, which in turn would cause the substantial attenuation of its signals. When the polymer dispersion was heated above 50 °C, all peaks became more defined and intense, reaching a maximum at 65 °C, where the apparent hydration degree exceeded 90% (Figure 5g,h). Upon cooling, all changes abruptly reverted at 35 °C (Figure S34). The temperatures and changes observed by NMR are in good agreement with those determined from transmittance and DLS measurements, reflecting self-assembly disintegration and polymer solubilization (Figure 5i).

The thermoresponse of **PEG**_**113**_ **-** ***b*** **-d16GA** was also studied in PBS (pH 7.4) at a concentration of 1.0 mg mL^–1^, showing behavior comparable to that in water, with a *T*_cp_ of 53 °C. Upon heating, the self-assemblies fully disassembled into unimers at 65 °C, and they reassembled approximately 30 °C upon cooling (Figure S35).

### Preliminary Studies on Nile Red Encapsulation and Release, and Cytotoxicity Evaluation of LDBCs

The ability of these self-assemblies to encapsulate and thermally trigger the release of hydrophobic cargo was preliminarily investigated using Nile Red, a polarity-sensitive fluorescence probe, with **PEG**_**45**_ **-** ***b*** **-d16U** and **PEG**_**113**_ **-** ***b*** **-d16GA** LDBCs selected as representative examples. Nile Red was encapsulated within the self-assemblies dispersed in water at a polymer concentration of 1.0 mg mL^–1^, and its emission was recorded every 5 °C upon heating and cooling between 20 and 80 °C (Figures 6a–d and S36). At 20 °C, both **PEG**_**45**_ **-** ***b*** **-d16U** and **PEG**_**113**_ **-** ***b*** **-d16GA** self-assemblies encapsulated Nile Red, as deduced from the strong emission recorded, with maxima at 628 and 622 nm, respectively. Upon heating, a decrease in the emission accompanied by a slight red shift of emission maxima (635 nm for **PEG**_**45**_ **-** ***b*** **-d16U** and 640 nm for **PEG**_**113**_ **-** ***b*** **-d16GA**) occurred above the *T*_cp_. As reported in the literature, the decrease in fluorescence intensity and the red shift of the emission maxima are attributed to the localization of Nile Red within a more polar environment.^46,47^ Upon cooling, the initial emission was recovered in both cases. Therefore, considering the UCST behavior of **PEG**_**113**_ **-** ***b*** **-d16GA**, it can be presumed that disassembly and solubilization of **PEG**_**113**_ **-** ***b*** **-d16GA** above *T*_cp_ triggered the release of Nile Red into the more polar aqueous environment. During the cooling process, **PEG**_**113**_ **-** ***b*** **-d16GA** reassembled, allowing the self-assemblies to re-encapsulate Nile Red. As a consequence, the fluorescence intensity increased back to the initial values and the emission maxima shifted toward blue wavelengths (Figure S36). Changes in emission were less pronounced for **PEG**_**45**_ **-** ***b*** **-d16U**. As it has been concluded, in this case, the micelles do not dissolve; instead, phase separation or coacervation is driven by micellar hydration and collapse. For **PEG**_**45**_ **-** ***b*** **-d16U**, coacervate domains might either partially retain Nile Red or increase local polarity, both of which can reduce fluorescence intensity.

**Figure 6:**
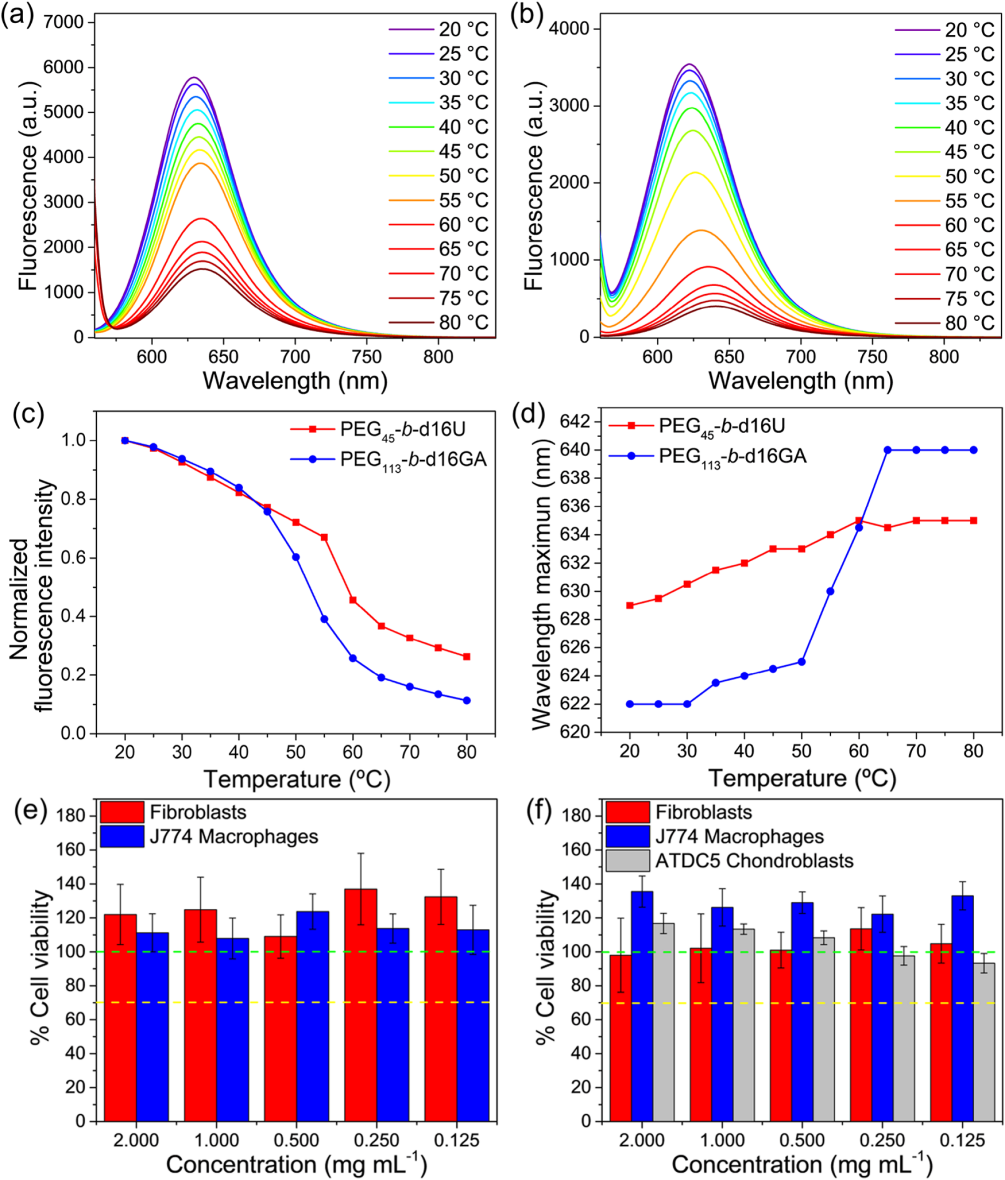
Variable temperature emission spectra at 1.0 mg mL^–1^ of (a) **PEG**_**45**_-***b*** **-d16U** and (b) **PEG**_**113**_-***b*** **-d16GA** dispersion loaded with 1.0 × 10^–6^ M of Nile Red (λexc. = 550 nm) upon heating. (c) Temperature-dependent evolution of the normalized maximum fluorescence intensity of Nile Red upon heating. (d) Temperature-dependent evolution of the wavelength maximum of Nile Red upon heating. Graphical representation of viability values obtained through metabolic activity assays after 24 h exposure to (e) **PEG**_**45**_-***b*** **-d16U** and (f) **PEG**_**113**_-***b*** **-d16GA** self-assemblies in different cell lines. The yellow dashed line indicates 70% cell viability, which is the threshold for cytotoxicity as defined by ISO 10993–5.^48^ The green dashed line represents 100% viability and served as the statistical reference.

Cytocompatibility of the LDBCs is a key requirement for employing their self-assemblies in biomedical applications. Therefore, the cytotoxicity of **PEG**_**45**_ **-** ***b*** **-d16U** and **PEG**_**113**_ **-** ***b*** **-d16GA** LDBCs was evaluated in human dermal fibroblast and J774A.1 mouse monocyte macrophages. The viability was assessed at LDBC concentrations ranging from 0.125 to 2.000 mg mL^–1^. Following the ISO 10993–5:2009 standard,^48^ LDBCs were considered cytotoxic at a given concentration if cell viability was below 70%. As shown in Figure 6e,f, neither of the two LDBCs exhibited cytotoxic effects on fibroblasts and macrophages across all tested concentrations. For **PEG**_**113**_ **-** ***b*** **-d16GA** self-assemblies, cell viability was further studied in chondrogenic ATDC5 cells, which also demonstrated good cytocompatibility at 2.000 mg mL^–1^ (Figure 6f). Besides, the cell cycle analysis of J774A.1 macrophages was performed for **PEG**_**113**_ **-** ***b*** **-d16GA** self-assemblies to evaluate whether the polymer induced apoptosis, altered cell cycle phase distribution, affected cell division, or impeded cell growth. The assays were carried out at the highest and lowest polymer concentrations tested in the cytotoxicity studies. No S phase arrest, the stage of the cell cycle associated with DNA synthesis, was detected upon exposure to the LDBC self-assemblies, suggesting that these assemblies do not interfere with DNA replication or induce replication-associated cell cycle stress under the tested conditions (Figure S37).

## Conclusions

Two families of nonionic thermoresponsive amphiphilic LDBCs, one functionalized with ureido units and the other with glycinamide units at the dendron periphery, were successfully synthesized using a versatile strategy that combines two orthogonal click chemistry reactions. It was concluded that the self-assembly properties and thermoresponsive behavior of these LDBCs were dictated by the dendron’s peripheral groups. While **PEG**_**45**_ **-** ***b*** **-d16U** formed spherical micelles and worms, **PEG**_**45**_ **-** ***b*** **-d16GA** self-assembled into platelets. These significant differences in the morphology and size of the self-assemblies were mainly attributed to the higher tendency of the glycinamide dendron toward crystallization compared with the ureido analogue, which hampered the formation of curved self-assembled structures. By increasing the PEG chain length, which enhanced the hydrophilic to hydrophobic balance and restricted the tendency of the glycinamide dendron to crystallize, the self-assemblies transitioned from platelet structures to more curved morphologies, such as branched worm-like nanostructures. Regarding the thermoresponsive behavior, glycinamide was a more effective UCST promoter than ureido, likely because of its higher hydrophilicity. While the ureido **C**_**6**_ **-d16U** model dendron was insoluble, the glycinamide **C**_**6**_ **-d16GA** model dendron showed UCST behavior upon heating. With respect to LDBCs, two distinct thermoresponsive behaviors were observed for the ureido and glycinamide macromolecules. The **PEG**_**45**_ **-** ***b*** **-d16U** micelles underwent coacervation upon heating as a result of hydration of the micellar core without reaching solubilization. In contrast, **PEG** *_**m**_* **-** ***b*** **-d16GA** displayed a typical UCST response, leading to the solubilization of the self-assemblies above *T*_cp_. Moreover, the *T*_cp_ decreased with an increasing PEG chain length. Overall, these results highlight that a precise linear–dendritic architecture provides a modular platform where even subtle modifications at the dendron periphery translate into pronounced differences in self-assembly and thermoresponsive behavior in water.

Finally, the qualitative studies on encapsulation and temperature-dependent release of Nile Red, together with the good cytocompatibility of these LDBCs toward several eukaryote cell lines, provide a promising starting point for their potential application as thermoresponsive carrier platforms and for the design of new functional macromolecules.

## Supplementary Material





## Notes

Materials, characterization techniques, synthetic experimental procedures with additional NMR spectra (Figures S1–S11), description of self-assembly method, preparation of samples for XRD measurements, protocol for CAC determination, preparation of TEM samples, procedures for variable temperature ^1^H NMR and SAXS experiments, cell culture protocols, methods for cell viability and cell cycle analysis and variable temperature fluorescence measurements of Nile Red loaded micelles. Supplementary figures and tables include: FTIR spectra (Figure S12), MALDI-TOF MS spectra (Figures S13–S19), molar masses and dispersities of polymers (Table S1), TGA curves (Figure S20), DSC curves (Figure S21), CAC determination (Figure S22), DLS curves of **PEG**_**45**_ **-** ***b*** **-d16U** micelles (Figure S23), explanation of SAXS model and Guinier plot (Figure S24), XRD patterns of LDBCs, (Figure S25), temperature-dependent transmittance curves of **PEG**_**45**_ **-** ***b*** **-d16U** (Figures S26 and S27), OM photographs of **PEG**_**45**_ **-** ***b*** **-d16U** (Figure S28), temperature-dependent transmittance of **C**_**6**_ **-d16GA** and **PEG** *_**m**_* **-** ***b*** **-d16GA** (Figures S29 and S30), TEM images of **PEG** *_**m**_* **-** ***b*** **-d16GA** after first heating/cooling (Figure S31), temperature evolution of derived count rate (kcps) for **PEG** *_**m**_* **-** ***b*** **-d16GA** (Figure S32), temperature-dependent transmittance curves of **PEG**_**113**_ **-** ***b*** **-d16GA** (Figure S33), ^1^H NMR spectra of **PEG**_**113**_ **-** ***b*** **-d16GA** on cooling (Figure S34), temperature-dependent transmittance, DLS and derived count rate (kcps) of **PEG**_**113**_ **-** ***b*** **-d16GA** (Figure S35), variable temperature emission spectra of Nile Red on cooling (Figure S36) and cell cycle phase distribution (Figure S37) (PDF)
OM video of **PEG**_**45**_ **-** ***b*** **-d16U** dispersion at 5.0 mg mL^–1^ upon heating and cooling from 48 to 65 °C at 0.5 °C min^–1^ (Video S1) (MP4)
