# Supplementary material for: Peripheral Dendritic Functionalization Effects on the Self-Assembly and Thermoresponsive Behavior of Nonionic Amphiphilic Linear–Dendritic Block Copolymers in Aqueous Media
Source: Macromolecules. 2026 Jul 9;59(14):8220–33. doi: 10.1021/acs.macromol.6c00544 (PMC13435729; doi:10.1021/acs.macromol.6c00544)
Supplement: Supplementary file 1 [file ma6c00544_si_001.pdf]

Electronic Supporting Information

Peripheral Dendritic Functionalization Effects on the Self-Assembly and Thermoresponsive Behavior of Non-Ionic Amphiphilic Linear-Dendritic Block Copolymers in Aqueous Media

*Javier Martín-Martín,[1,2] Luis Folch-Cirujeda,[1,2] Cristina Yus,[1,3] Aurora Nogales,[4] Tiberio A. Ezquerra,[4] Miriam Abad,[1,2] Víctor Sebastián,[1,3,5,6] Luis Oriol,[1,2] Milagros Piñol[1,2]**

[1]Instituto de Nanociencia y Materiales de Aragón (INMA), CSIC-Universidad de Zaragoza, 50009 Zaragoza, Spain.

[2]Departamento de Química Orgánica, Facultad de Ciencias, Universidad de Zaragoza, 50009 Zaragoza, Spain.

[3]Department of Chemical Engineering and Environmental Technology, Universidad de Zaragoza, 50018 Zaragoza, Spain.

[4]Instituto de Estructura de la Materia (IEM-CSIC), 28006 Madrid, Spain.

[5]Networking Research Center on Bioengineering, Biomaterials and Nanomedicine (CIBER-BBN), 28029 Madrid, Spain.

[6]Laboratorio de Microscopías Avanzadas, Universidad de Zaragoza, 50018 Zaragoza, Spain.

Table of contents

1. Materials 3

2. Characterization techniques 3

3. Experimental procedures 6

3.1. Synthesis of **GA-SH** thiol 6

3.1. General procedure for CuAAC reaction in the preparation of **PEG_m-*b*-d16Allyl** ..9

3.2. Synthesis and characterization of **C_6-d16U** 12

3.3. Synthesis and characterization of **C_6-d16GA** 13

3.4. Synthesis and characterization of **PEG_{45}-*b*-d16U** 15

3.5. General procedure for the thiol-ene reaction in the synthesis of **PEG_m-*b*-d16GA**16

4. Self-assembly of amphiphilic LDBCs by the co-solvent method 19

5. Preparation of samples for XRD measurements 20

6. Determination of the Critical Aggregation Concentration (CAC) 20

7. TEM images 20

8. Variable temperature ^{1}H NMR experiments 21

9. Variable temperature SAXS experiments 21

10. Cell culture 21

11. Cell viability 22

12. Cell cycle 22

13. Variable temperature fluorescence measurements of Nile Red loaded micelles 23

14. Supplementary figures and tables 23

1. Materials

The **U-SH** thiol was synthesized as reported in the literature.[1] Alkyne-functionalized PEG (**PEG_{45}-Alkyne**, **PEG_{113}-Alkyne** and **PEG_{226}-Alkyne**) were synthesized according to previously described methods.[2] The **N_3-d16Allyl** and **C_6-d16Allyl** was synthesized according to reported procedures.[3] Poly(ethylene glycol) methyl ethers (**PEG_{45}-OH**, **PEG_{113}-OH** and **PEG_{226}-OH**) were purchased from Sigma-Aldrich. The number average molar mass (M_n) provided by the supplier was confirmed via MALDI-TOF mass analysis using Polytools Bruker software, M_n = 1942, 5119 and 10262 g mol^{-1}, respectively. The dispersities (*Đ*) were 1.02, 1.04 and 1.04, respectively, determined by size exclusion chromatography (SEC) under the same conditions as those used for LDBCs. Phosphate buffered saline (PBS) from Gibco™ pH 7.4 (1X) was purchased from Fisher Scientific. Paraformaldehyde 4% in PBS was obtained from Alfa Aesar (Germany). Dulbecco's Phosphate Buffered Saline (DPBS) and Dulbecco's modified Eagle's medium high glucose with stable glutamine (DMEM) and antibiotic-antimycotic solution (penicillin-streptomycin-amphotericin B; PSA) were acquired from Biowest (France). Fetal bovine serum (FBS) was purchased from Gibco (USA), and the Blue Cell viability assay kit was supplied by Abnova (Taiwan). Propidium iodide and RNAase (PI/RNASE Solution kit) was acquired by Immunostep (Spain). All other reagents and solvents were purchased from Sigma-Aldrich and Fisher Scientific and were used as received.

2. Characterization techniques

Infrared (IR) spectra were recorded on a Bruker Vertex 70 spectrophotometer using 1-2% (w/w) KBr pellets. ^{1}H NMR and ^{13}C NMR spectra were registered on a Bruker AV-400

spectrometer using either $CDCl_3$, DMSO-d_6 or D_2O. Matrix-assisted laser desorption ionization mass spectrometry (MALDI-TOF MS) was performed on an Autoflex Bruker mass spectrometer using dithranol or trans-2-[3-(4-tert-butylphenyl)-2-methyl-2-propenylidene]malononitrile (DCTB) as matrix. High resolution mass spectrometry (HRMS) was carried out in a positive ion electrospray ionization (ESI) mode on a Bruker Q-TOF-MS instrument. Size exclusion chromatography (SEC) was carried out on a Water Alliance 2695 liquid chromatography system equipped with a Waters 2424 evaporative light scattering detector using two columns connected in series, Styragel (HR2, 500 Å and HR4, 10^4 Å) from Waters (5 µm, 7.8×300 mm), and HPLC grade THF as eluent at a flow rate of 1.0 mL min^{-1} at 35 °C. Poly(methyl methacrylate) narrow molecular weight standards were used to calibrate the system. Thermogravimetric analysis (TGA) was performed at heating rate of 10 °C min^{-1} under nitrogen atmosphere on a SDT2960 Simultaneous DTA-TGA from TA instruments. Decomposition temperature associated to mass loss was read as the onset point in the weight loss curve. Differential scanning calorimetry (DSC) was performed on a DSC Q2000 calorimeter from TA instruments at scanning rate of 10 °C min^{-1}, using approx. 2.0 mg of the sample sealed in aluminium pans. The glass transition temperature (T_g) was determined at the half height of baseline jump and the melting temperature (T_m) was determined at the maximum of the corresponding peak. Turbidity measurements were conducted on a Cary 6000i UV-Vis-NIR spectrophotometer equipped with a Peltier temperature controller. The optical transmittance at 650 nm was recorded over three successive thermal cycles, by heating and then cooling from 20 °C to 90 °C at a scanning rate of 1.0 °C min^{-1}. T_{cp} was given

during the first heating scan as the temperature at which the transmittance changes 50% from the minimum and maximum of the curve. Before the transmittance measurements, the **C_6-d16GA** suspension was preheated at 50 ˚C for 5 min to facilitate the disaggregation and final dispersion of the coarse solid. Fluorescence measurements were performed on a Perkin Elmer LS 50B fluorescence spectrophotometer. The variable temperature fluorescence measurements were conducted using a Jasco F9-8550 spectrofluorometer equipped with a single-position Peltier cell holder (Jasco ETC-115). The fluorescence for assays of cell viability was measured using a Varioskan LUX microplate reader (Thermo Fisher, USA). Dynamic light scattering (DLS) measurements were carried out in a Malvern Instrument Nano ZS using a He-Ne laser with a 633 nm wavelength and a detector angle of 173 °. The size measurements were registered three times to ensure reproducibility. The standard deviation values of the hydrodynamic diameter (D_h) were obtained by fitting the corresponding size distribution to a Gaussian function. Temperature-dependent DLS was conducted from 20 °C to 80 °C at 5 °C intervals with 5 min equilibration period between each measurement, beginning with the heating scan. Optical images were visualized using an Olympus BX51 microscope equipped with an Olympus DP152 digital camera and connected to a Linkam THMS600 hot stage and a Linkam TMS94 controller. The polymeric dispersion was sandwiched in a quartz cuvette with a path length of 0.1 mm. Transmission Electron Microscopy (TEM) was carried out on a FEI TECNAI G20 electron microscope operating at 200 kV. Atomic force microscopy (AFM) analyses were conducted using a Multimode 8 microscope equipped with a Nanoscope V control unit from Bruker at a scan rate of 1.0−1.2 Hz, using Tapping

mode in ambient air. The tips employed were RTESPA-150 tip, working with a resonance frequency of 150 kHz. The samples were prepared by depositing 20 μL of 1.0 mg mL^{-1} self-assembly dispersions onto cleaved mica. After 30 s, the excess solution was removed by capillarity using a paper filter, and the samples were subsequently dried under ambient conditions. The dimensions of self-assemblies were determined as the average of 40 individual measurements. X-ray Diffraction (XRD) measurements were made using an $CuK_{\alpha 1}$ = 1.5409 A radiation (45 kV, 40 mA) on an XRD-PANanalytical Empyrean diffractometer equipped with platform Scatter X78. The calibration of beam center and the q range were carried out using the diffraction peaks of silver behenate. The 2θ range employed was from 1° to 30°, with a step size of 0.0131° and a counting time of 50 s per step. Small Angle X-ray Scattering (SAXS) experiments were performed at beamline BL11 NCD (ALBA, Spain) using a wavelength of 0.1 nm. The SAXS detector, a photon counting Dectris 2D detector (model Pilatus 1 M), was located at 3.83 m distance from the sample position. For experiments performed upon heating and cooling ramps, a capillary containing the sample was inserted into a Linkam THM600 hot stage. Log *P* (the decimal logarithm of the octanol-water partition coefficient and as a measure of hydrophobicity) of **U-SH** and **GA-SH** thiol was determined by Chemdraw professional 15.0.

3. Experimental procedures

3.1. Synthesis of GA-SH thiol

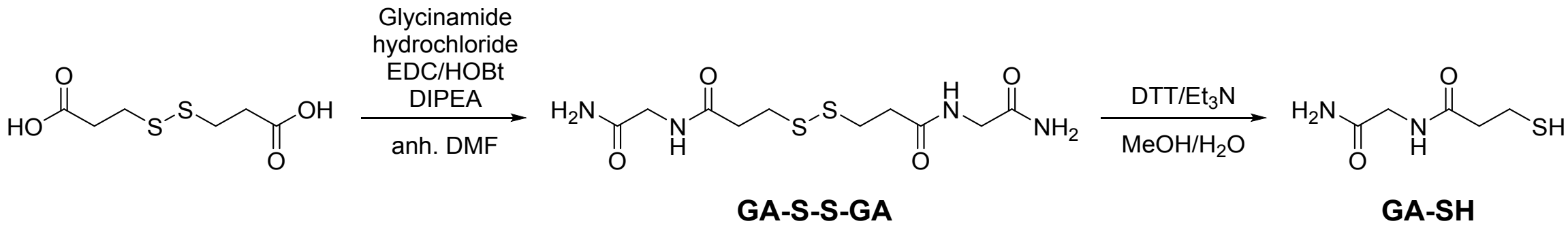

Scheme S1. Synthesis of **GA-SH**.

*Synthesis and characterization of **GA-S-S-GA**.* A solution of glycinamide hydrochloride (2.00 g, 17.73 mmol) and *N,N*-diisopropylethylamine (DIPEA) (8.38 mL, 35.42 mmol) at 0 °C in anhydrous DMF (15 mL) was prepared. Afterwards, *N*-(3-dimethylaminopropyl)-*N'*-ethylcarbodiimide hydrochloride (EDC) (2.75 g, 17.73 mmol), 1-hydroxybenzotriazole hydrate (HOBt) (2.40 g, 17.73 mmol) and finally 3,3′-dithiodipropionic acid (1.56 g, 7.38 mmol) were added in that order. After 1 h, the reaction was warmed to RT and stirred overnight under argon atmosphere. Then, the solvent was evaporated under reduced pressure. The crude was washed with DCM (3×50 mL) and filtered. The isolated solid was washed with hot ethanol (EtOH) (50 mL) and filtered to yield a white solid. Yield: 81%. FTIR (KBr), (ν, cm^{-1}): 3381, 3329, 3198 (N-H^{st}), 2938 (Csp^3-H^{st}), 1660, 1643 (C=O^{st}), 1539 (N-H^{δ}). ^1H NMR (400 MHz, DMSO-d_6) δ (ppm): 8.16 (t, *J*=5.7 Hz, 2H), 7.27 (s, 2H), 7.03 (s, 2H), 3.63 (d, *J*=5.8 Hz, 4H), 2.90 (t, *J*=7.3 Hz, 4H), 2.55 (t, *J*=7.3 Hz, 4H). ^{13}C NMR (100 MHz, DMSO-d_6) δ (ppm): 171.0, 170.4, 41.9, 34.9, 33.7.

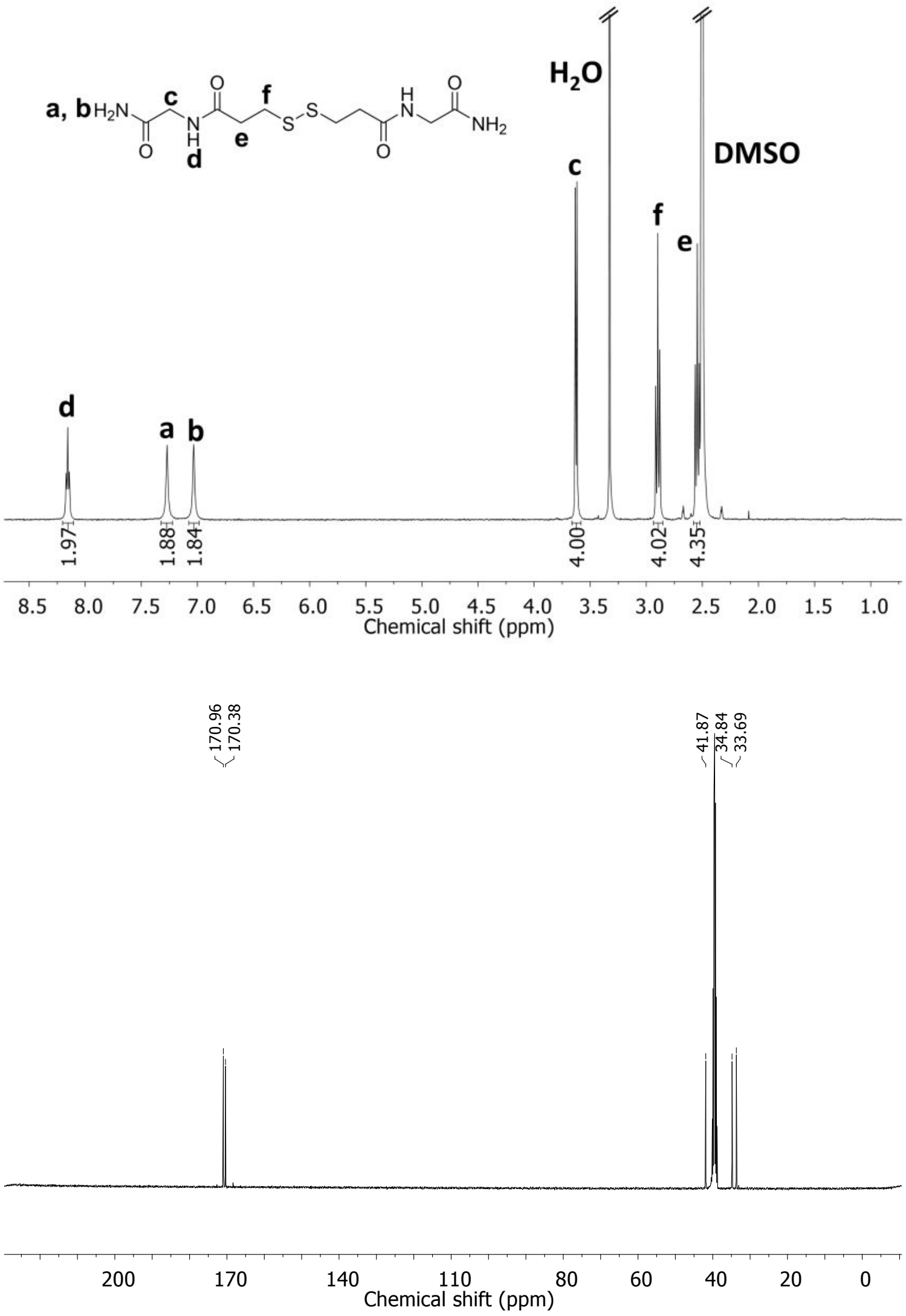

Figure S1. ^{1}H NMR (top) and ^{13}C NMR (bottom) spectra of **GA-S-S-GA** compound.

Synthesis and characterization of ***GA-SH***. A solution of **GA-S-S-GA** disulfide (1.40 g, 4.34 mmol) and *DL*-dithiothreitol (DTT) (1.06 g, 6.86 mmol) in a deoxygenated mixture of H_2O/MeOH (1:1) (80 mL) was prepared. Then, triethylamine (Et_3N) was added to the mixture until pH = 9. The reaction was stirred overnight at RT under argon atmosphere. Afterwards, the solvent was removed under reduced pressure, and the crude was purified by flash column chromatography on silica gel, using a gradient eluent from DCM/MeOH (97:3) to DCM/MeOH (8:2), to yield the **GA-SH** thiol as a white solid. Yield: 84%. FTIR

(KBr), (ν, cm^{-1}): 3393, 3313, 3201 (N-Hst), 2972, 2943 (Csp^3-Hst), 2544 (S-Hst), 1656, 1641 (C=Ost), 1548 (N-H$^{\delta}$). ^{1}H NMR (300 MHz, DMSO-d$_6$) δ (ppm): 8.08 (t, *J*=5.6 Hz, 1H), 7.25 (s, 1H), 7.01 (s, 1H), 3.63 (d, *J*=5.8 Hz, 2H), 2.71-2.60 (m, 2H), 2.45 (t, *J*=7.3 Hz, 2H), 2.36 (t, *J*=7.5 Hz, 2H). ^{13}C NMR (100 MHz, DMSO-d$_6$) δ (ppm): 171.0, 170.6, 41.8, 39.1, 19.9.

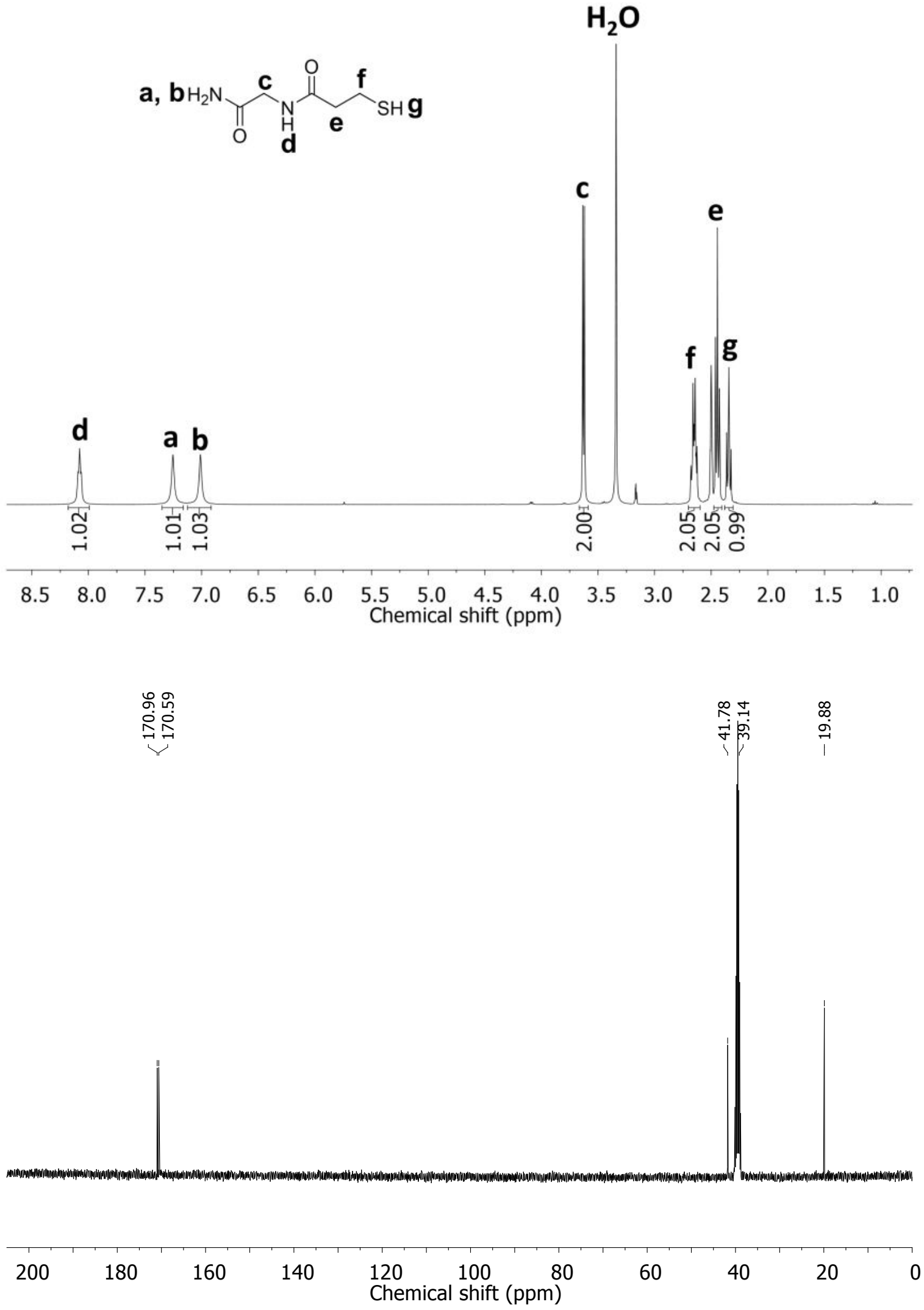

Figure S2. ^{1}H NMR (top) and ^{13}C NMR (bottom) spectra of **GA-SH** compound.

3.1. General procedure for CuAAC reaction in the preparation of PEG_m-*b*-d16Allyl

N_3-d16Allyl (1.15 mmol) and **PEG_m-Alkyne** (1.00 mmol) were dissolved in DMF (approx. 1 mL per 100.0 mg of **N_3-d16Allyl**) and the solution degassed with argon. Then, CuBr (0.20 mmol) and *N*,*N*,*N*',*N*'',*N*''-pentamethyldiethylenetriamine (PMDETA) (0.20 mmol) were added, and the reaction stirred at RT overnight. Once reacted all amount of **PEG_m-Alkyne** verified by ^{1}H NMR, the mixture was evaporated. The crude was dissolved in DCM (15 mL) and the resulting solution washed with distilled water (4×10 mL), dried over $MgSO_4$, filtered, and evaporated. The residue was purified by precipitation twice in cold hexane (50 mL) followed by decantation.

*Characterization of **PEG_{45}-b-d16Allyl**.* **PEG_{45}-*b*-d16Allyl** was synthesized according to the general procedure for CuAAC, employing the following reactants and solvents: **N_3-d16Allyl** (489.0 mg, 0.19 mmol), **PEG_{45}-Alkyne** (337.5 mg, 0.16 mmol), CuBr (4.6 mg, 0.03 mmol), PMDETA (6.8 μL, 0.03 mmol) and DMF (5 mL). The polymer was isolated as oil. Yield: 87%. FTIR (KBr), (ν, cm^{-1}): 3080 ($Csp^2–H_2^{st}$), 2979, 2937, 2870 ($Csp^3–H^{st}$), 1740 ($C{=}O^{st}$), 1646 ($C{=}C^{st}$). ^{1}H NMR (400 MHz, $CDCl_3$) δ (ppm): 7.36 (s, 1H), 5.89-5.75 (m, 16H), 5.25-5.17 (m, 16H), 5.15-5.08 (m, 16H), 4.33-4.14 (m, 32H), 4.07 (t, *J*=6.9 Hz, 2H), 3.93 (d, *J*=5.1 Hz, 32H), 3.66-3.60 (m, 178H), 3.52-3.47 (m, 32H), 3.37 (s, 3H), 3.01 (t, *J*=7.4 Hz, 2H), 2.75 (t, *J*=7.4 Hz, 2H), 1.92-1.84 (m, 2H), 1.70-1.57 (m, 2H), 1.45-1.30 (m, 4H), 1.25 (s, 3H), 1.22 (s, 6H), 1.20 (s, 12H), 1.17 (s, 24H). MALDI TOF-MS (matrix: DCTB, *m/z*): $[M+Na]^+$ Calculated for $C_{221}H_{377}O_{91}N_3Na$: 4554.5; found: 4552.6.

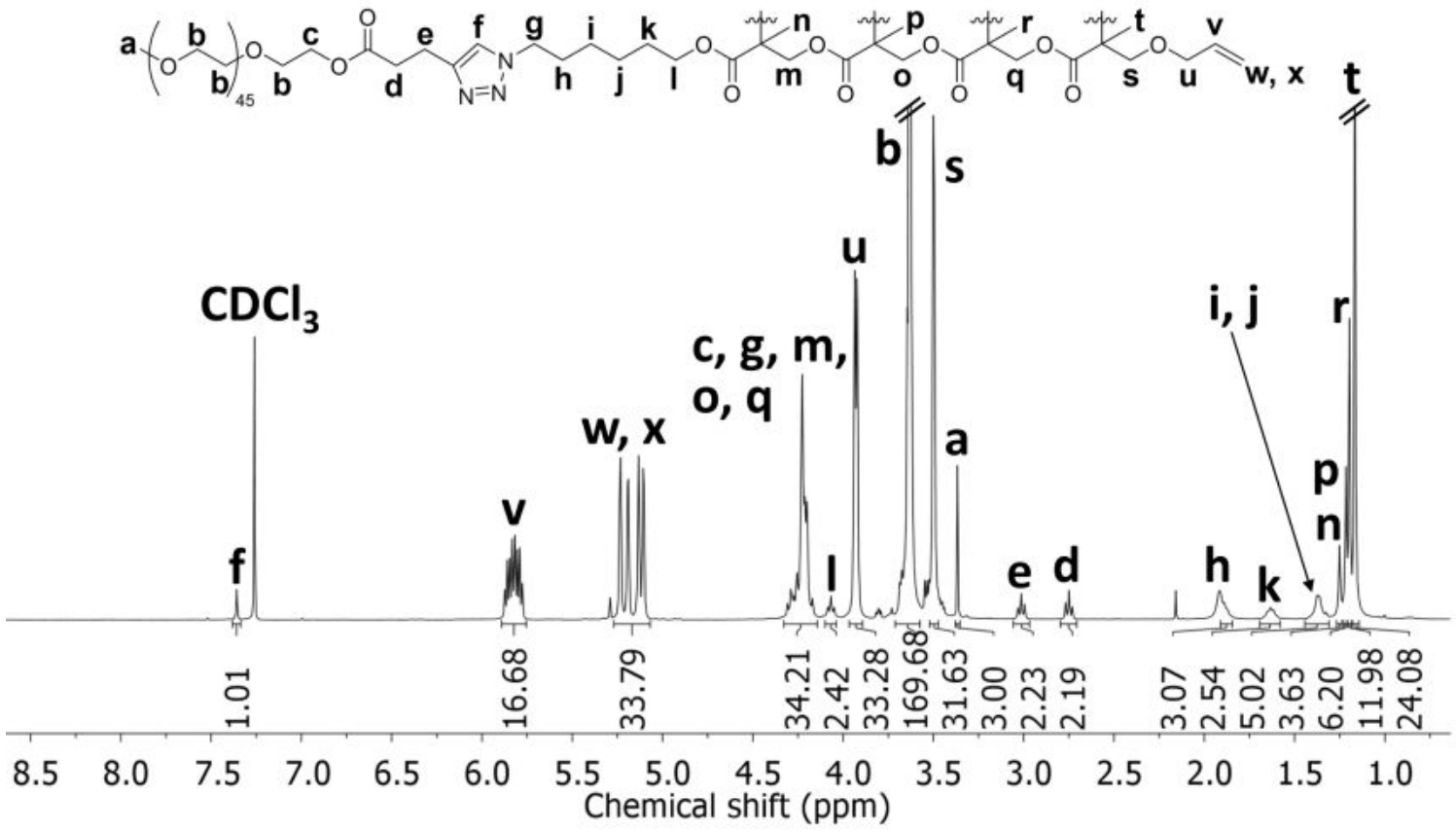

Figure S3. ^{1}H NMR spectra of **PEG$_{45}$-*b*-d16Allyl**.

*Characterization of **PEG$_{113}$-b-d16Allyl**.* **PEG$_{113}$-*b*-d16Allyl** was synthesized according to the general procedure for CuAAC, employing the following reactants and solvents: **N$_3$-d16Allyl** (100.0 mg, 0.04 mmol), **PEG$_{113}$-Alkyne** (175.1 mg, 0.03 mmol), CuBr (1.0 mg, 0.01 mmol), PMDETA (1.4 μL, 0.01 mmol) and DMF (2 mL). The polymer was isolated as a waxy solid. Yield: 81%. FTIR (KBr), (ν, cm^{-1}): 3081 (Csp^2–H$_2$st), 2979, 2946, 2886 (Csp^3–Hst), 1741 (C=Ost), 1646 (C=Cst). ^{1}H NMR (400 MHz, $CDCl_3$) δ (ppm): 7.36 (s, 1H), 5.91-5.75 (m, 16H), 5.27-5.08 (m, 32H), 4.34-4.15 (m, 32H), 4.07 (t, *J*=6.7 Hz, 2H), 3.94 (d, *J*=5.0 Hz, 32H), 3.68-3.59 (m, 450H), 3.53-3.49 (m, 32H), 3.37 (s, 3H), 3.02 (t, *J*=7.5 Hz, 2H), 2.76 (t, *J*=7.5 Hz, 2H), 1.95-1.85 (m, 2H), 1.68-1.59 (m, 2H), 1.43-1.33 (m, 4H), 1.26 (s, 3H), 1.22 (s, 6H), 1.20 (s, 12H), 1.17 (s, 24H). MALDI-TOF MS (matrix: dithranol, *m/z*): $[M+Na]^+$ Calculated for $C_{361}H_{657}O_{161}N_3Na$: 7638.3; found: 7638.9.

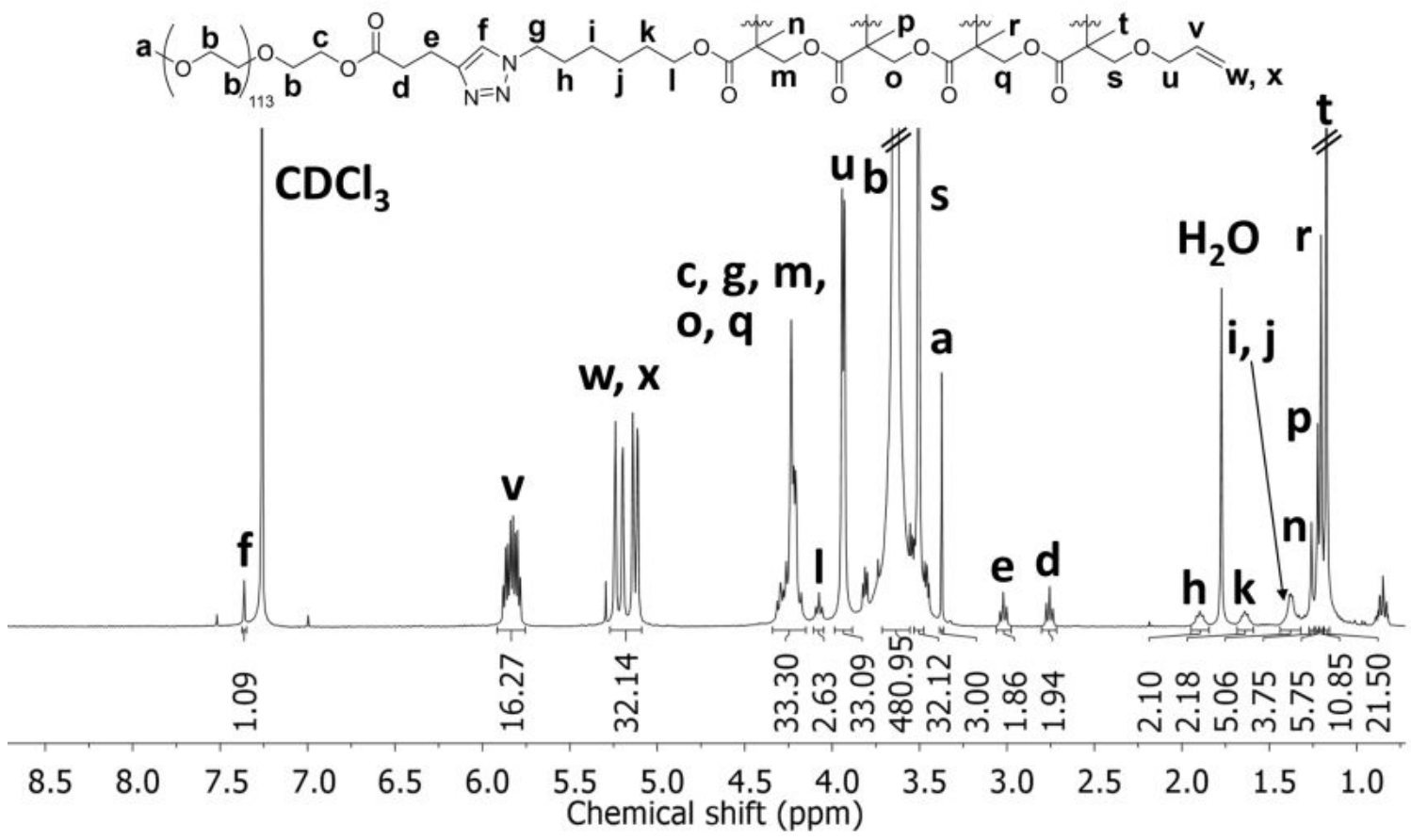

Figure S4. ^{1}H NMR spectra of **PEG$_{113}$-*b*-d16Allyl**.

*Characterization of **PEG$_{226}$-b-d16Allyl**.* **PEG$_{226}$-*b*-d16Allyl** was synthesized according to the general procedure for CuAAC, employing the following reactants and solvents: **N$_3$-d16Allyl** (100.0 mg, 0.04 mmol), **PEG$_{226}$-Alkyne** (346.2 mg, 0.03 mmol), CuBr (1.0 mg, 0.01 mmol), PMDETA (1.4 μL, 0.01 mmol) and DMF (2 mL). The polymer was isolated as a waxy solid. Yield: 89%. FTIR (KBr), (ν, cm^{-1}): 3081 (Csp^2–H$_2$st), 2946, 2886, 2803 (Csp^3–Hst), 1740 (C=Ost), 1646 (C=Cst). ^{1}H NMR (400 MHz, CDCl$_3$) δ (ppm): 7.36 (s, 1H), 5.91-5.75 (m, 16H), 5.27-5.07 (m, 32H), 4.32-4.16 (m, 32H), 4.07 (t, *J*=6.9 Hz, 2H), 3.93 (d, *J*=5.5 Hz, 32H), 3.69-3.57 (m, 900H), 3.52-3.48 (m, 32H), 3.37 (s, 3H), 3.02 (t, *J*=7.3 Hz, 2H), 2.75 (t, *J*=7.3 Hz, 2H), 1.92-1.85 (m, 2H), 1.69-1.59 (m, 2H), 1.42-1.33 (m, 4H), 1.26 (s, 3H), 1.22 (s, 6H), 1.20 (s, 12H), 1.17 (s, 24H). MALDI-TOF MS (matrix: dithranol, *m/z*): [M+Na]$^+$ Calculated for $C_{595}H_{1125}O_{278}N_3Na$: 12792.4; found: 12784.7.

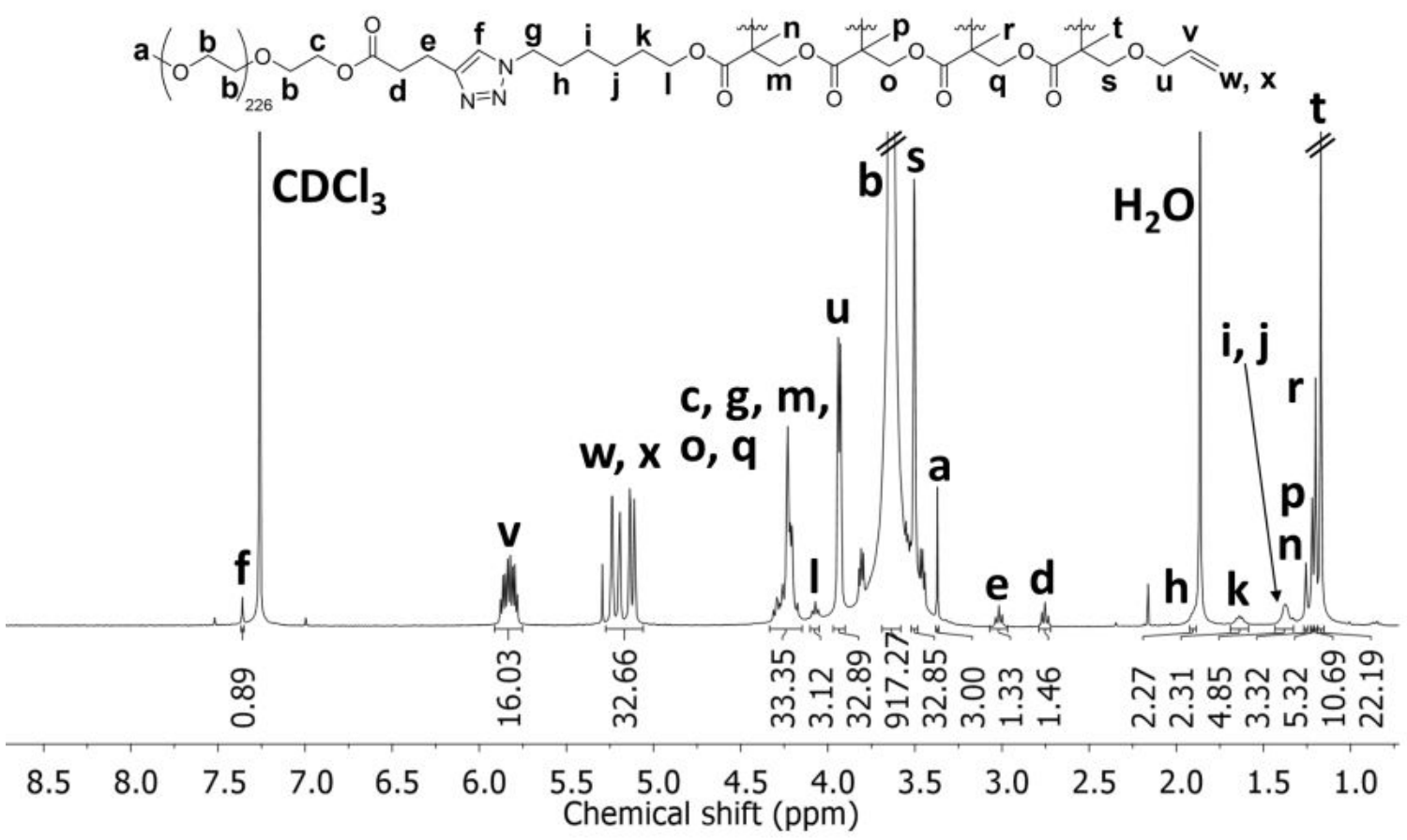

Figure S5. ^{1}H NMR spectra of **PEG$_{226}$-*b*-d16Allyl**.

3.2. Synthesis and characterization of C$_6$-d16U

2,2-Dimethoxy-2-phenyl acetophenone (DMPA) (15.6 mg, 0.06 mmol) was added to a solution of **U-SH** thiol (294.4 mg, 2.45 mmol) and **C$_6$-d16Allyl** (200.0 mg, 0.07 mmol, 1.22 mmol of alkene groups) in deoxygenated DMF (5 mL). Under argon atmosphere, the reaction was stirred at RT inside of a chamber equipped with interior mirrors and irradiated by a Philips PL-S-9W UV Hg lamp with a maximum emission at 365 nm, for 6 h, until complete functionalization was confirmed by ^{1}H NMR. Afterwards, the reaction was precipitated into cold diethyl ether and the supernatant was decanted. The precipitate was washed with DCM (3×10 mL), then with THF (3×10 mL) and finally with Milli-Q® water (3×10 mL). The solid was dried under vacuum to yield **C$_6$-d16U** as a white powdery solid. Yield: 68%. FTIR (KBr), (ν, cm^{-1}): 3455, 3344, 3218 (N–Hst), 2934, 2870 (Csp^{3}–Hst), 1738, 1655 (C=Ost), 1604 (NH$_2$$^{\delta}$), 1549 (N–H$^{\delta}$). ^{1}H NMR (400 MHz, DMSO-d$_6$) δ (ppm): 7.77 (s, 1H), 6.07 (t, *J*=5.5 Hz, 16H), 5.50 (s broad, 16H), 4.35-3.97 (m, 32H), 3.48-3.36 (m, 64H), 3.12 (q, *J*=6.9 Hz, 32H), 2.59 (t, *J*=6.8 Hz, 2H), 2.54-2.47 (m, 64H), 1.84-1.76 (m, 2H), 1.70 (quint, *J*=6.7 Hz, 32H), 1.61-1.50 (m, 4H), 1.37-1.27 (m,

6H), 1.24-1.18 (m, 9H), 1.15 (s, 12H), 1.07 (s, 24H), 0.89 (t, *J*=7.2 Hz, 3H). ^{13}C NMR (100 MHz, DMSO-d_6) δ (ppm): 173.0, 171.7, 171.5, 171.2, 158.5, 146.8, 121.4, 71.9, 69.2, 65.3, 64.8, 64.4, 49.0, 47.9, 46.3, 39.2, 31.7, 31.6, 31.1, 30.6, 29.1, 27.8, 27.6, 25.5, 24.7, 21.7, 18.3, 17.5, 16.9, 13.7.

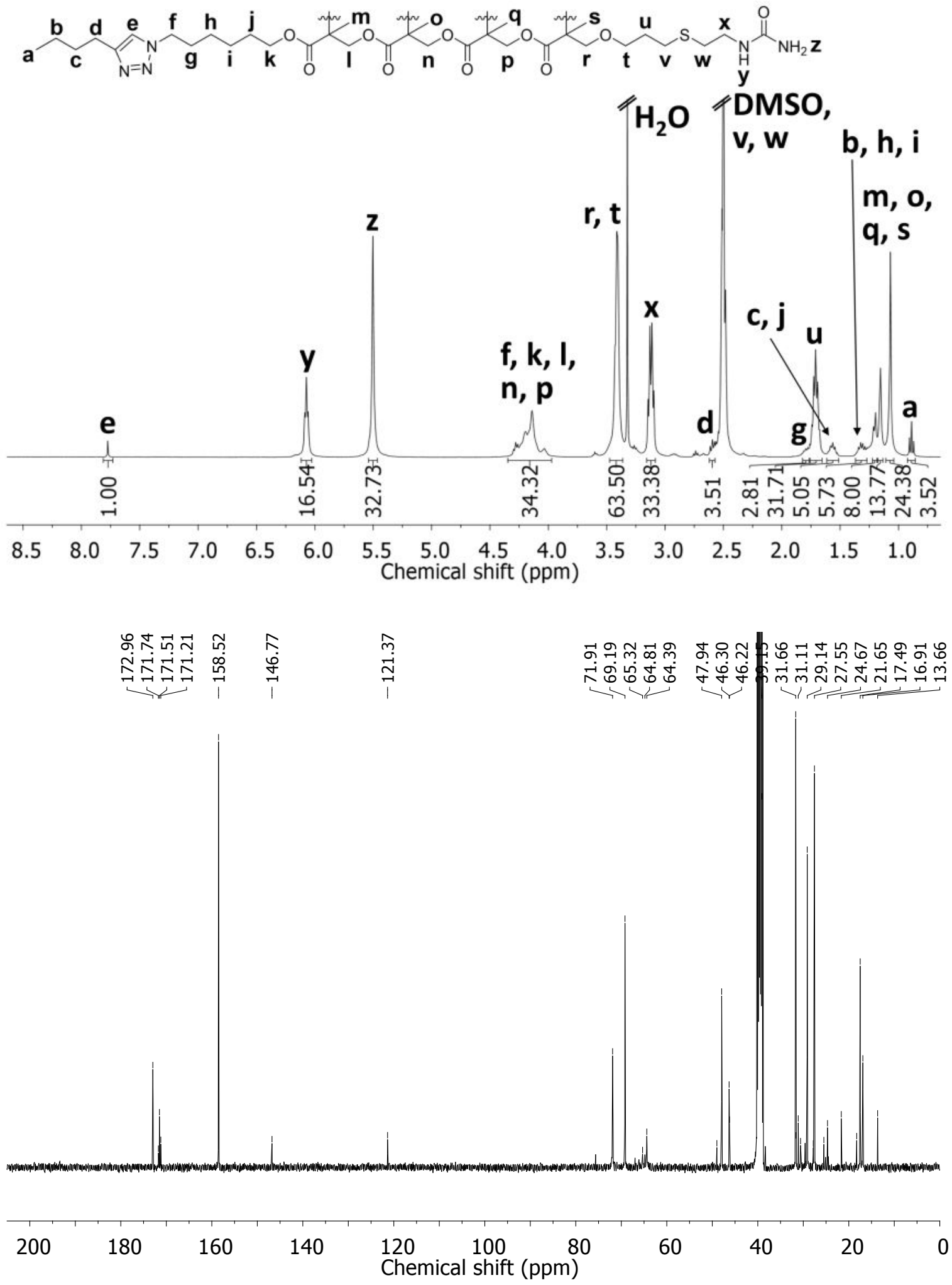

Figure S6. ^{1}H NMR (top) and ^{13}C NMR (bottom) spectra of **C$_6$-d16U**.

3.3. Synthesis and characterization of C$_6$-d16GA

A solution of **GA-SH** thiol (350.0 mg, 2.15 mmol) and **C$_6$-d16Allyl** (175.0 mg, 0.07 mmol, 1.08 mmol of alkene groups) in deoxygenated DMF (5 mL) was prepared. Then, DMPA (13.8 mg, 0.05 mmol) was added to the solution. Under argon atmosphere, the

reaction was stirred at RT inside of a chamber equipped with interior mirrors and irradiated by a Philips PL-S-9W UV Hg lamp with a maximum emission at 365 nm, for 10 h, until complete functionalization was confirmed by ^{1}H NMR. Subsequently, the mixture was precipitated in diethyl ether (20 mL). The isolated solid was dissolved in DMSO and dialyzed against DMSO (MWCO 1000 Da) for 24 h followed by an additional 24 h dialysis against Milli-Q® water. The aqueous dialyzed dispersion was freeze-dried to yield **C_6-d16GA** as a white solid. Yield: 58%. FTIR (KBr), (ν, cm^{-1}): 3392, 3315, 3199 (N–Hst), 2935, 2867 (Csp^3–Hst), 1737, 1664 (C=Ost), 1628, 1547 (N–H$^{\delta}$). ^{1}H NMR (400 MHz, DMSO-d$_6$) δ (ppm): 8.08 (t, *J*=5.9 Hz, 16H), 7.77 (s, 1H), 7.25 (s broad, 16H), 7.04 (s broad, 16H), 4.35-3.94 (m, 32H), 3.63 (d, *J*=5.8 Hz, 32H), 3.46-3.35 (m, 64H), 2.66 (t, *J*=5.8 Hz, 32 H), 2.59 (t, *J*=7.6 Hz, 2H), 2.50-2.47 (m, 32H), 2.41 (t, *J*=7.4 Hz, 32H), 1.84-1.75 (m, 2H), 1.70 (quint, *J*=6.6 Hz, 32H), 1.61-1.51 (m, 4H), 1.37-1.27 (m, 6H), 1.22-1.18 (m, 9H), 1.15 (s, 12H), 1.07 (s, 24H), 0.89 (t, *J*=7.5 Hz, 3H). ^{13}C NMR (100 MHz, DMSO-d$_6$) δ (ppm): 173.0, 171.8, 171.5, 171.2, 171.0, 170.5, 146.8, 121.4, 71.9, 69.1, 66.2, 65.3, 64.4, 49.0, 48.0, 46.3, 46.2, 41.9, 35.6, 31.1, 29.6, 29.0, 27.8, 27.7, 26.9, 25.9, 25.5, 24.7, 21.7, 18.1, 17.5, 17.0, 16.9, 13.7.

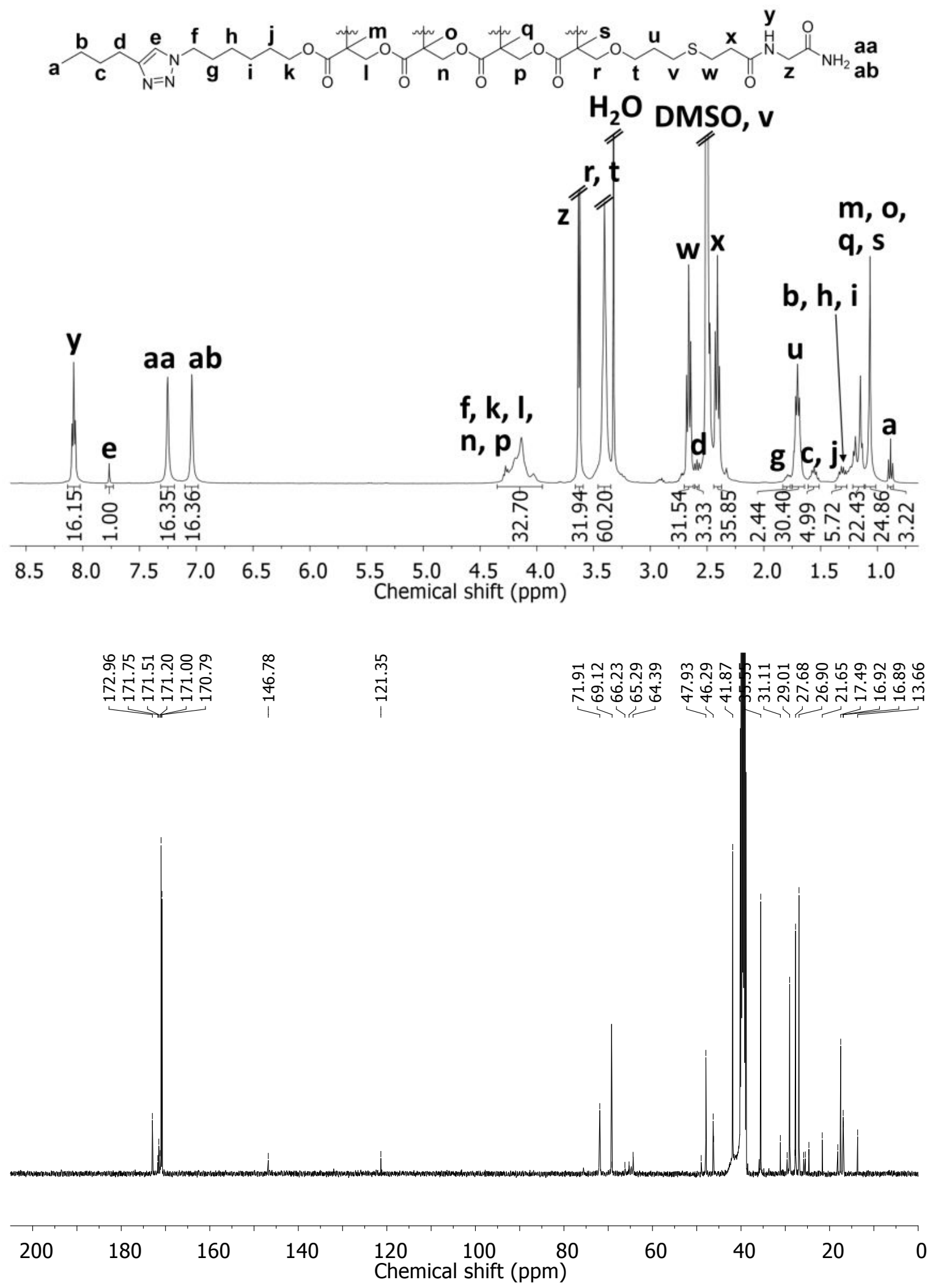

Figure S7. ^{1}H NMR (top) and ^{13}C NMR (bottom) spectra of **C_6-d16GA**.

3.4. Synthesis and characterization of PEG$_{45}$-*b*-d16U

A solution of **PEG$_{45}$-*b*-d16Allyl** (620.0 mg, 0.13 mmol, 2.14 of alkene groups) and **U-SH** thiol (516.0 mg, 4.29 mmol) in deoxygenated DMF (4 mL) was prepared. Then, DMPA (27.5 mg, 0.11 mmol) was added. Then, the reaction was magnetically stirred and carried out under argon atmosphere at RT inside of a chamber equipped with interior mirrors and illuminated by a Philips PL-S-9W UV Hg lamp with a maximum emission at 365 nm, for 3 h, until complete functionalization was confirmed by ^{1}H NMR. Afterwards, the reaction was precipitated into cold diethyl ether (20 mL). The solid was dispersed in Milli-Q®

water and dialyzed against water (MWCO 1000 Da) for 72 h, with water changes performed three times. Finally, the dialysate was freeze-dried to obtain the **PEG$_{45}$-*b*-d16U** polymer as a colorless sticky wax. Yield: 74%. FTIR (KBr), (ν, cm^{-1}): 3437, 3344, 3205 (N–Hst), 2925, 2874 (Csp^3–Hst), 1738, 1662 (C=Ost), 1608 (NH$_2^{\delta}$), 1548 (N–H$^{\delta}$). ^{1}H NMR (400 MHz, DMSO-d$_6$) δ (ppm): 7.82 (s, 1H), 6.08 (t, *J*=6.0 Hz, 16H), 5.51 (s, 32H), 4.36-3.97 (m, 34H), 3.54-3.47 (m, 178H), 3.45-3.36 (m, 64H), 3.24 (s, 3H), 3.12 (q, *J*=7.0 Hz, 32H), 2.87 (t, *J*=7.8 Hz, 2H), 2.67 (t, *J*=7.4 Hz, 2H), 2.53-2.46 (m, 64H), 1.83-1.76 (m, 2H), 1.71 (quint, *J*=6.6 Hz, 32H), 1.62-1.52 (m, 2H), 1.39-1.27 (m, 4H), 1.23-1.18 (m, 9H), 1.15 (s, 12H), 1.07 (s, 24H). MALDI-TOF MS (matrix: dithranol, *m/z*): [M+H]$^+$. Calculated for $C_{135}H_{207}N_3O_{46}$: 6543.1; found: 6489.0.

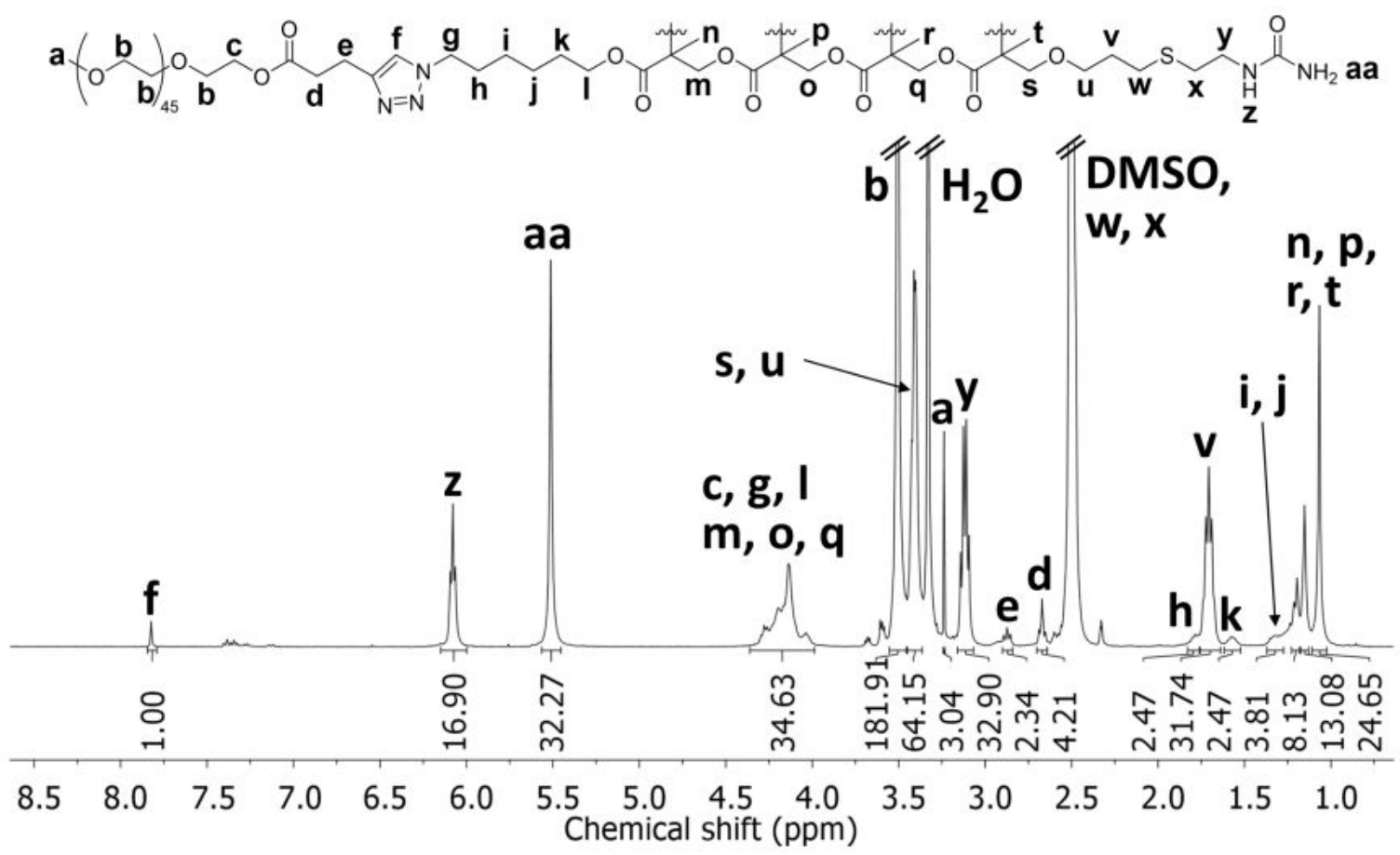

Figure S8. ^{1}H NMR spectra of **PEG$_{45}$-*b*-d16U**.

3.5. General procedure for the thiol-ene reaction in the synthesis of PEG$_m$-*b*-d16GA

DMPA (0.80 mmol) was added to a solution of **PEG$_m$-*b*-d16Allyl** (1.00 mmol, 16.00 mmol of alkene groups) and **GA-SH** thiol (32.00 mmol) in deoxygenated DMF (approx. 1 mL per 100.0 mg of **PEG$_m$-*b*-d16Allyl**) under argon atmosphere. Then, the reaction was magnetically stirred and carried out under argon atmosphere at RT inside of a chamber

equipped with interior mirrors and illuminated by a Philips PL-S-9W UV Hg lamp with a maximum emission at 365 nm, for 6 h, until complete functionalization was confirmed by ^{1}H NMR. The isolated crude was dissolved in DMSO and dialyzed against DMSO (MWCO 1000 Da) for 24 h followed by an additional 24 h dialysis against Milli-Q® water. The aqueous dialyzed dispersion was freeze-dried to isolate the final **PEG$_m$-*b*-d16GA** as white solids in all cases.

*Characterization of **PEG$_{45}$-b-d16GA**.* **PEG$_{45}$-*b*-d16GA** was synthesized according to the general procedure for thiol-ene reaction, employing the following reactants and solvents: **PEG$_{45}$-*b*-d16Allyl** (200.0 mg, 0.04 mmol), **GA-SH** thiol (224.2 mg, 1.38 mmol), DMPA (8.9 mg, 0.03 mmol) and DMF (2 mL). Yield: 44%. FTIR (KBr), (ν, cm^{-1}): 3390, 3316, 3199 (N–Hst), 2921, 2874 (Csp^3–Hst), 1739, 1663, 1644 (C=Ost), 1626, 1548 (N–H$^{\delta}$). ^{1}H NMR (400 MHz, DMSO-d$_6$) δ (ppm): 8.08 (t, *J*=5.8 Hz, 16H), 7.81 (s, 1H), 7.25 (s broad, 16H), 7.04 (s broad, 16H), 4.33-3.98 (m, 34H), 3.65-3.60 (m, 34H), 3.53-3.49 (m, 176H), 3.44-3.37 (m, 64H), 3.24 (s, 3H), 2.87 (t, *J*=7.5 Hz, 2H), 2.66 (t, *J*=7.4 Hz, 34H), 2.52-2.46 (m, 32H), 2.41 (t, *J*=7.3 Hz, 32H), 1.83-1.76 (m, 2H), 1.70 (quint, *J*=6.5 Hz, 32H), 1.61-1.53 (m, 2H), 1.37-1.28 (m, 4H), 1.22-1.18 (m, 9H), 1.15 (s, 12H), 1.07 (s, 24H).

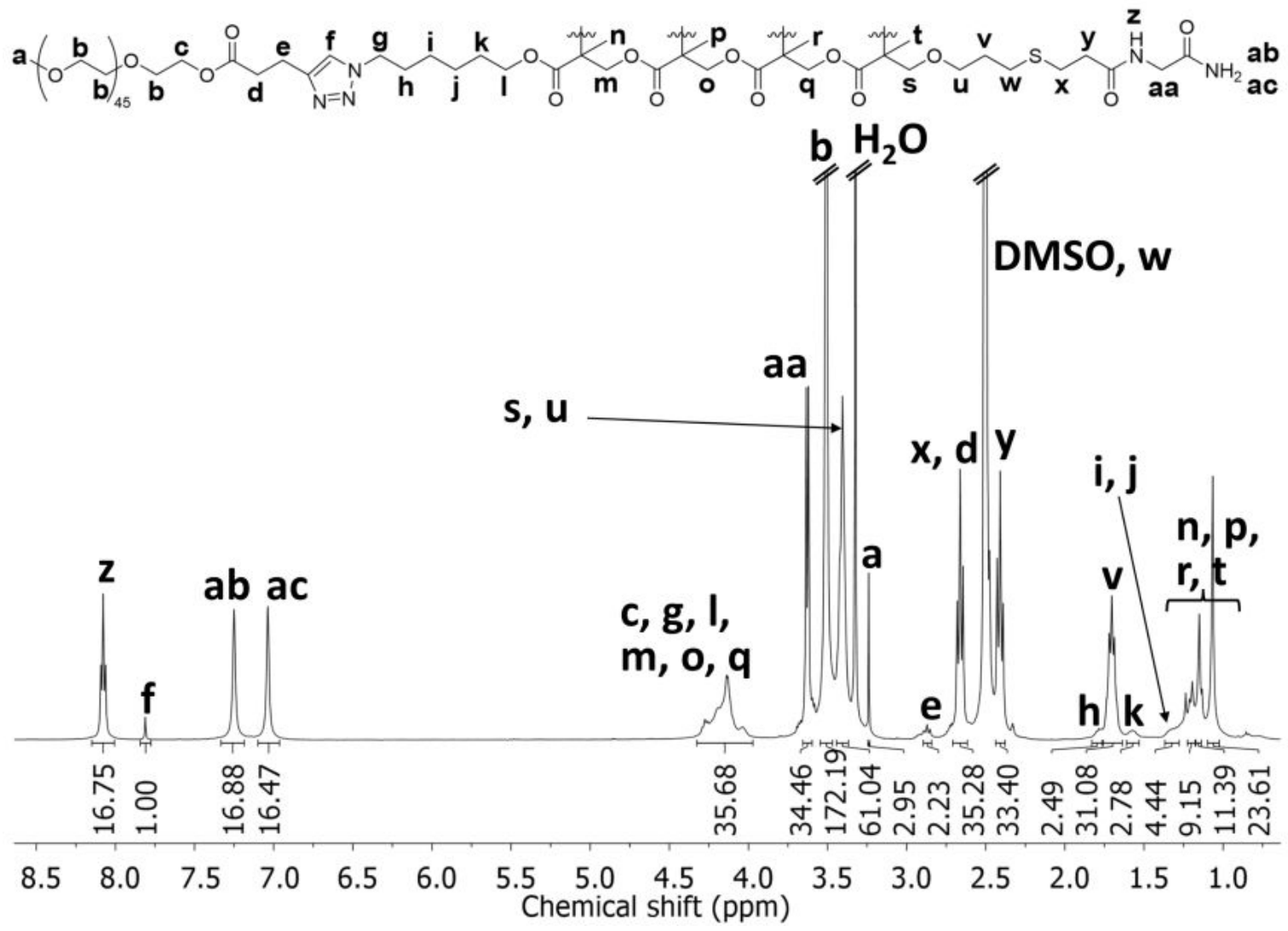

Figure S9. ^{1}H NMR spectra of **PEG$_{45}$-*b*-d16GA**.

*Characterization of **PEG$_{113}$-b-d16GA**.* **PEG$_{113}$-*b*-d16GA** was synthesized according to the general procedure for thiol-ene reaction, employing the following reactants and solvents: **PEG$_{113}$-*b*-d16Allyl** (171.0 mg, 0.02 mmol), **GA-SH** thiol (116.7 mg, 0.72 mmol), DMPA (4.6 mg, 0.02 mmol) and DMF (2 mL). Yield: 48%. FTIR (KBr), (ν, cm^{-1}): 3396, 3320, 3201 (N–Hst), 2914, 2875 (Csp^{3}–Hst), 1738, 1663, 1643 (C=Ost), 1626, 1549 (N–H$^{\delta}$). ^{1}H NMR (400 MHz, DMSO-d$_6$) δ (ppm): 8.07 (t, *J*=5.6 Hz, 16H), 7.81 (s, 1H), 7.25 (s broad, 16H), 7.03 (s broad, 16H), 4.37-3.97 (m, 34H), 3.66-3.60 (m, 34H), 3.55-3.46 (m, 448H), 3.44-3.36 (m, 64H), 3.24 (s, 3H), 2.87 (t, *J*=7.7 Hz, 2H), 2.66 (t, *J*=7.7 Hz, 34H), 2.52-2.46 (m, 32H), 2.41 (t, *J*=7.5 Hz, 32H), 1.84-1.62 (m, 34H), 1.61-1.52 (m, 2H), 1.38-1.24 (m, 4H), 1.23-1.18 (m, 9H), 1.17-1.12 (m, 12H), 1.11-1.02 (m, 24H).

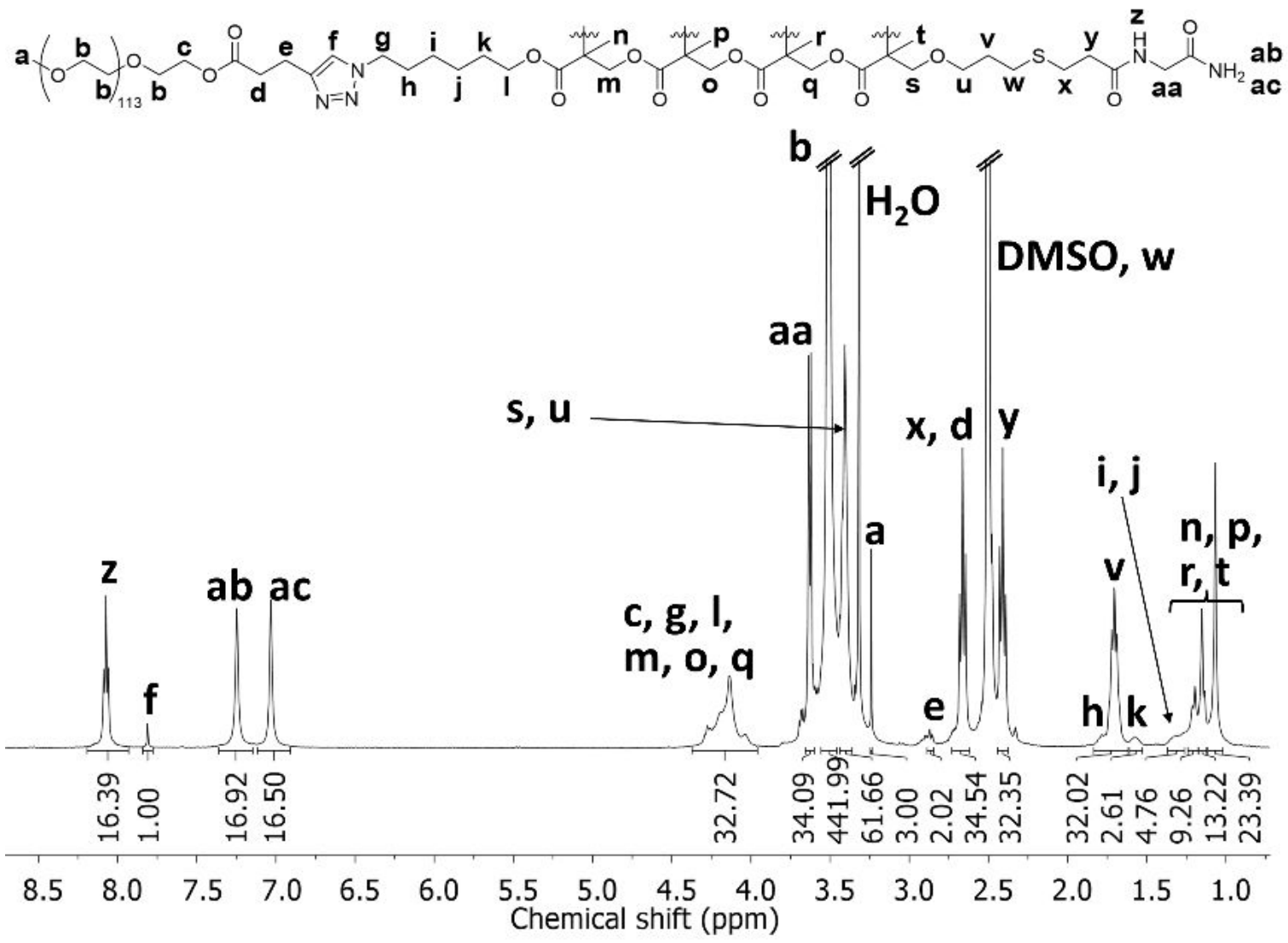

Figure S10. ^{1}H NMR spectra of **PEG_{113}-*b*-d16GA**.

*Characterization of **PEG_{226}-b-d16GA**.* **PEG_{226}-*b*-d16GA** was synthesized according to the general procedure for thiol-ene reaction, employing the following reactants and solvents: **PEG_{226}-*b*-d16Allyl** (200.0 mg, 0.02 mmol), **GA-SH** thiol (82.5 mg, 0.51 mmol), DMPA (3.3 mg, 0.01 mmol) and DMF (2 mL). Yield: 51%. FTIR (KBr), (ν, cm^{-1}): 3392, 3316, 3206 (N–Hst), 2943, 2890 (Csp^{3}–Hst), 1738, 1662, 1643 (C=Ost), 1628, 1551 (N–H$^{\delta}$). ^{1}H NMR (400 MHz, DMSO-d_6) δ (ppm): 8.08 (t, *J*=5.6 Hz, 16H), 7.81 (s, 1H), 7.25 (s broad, 16H), 7.04 (s broad, 16H), 4.34-3.96 (m, 34H), 3.65-3.60 (m, 34H), 3.57-3.45 (m, 900H), 3.44-3.36 (m, 64H), 3.24 (s, 3H), 2.90-2.84 (m, 2H), 2.71-2.61 (m, 34H), 2.52-2.46 (m, 32H), 2.41 (t, *J*=7.2 Hz, 32H), 1.82-1.63 (m, 34H), 1.61-1.53 (m, 2H), 1.38-1.27 (m, 4H), 1.24-1.12 (m, 21H), 1.10-1.01 (m, 24H).

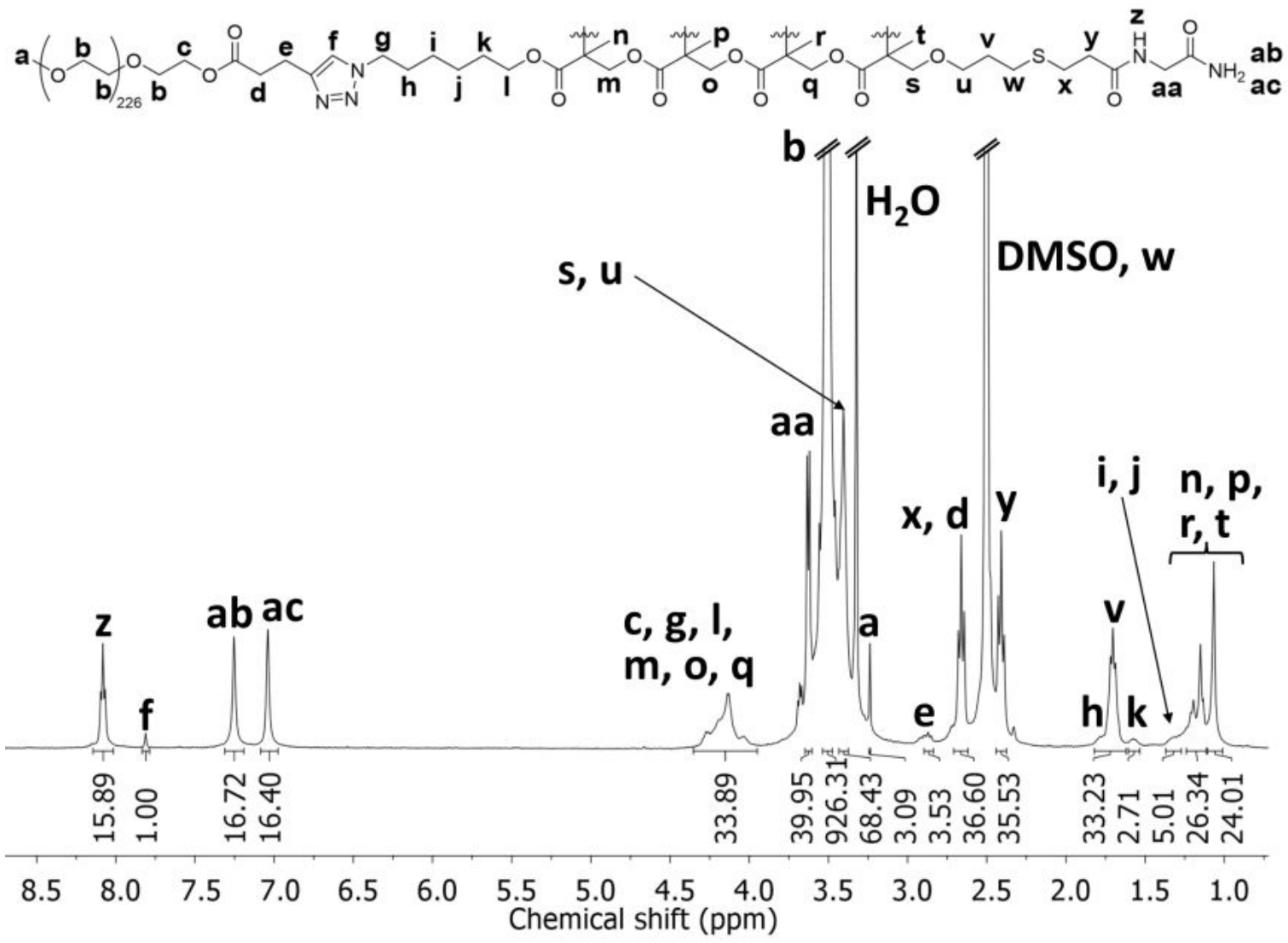

Figure S11. ^{1}H NMR spectra of **PEG$_{226}$-*b*-d16GA**.

4. Self-assembly of amphiphilic LDBCs by the co-solvent method

A solution of the LDBC (5.0 mg) in DMSO (1 mL) was prepared, and Milli-Q® water was gradually added while monitoring the absorbance of the solution at 650 nm using a UV-Vis spectrometer. Changes in absorbance served as an indirect determination of the loss of transmitted light resulting from the scattering of incident light due to the formation of self-assemblies. The addition of Milli-Q® water was concluded when absorbance values remained unchanged. Subsequently, the mixture was dialyzed against Milli-Q® water using a Spectra Por® dialysis membrane (MWCO 1000 Da) for 3 days, with the water changed three times during the dialysis process.

5. Preparation of samples for XRD measurements

Samples for XRD analysis were prepared as follows. First, the self-assemblies were prepared by the co-solvent method at a final polymer concentration of 20.0 mg mL^{-1}. The resulting aqueous dispersion was then deposited onto a 1 cm × 1 cm glass substrate and dried under a steady air flow, followed by vacuum drying at RT.

6. Determination of the Critical Aggregation Concentration (CAC)

CAC was determined by fluorescence spectroscopy using Nile Red as polarity sensitive probe. A solution of Nile Red in DCM at concentration of 6.0×10^{-6} M was prepared. Then, 100 μL of this solution was added to a vial, and the solvent evaporated at RT for 6 h Subsequently, 600 μL of the self-assembly dispersion, with concentrations ranging from 1.0×10^{-4} to 1.0 mg mL^{-1}, were added into the vial, achieving a final Nile Red concentration of 1.0×10^{-6} M. The mixture was then stirred overnight in orbital shaker. Finally, the emissions spectra of Nile Red solution were registered from 530 to 900 nm while exciting at 550 nm.

7. TEM images

For the preparation of the samples at RT, 10 μL of the LDBC suspension was deposited onto carbon-coated copper grid. After 30 s, the excess of water was removed by capillarity using a paper filter. Then, sample was stained adding 10 μL of uranyl acetate (1 wt %) solution in water onto the sample grid. After 30 s, the excess of staining agent was removed by capillarity using a paper filter. Finally, the grid was dried under vacuum overnight. For the preparation of the samples at 70 °C, the carbon coated copper grid, the LDBC suspension and the staining agent, phosphotungstic acid (3 wt %) solution in water, were heated in oven at 70 °C for 30 min. Then, the LDBC dispersion was deposited onto grid at 70 °C. The excess of water was removed by capillarity. Subsequently, phosphotungstic acid solution was deposited onto the sample grid at 70 °C. The excess of stain was removed by capillarity. The TEM diameter was determined as the mean of 150 measurements histogram.

8. Variable temperature ^{1}H NMR experiments

LDBCs were dispersed in D_2O at a 5.0 mg mL^{-1} polymer concentration. The variable temperature ^{1}H NMR experiments of LDBCs used a coaxial insert for NMR tube. The outer NMR tube contained LDBCs dispersion in D_2O at 5.0 mg mL^{-1}. The inner tube contained a pyridine solution in $C_2D_2Cl_4$ as the external standard. Spectra were acquired from 20 °C to 80 °C at 5 °C intervals with 5 min equilibration.

9. Variable temperature SAXS experiments

For experiments performed upon heating and cooling ramps, a capillary containing the sample was inserted into a Linkam THM600 hot stage. SAXS patterns were acquired while the sample was heated from 20 ˚C to 80 ˚C at a controlled rate of 1.0 ˚C min^{-1} and the measurements were taken every 5 ˚C. The angular (q axis) was calibrated using standard samples of Silver Behenate. The scattering from water and that of the containing capillary was subtracted from the total scattering contribution.

10. Cell culture

Three different cell lines were used to evaluate the effect of the polymers on cell viability: J774A.1 mouse monocyte-macrophages ATCC-TIB-67™ were obtained from LGC Standards (Barcelona, Spain), human dermal fibroblasts were obtained from Lonza (Basel, Switzerland), and murine chondrogenic precursors (ATDC5). Fibroblasts and macrophages were cultured in high-glucose DMEM with stable glutamine, supplemented with 10% (v/v) FBS and 1% (v/v) PSA. ATDC5 cell was cultured in high-glucose DMEM with stable glutamine, supplemented with 5% (v/v) FBS, 1% (v/v) PSA, 3×10^{-8} M

sodium selenite, and 10 μg mL^{-1} transferrin. All three cell lines were maintained at 37°C in a humidified atmosphere with 5% CO_2.

11. Cell viability

The cytotoxicity assay for the different compounds was conducted using the Blue Cell Viability Assay following the manufacturer's instructions. Briefly, 6,000 cells per well were seeded and incubated for 24 h at 37 ˚C in a 5% CO_2 atmosphere. Then, the compounds were added at different concentrations and, after 24 h of incubation, the alamarBlue Cell Viability reagent was added at 10% (v/v). After 4 h at 37 ˚C and 5% CO_2, fluorescence was measured at 530/590 ex/em in a Varioskan LUX microplate reader (Thermo Fisher, USA). Untreated cells were considered as control samples. Cellular cytotoxicity was assessed by comparing the measurements from treated cells to those obtained from untreated controls, which were considered to have 100% viability. Each experiment was conducted with at least three independent replicates.

12. Cell cycle

Flow cytometry was used to study the effect of the polymer on the cell cycle of J774A.1 mouse monocyte-macrophages, identifying the phase distribution of cells after exposure to the polymer at concentrations of 2.000 and 0.125 mg mL^{-1} for 24 hours. Following exposure, cells were harvested in PBS and fixed in 70% (v/v) cold ethanol (with 2.5 µL of 1× trypsin). Samples were centrifuged for 5 minutes, and the pellet was resuspended in 500 µL of PI/RNASE Solution kit. After incubation for 15 minutes, samples were analyzed using a FACSARRAY BD system and MODIFIT 3.0 software (Verity). A control group of cells not exposed to the polymer was included.

13. Variable temperature fluorescence measurements of Nile Red loaded micelles

Sample preparation at polymer concentration of 1.0 mg mL^{-1} was carried out as described in section 5 "Determination of the Critical Aggregation Concentration (CAC)". The dispersions were heating and then cooling between 20 ˚C and 80 ˚C with a rate of 1.0 ˚C min^{-1}. Emission spectra of Nile Red were registered at every 5 ˚C. The measurements were started in the heating cycle.

14. Supplementary figures and tables

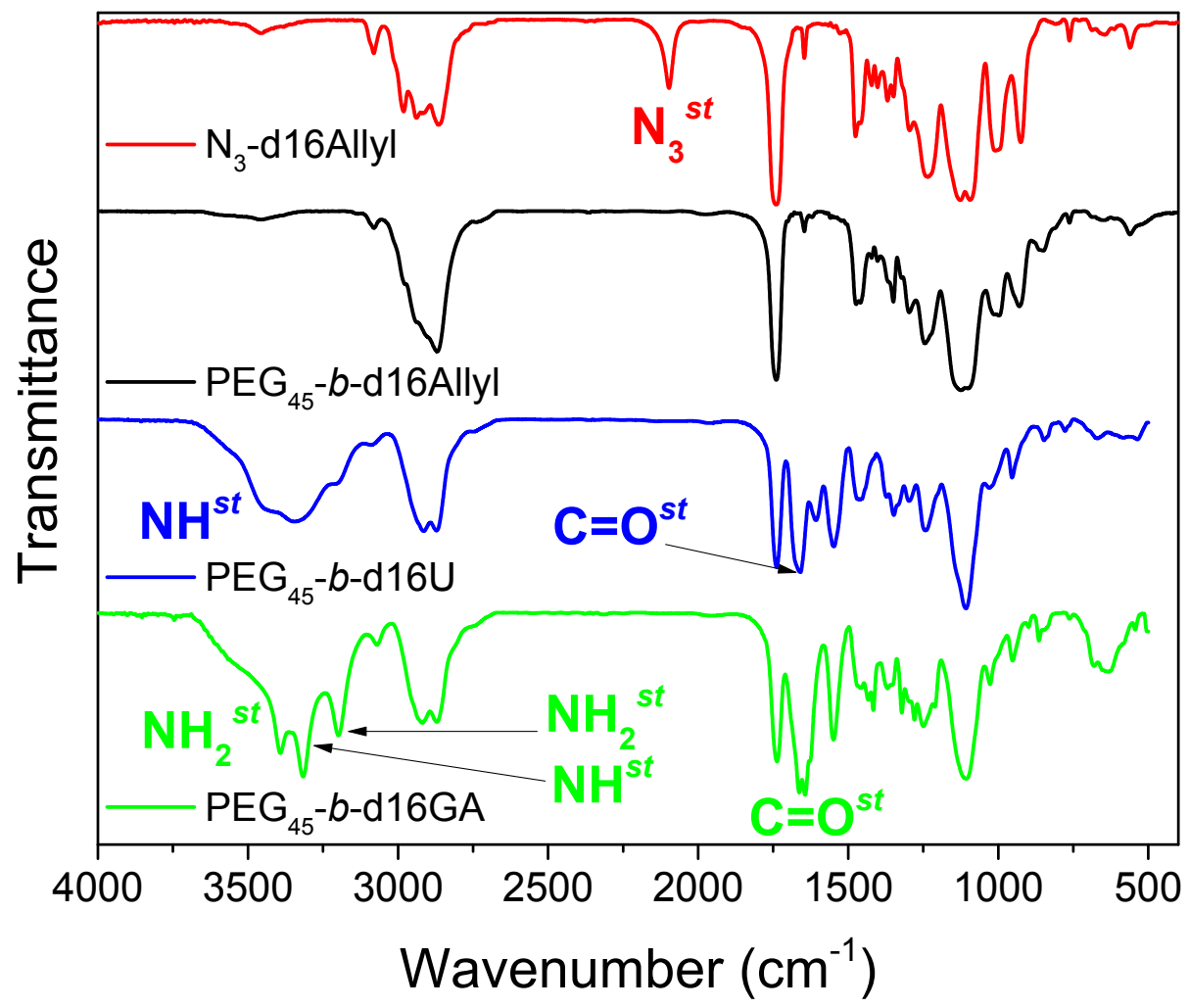

Figure S12. FTIR spectra of **N_3-d16Allyl**, **PEG_{45}-*b*-d16Allyl**, **PEG_{45}-*b*-d16U** and **PEG_{45}-*b*-d16GA**.

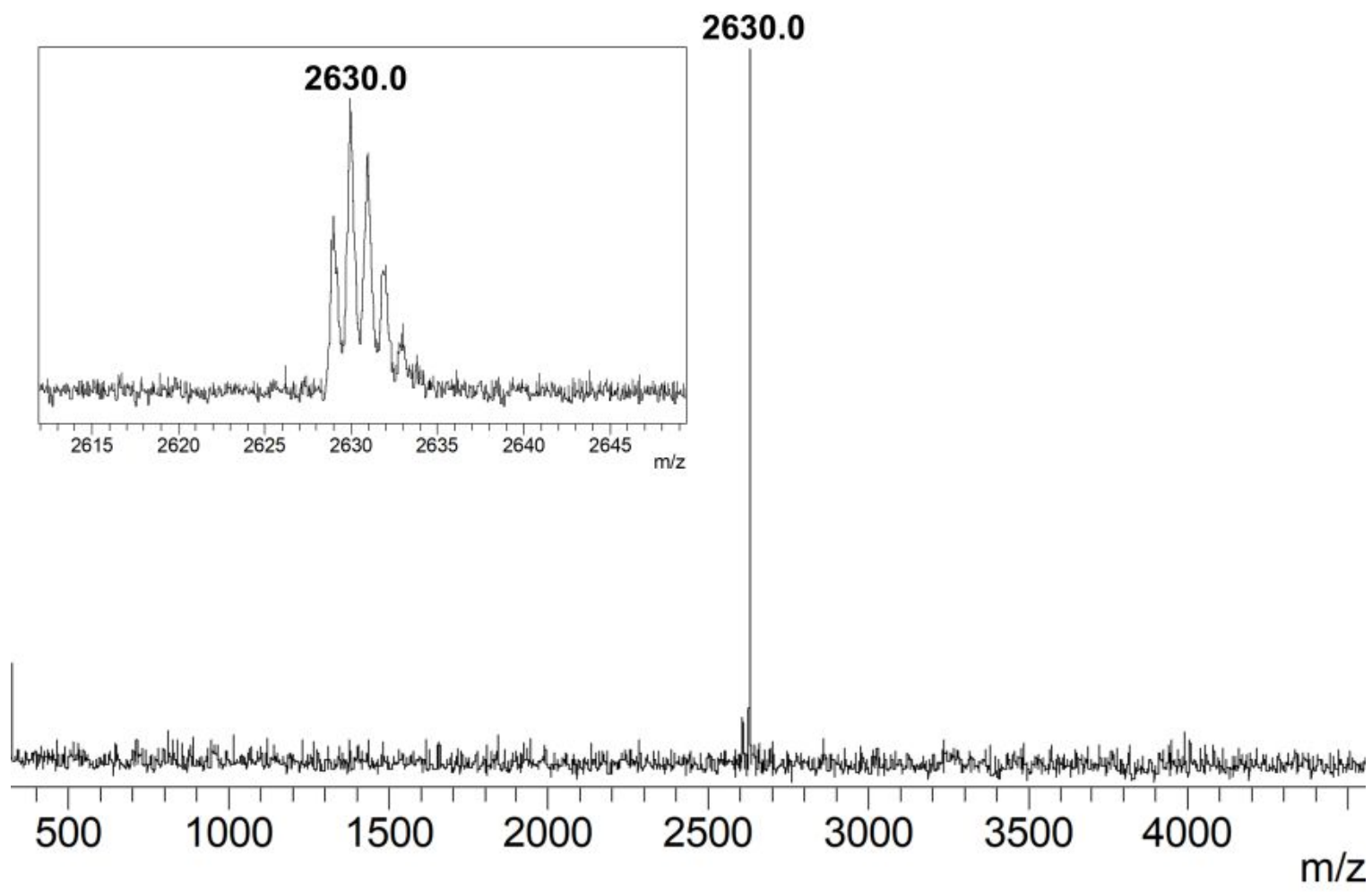

Figure S13. MALDI-TOF MS spectrum of **C_6-d16Allyl**.

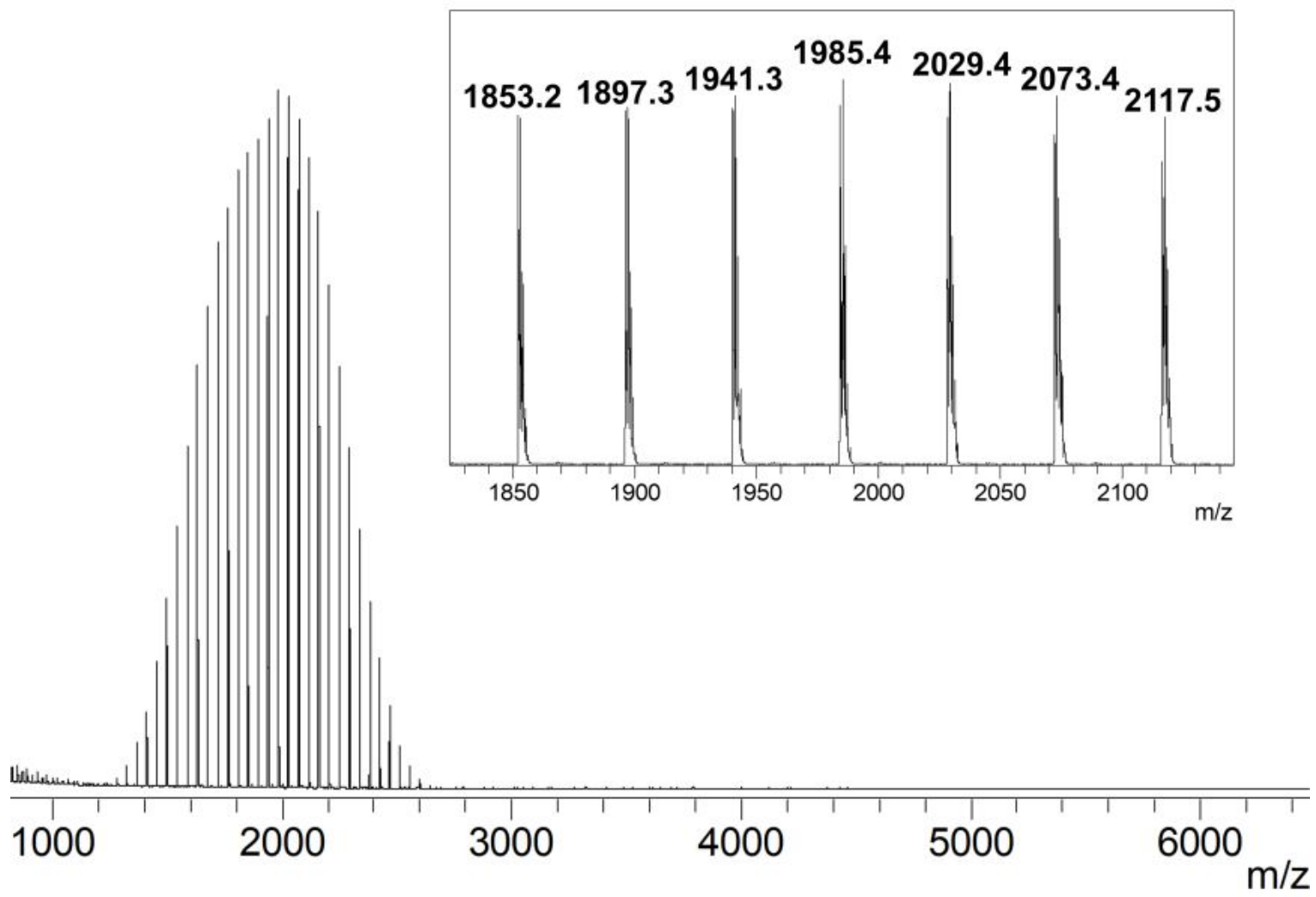

Figure S14. MALDI-TOF MS spectrum of **PEG_{45}-Alkyne**.

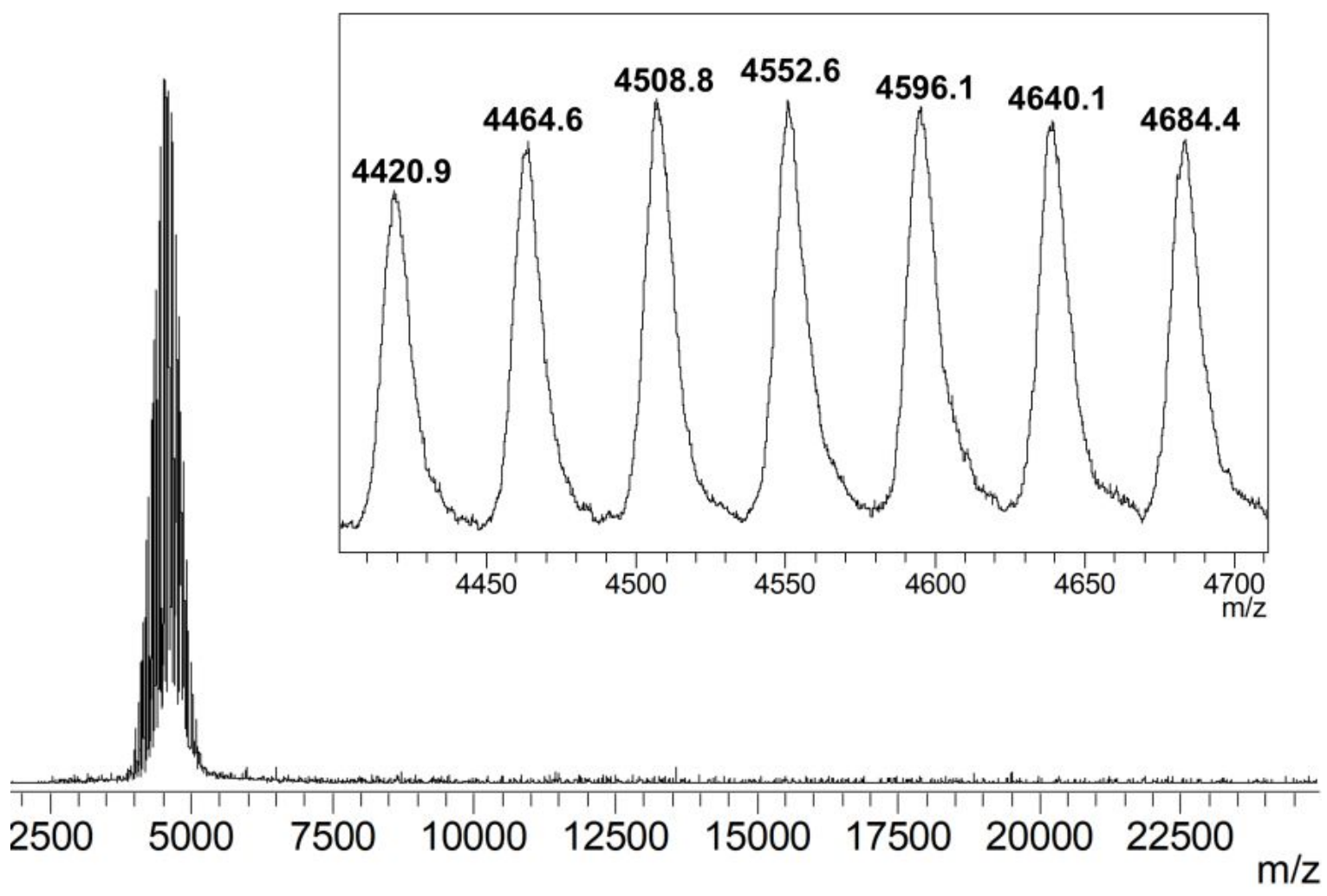

Figure S15. MALDI-TOF MS spectrum of **PEG$_{45}$-*b*-d16Allyl**.

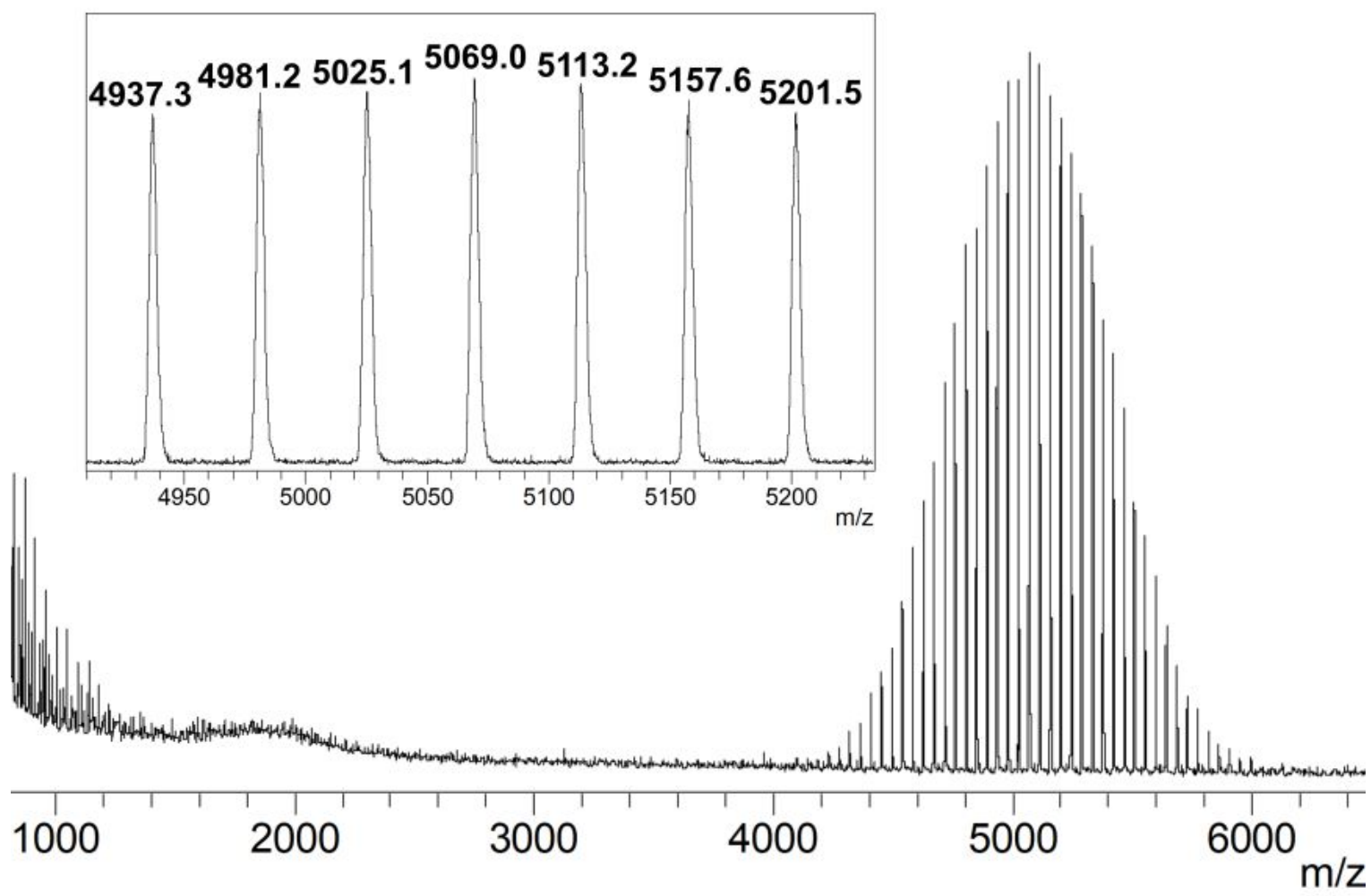

Figure S16. MALDI-TOF MS spectrum of **PEG$_{113}$-Alkyne**.

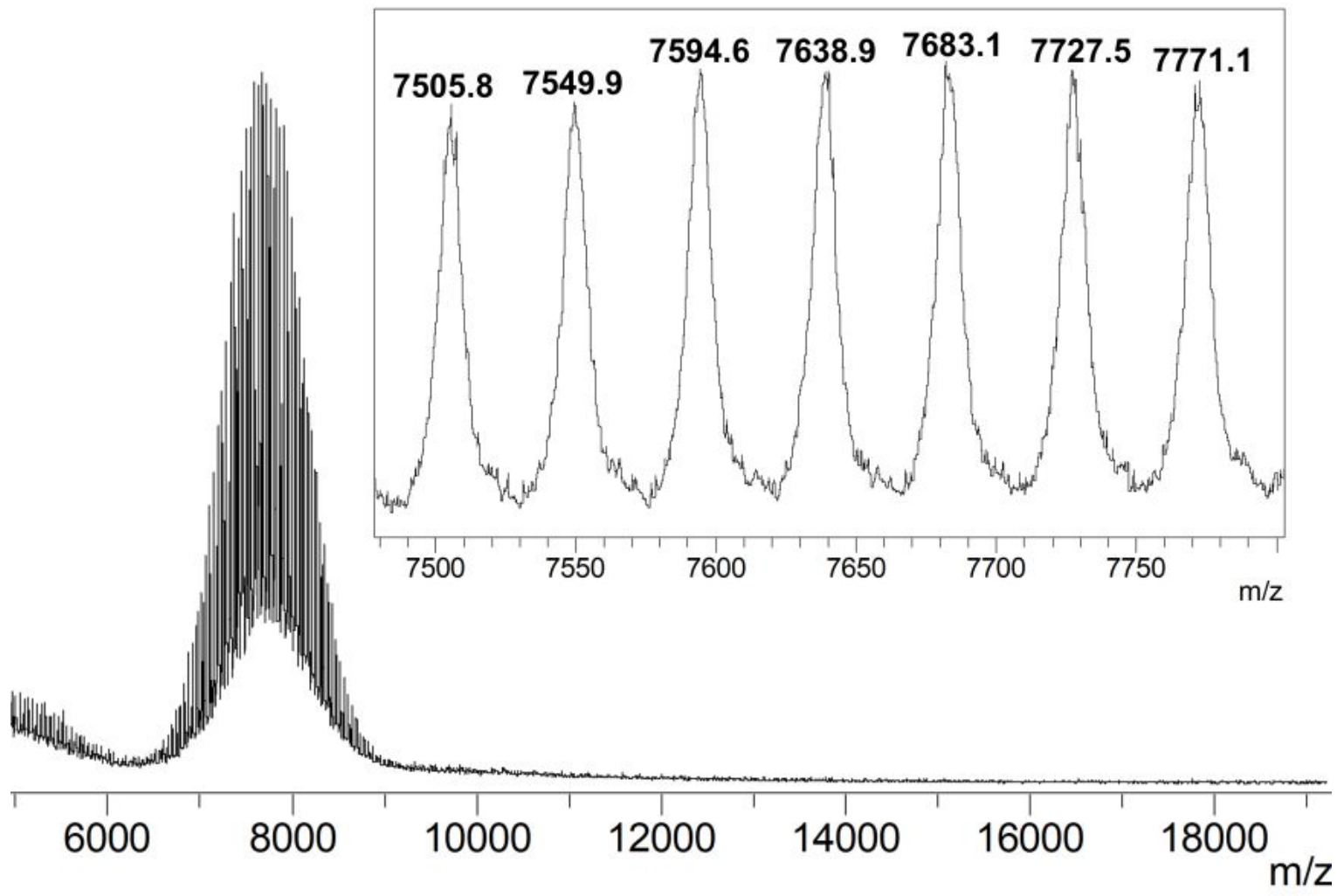

Figure S17. MALDI-TOF MS spectrum of **PEG$_{113}$-*b*-d16Allyl**.

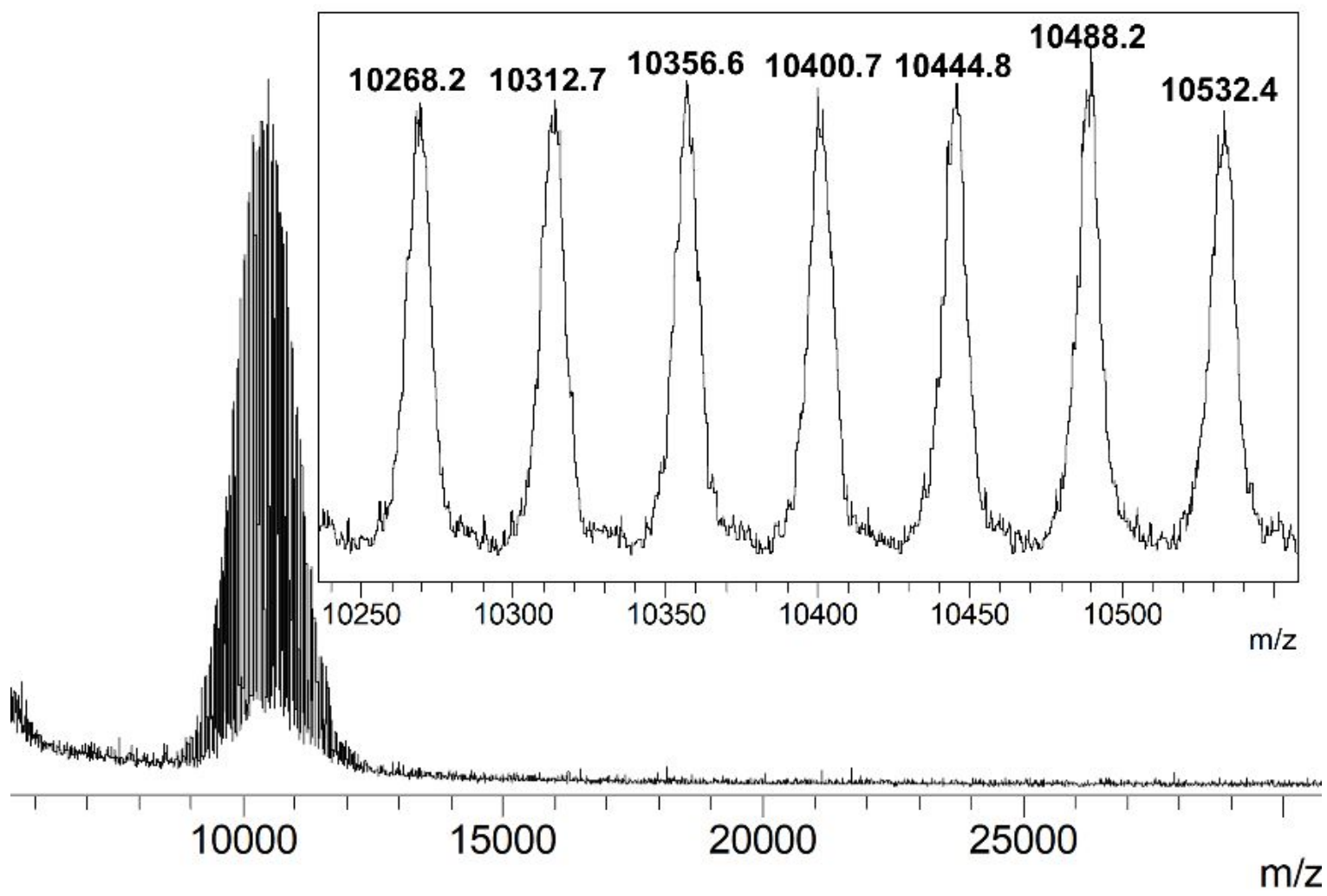

Figure S18. MALDI-TOF MS spectrum of **PEG$_{226}$-Alkyne**.

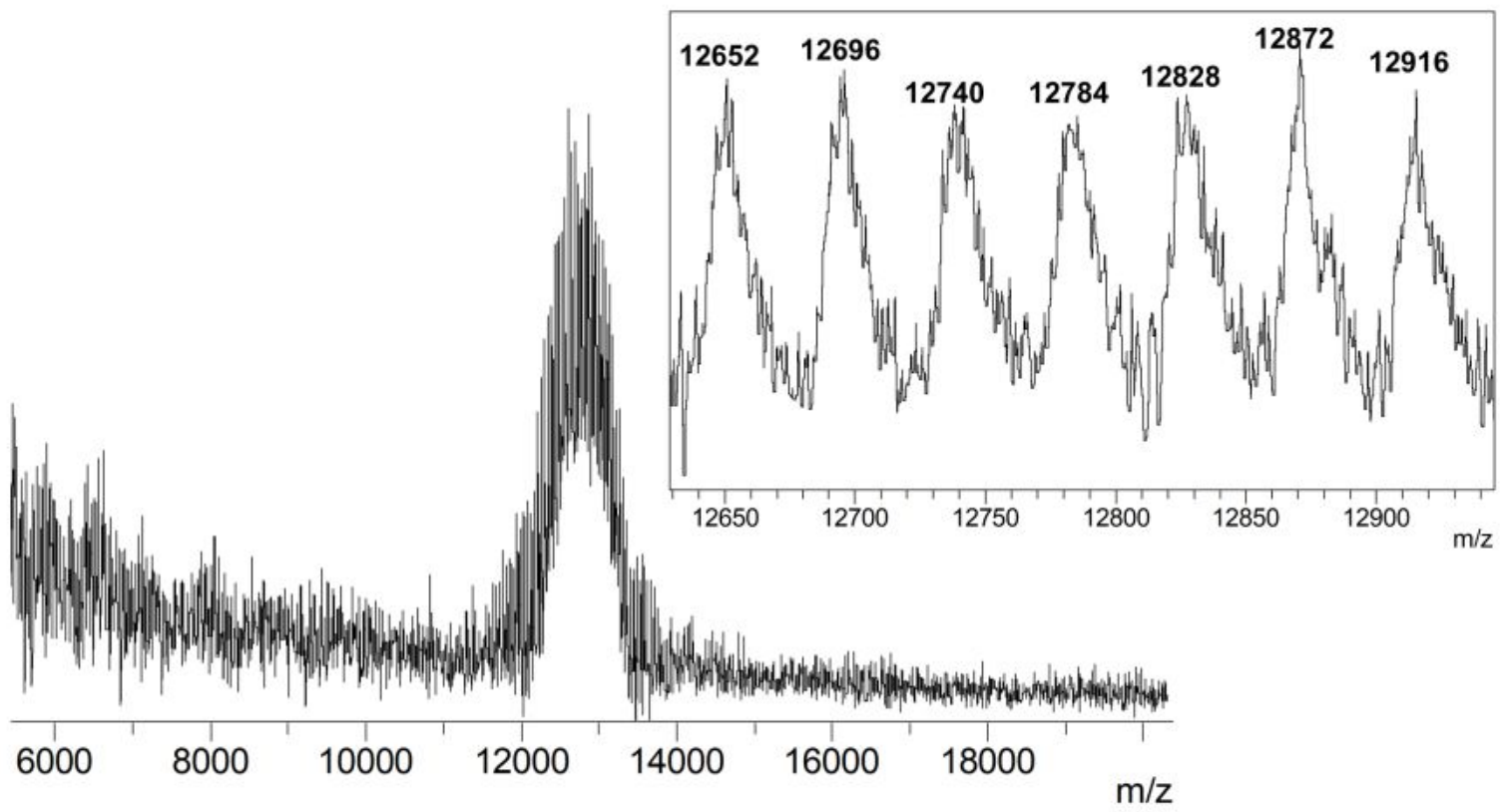

Figure S19. MALDI-TOF MS spectrum of **PEG_{226}-*b*-d16Allyl**.

Table S1. Molar masses and dispersities of alkyne-terminated **PEG$_m$-Alkyne** precursors and **PEG$_m$-*b*-d16Allyl** LDBCs.

Polymer	$M^{theoretical}$ (g mol^{-1})	M_n^{SEC} (g mol^{-1})[b]	*Đ*[b]
**PEG$_{45}$-Alkyne**	2094	3641	1.04
**PEG$_{113}$-Alkyne**	5090	9832	1.07
**PEG$_{226}$-Alkyne**	10068	20894	1.07
**PEG$_{45}$-*b*-d16Allyl**	4620[a]	6424	1.02
**PEG$_{113}$-*b*-d16Allyl**	7616[a]	14763	1.04
**PEG$_{226}$-*b*-d16Allyl**	12586[a]	25584	1.04

(a) Theoretical molar mass ($M^{theoretical}$) was calculated as sum of the molar mass of PEG block and molar mass of the dendritic block. (b) Number average molar mass (M_n) and dispersity (*Đ*) determined by SEC in THF (1 mL min^{-1}) using PMMA calibration standards and ELSD.

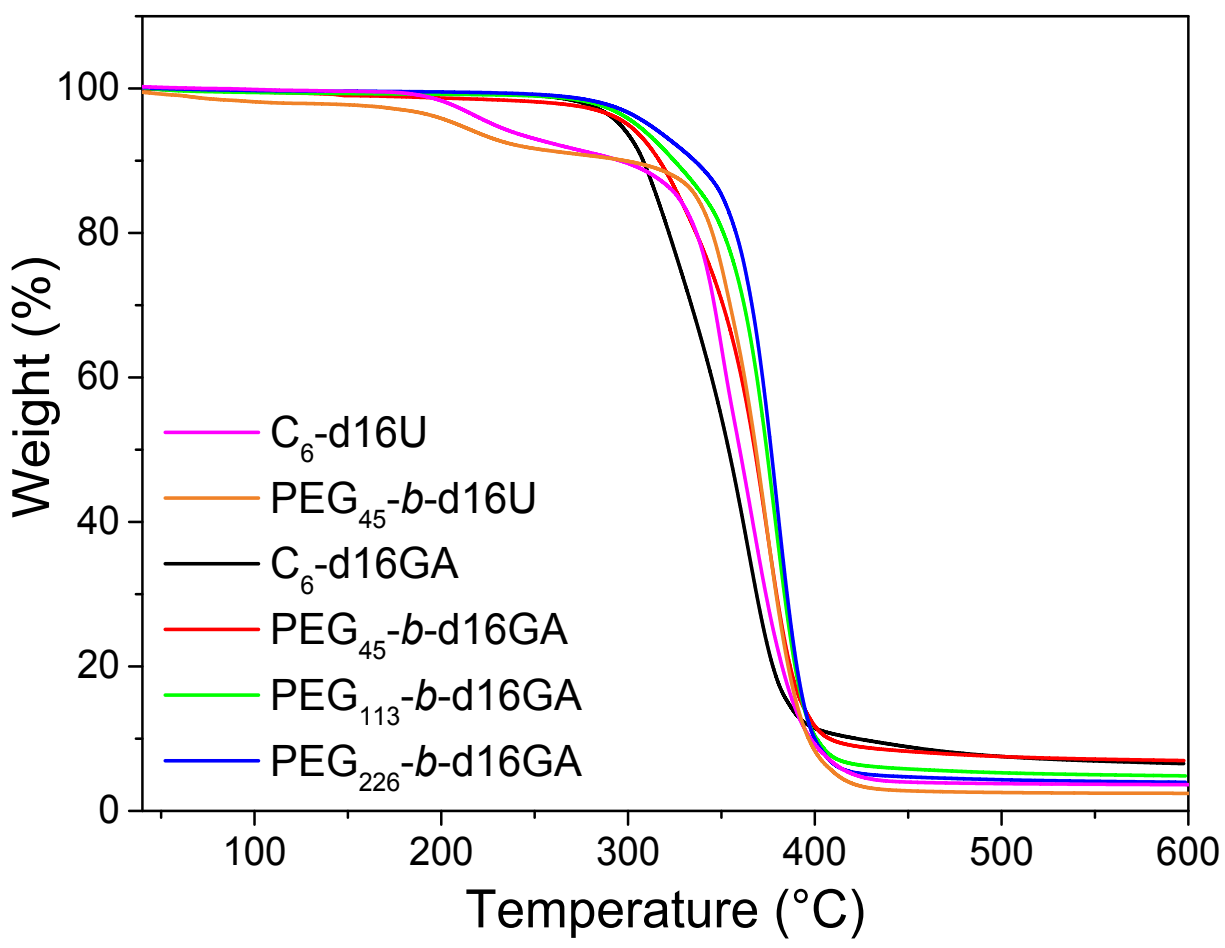

Figure S20. TGA curves of polymers registered at 10 °C min^{-1} heating rate under nitrogen atmosphere.

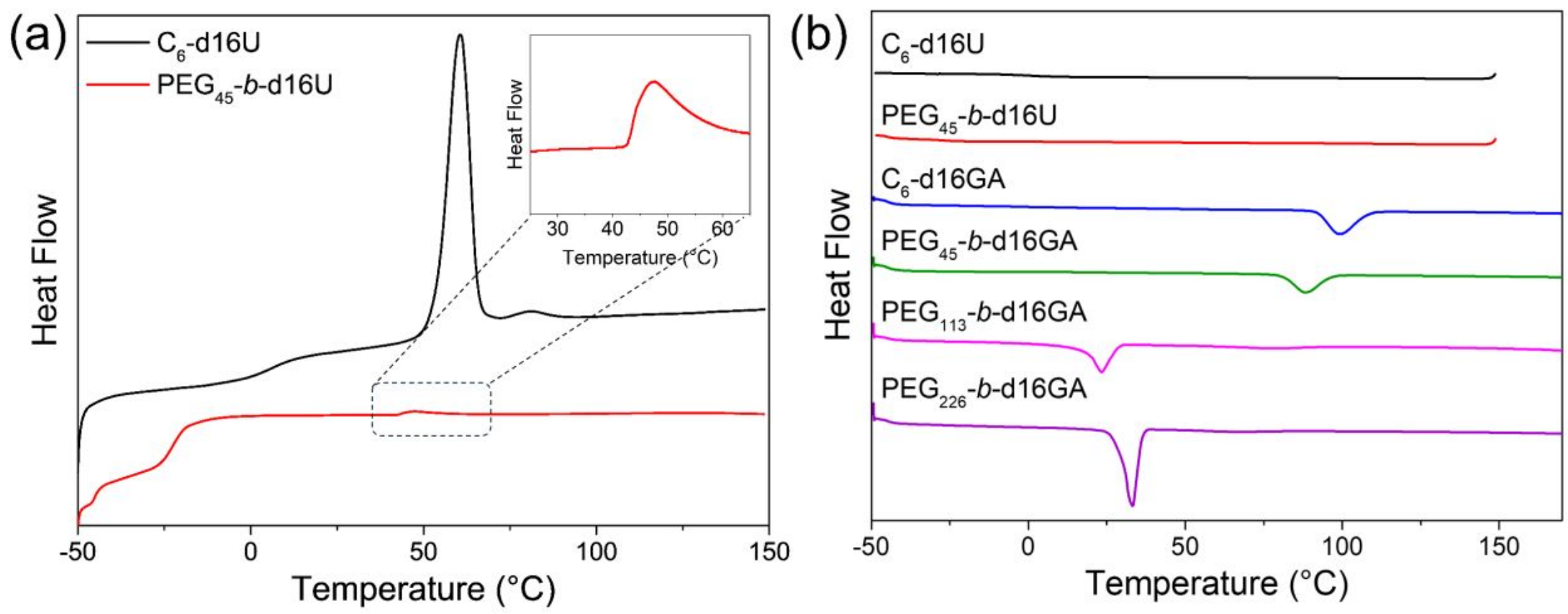

Figure S21. DSC curves corresponding to (a) first heating scan of **C_6-d16U** and **PEG_{45}-*b*-d16U** and (b) first cooling scan of dendrons and LDBCs registered at a 10 °C min^{-1} rate (exo down).

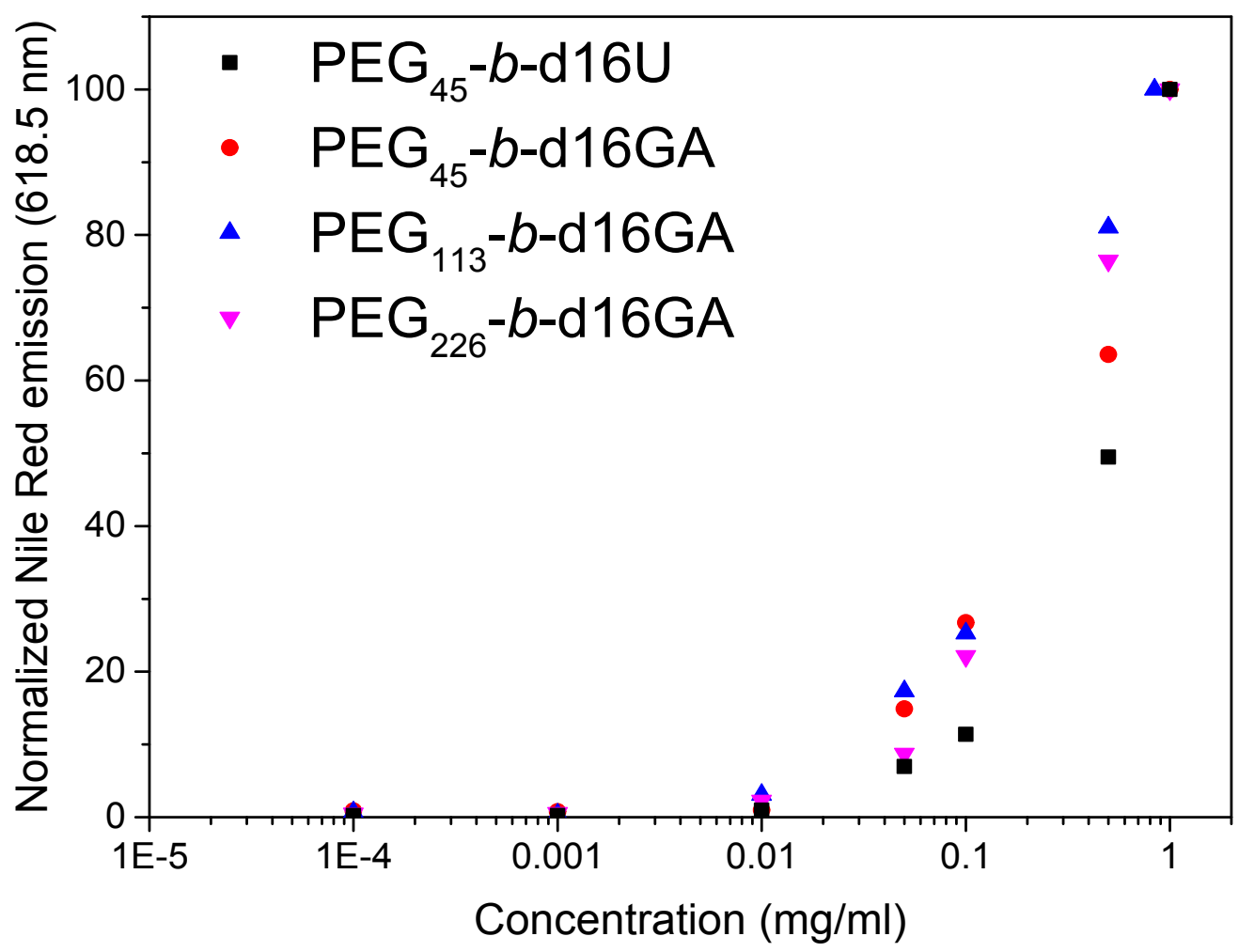

Figure S22. Normalized fluorescence emission of Nile Red at 618.5 nm (λ_{exc} = 550 nm) vs amphiphilic LDBC concentration. The CAC values were 53 µg mL^{-1} for **PEG_{45}-*b*-d16U**, 35 µg mL^{-1} for **PEG_{45}-*b*-d16GA**, 38 µg mL^{-1} for **PEG_{113}-*b*-d16GA** and 46 µg mL^{-1} for **PEG_{226}-*b*-d16GA**.

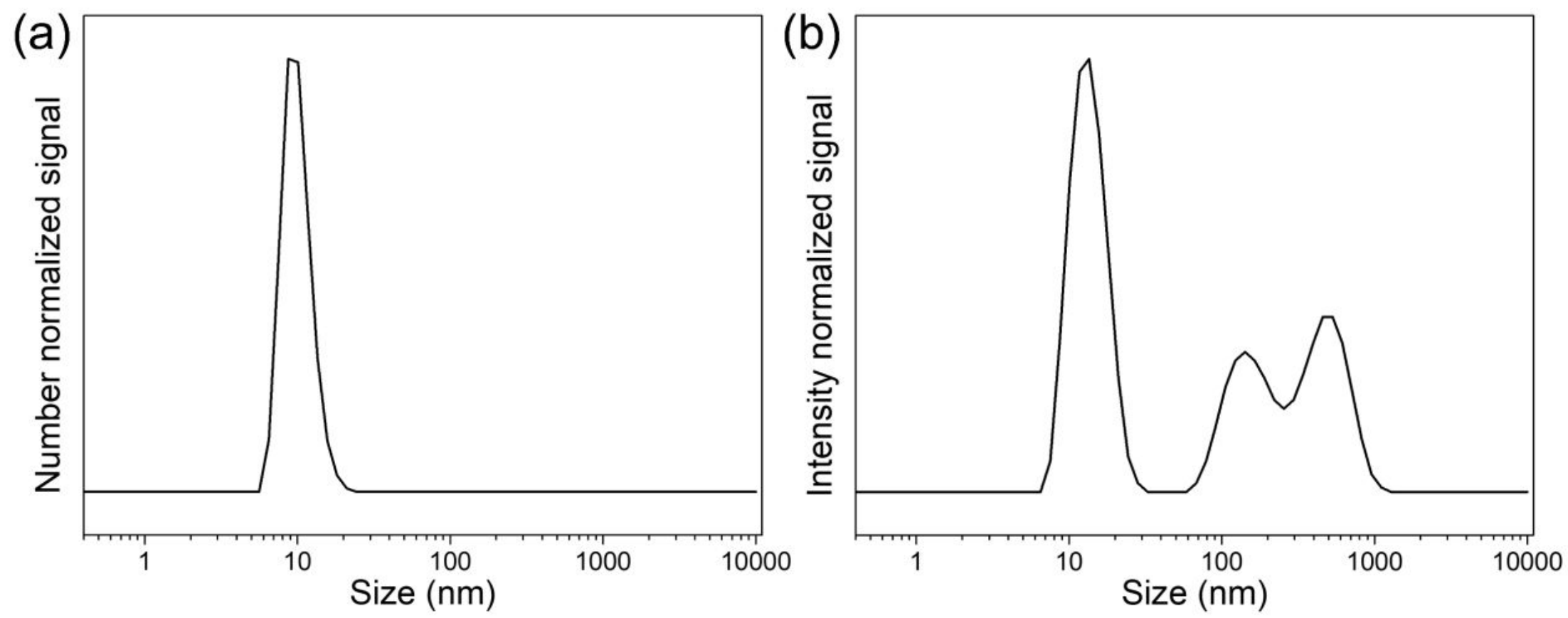

Figure S23. (a) DLS number size distribution and (b) DLS intensity size distribution at 25 ˚C of **PEG$_{45}$-*b*-d16U** self-assemblies in water.

SAXS EXPERIMENTS

Guinier Approximation for estimation of the Radius of Gyration

The Guinier approximation describes the low-q region of SAXS data, where scattering intensity decreases exponentially with q^2, allowing estimation of the radius of gyration (R_g) from the slope of the log(I) vs q^2 representation. It assumes particles are much smaller than the probed length scale, so the structure appears as a single entity without internal detail (Figure S16).

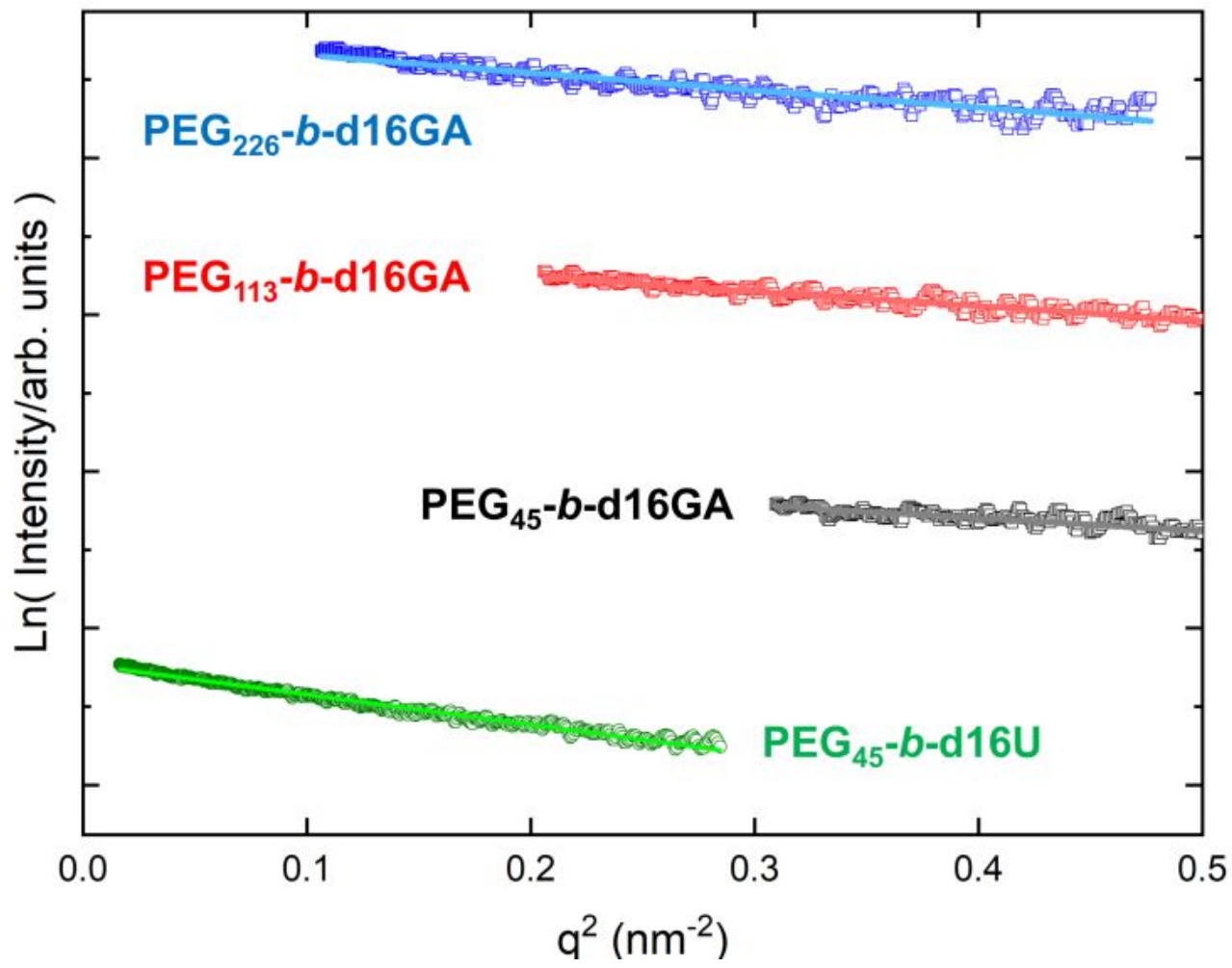

Figure S24. Guinier Plot of LDBCs in water at polymer concentration of 1.0 mg mL^{-1}.

Mass fractal model

The experimental SAXS data were fitted using the Mass Fractal model[4] implemented in SASView,[5] which describes the scattering intensity as:

$$I \sim P(q)S(q) \tag{S1}$$

where *P(q)* accounts for the form factor of the primary particles and *S(q)* represents the fractal structure factor, defined as:

$$S(q) = \frac{\Gamma(D_m - 1)\xi^{D_m - 1}}{\left[1 + (q\xi^2)\right]^{(D_m - 1)/2}} \frac{sin\left[(D_m - 1)\right]tan^{-1}(q\xi)}{q} \tag{S2}$$

The fit yielded a primary particle radius of 4.1 nm, a mass fractal dimension $D_m = 2.44$, and a cutoff length ξ = 10.3 nm, indicating a moderately branched fractal aggregate with finite size. The fractal dimension suggests a structure that is neither fully compact ($D_m \approx 3$) nor highly tenuous ($D_m \approx 1$), in agreement with the TEM images.

Scattering from flat objects

The scattering of flat object can be described as:[6,7]

$$P(q) = \left[\frac{2J_1(qR)}{qR}\right]^2 \cdot \left[\frac{\sin\left(\frac{qt}{2}\right)}{\frac{qt}{2}}\right]^2 \quad \text{(S3)}$$

Where J_1 is the First order Bessel function. The first term is associated to the lateral contribution, and the second to the thickness contribution. If the radius (*R*) of the disk is very large, the first term tends to 1, therefore, the scattering from an infinite plate of thickness *t* is:

$$P(q) = \left[\frac{\sin\left(\frac{qt}{2}\right)}{\frac{qt}{2}}\right]^2 \quad \text{(S4)}$$

The fit in Figure 2i was obtained by using a model including both contributions: A scattering from the plates with thickness t (Equation S4), allowing some polydispersity of this magnitude, added to a Porod contribution of the form: Aq^{-4}.

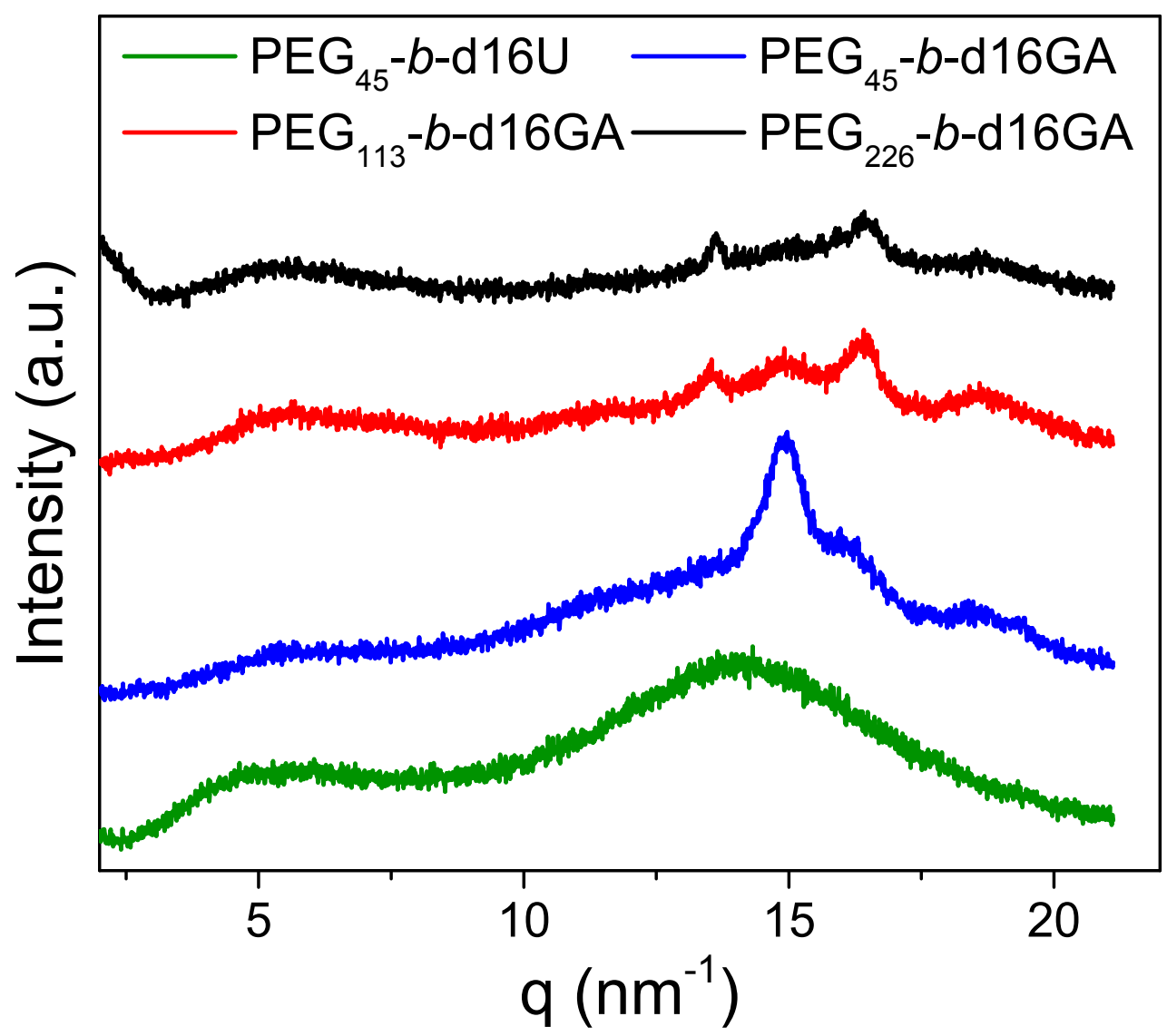

Figure S25. XRD patterns of dried **PEG$_{45}$-*b*-d16U** and **PEG$_m$-*b*-d16GA** self-assemblies.

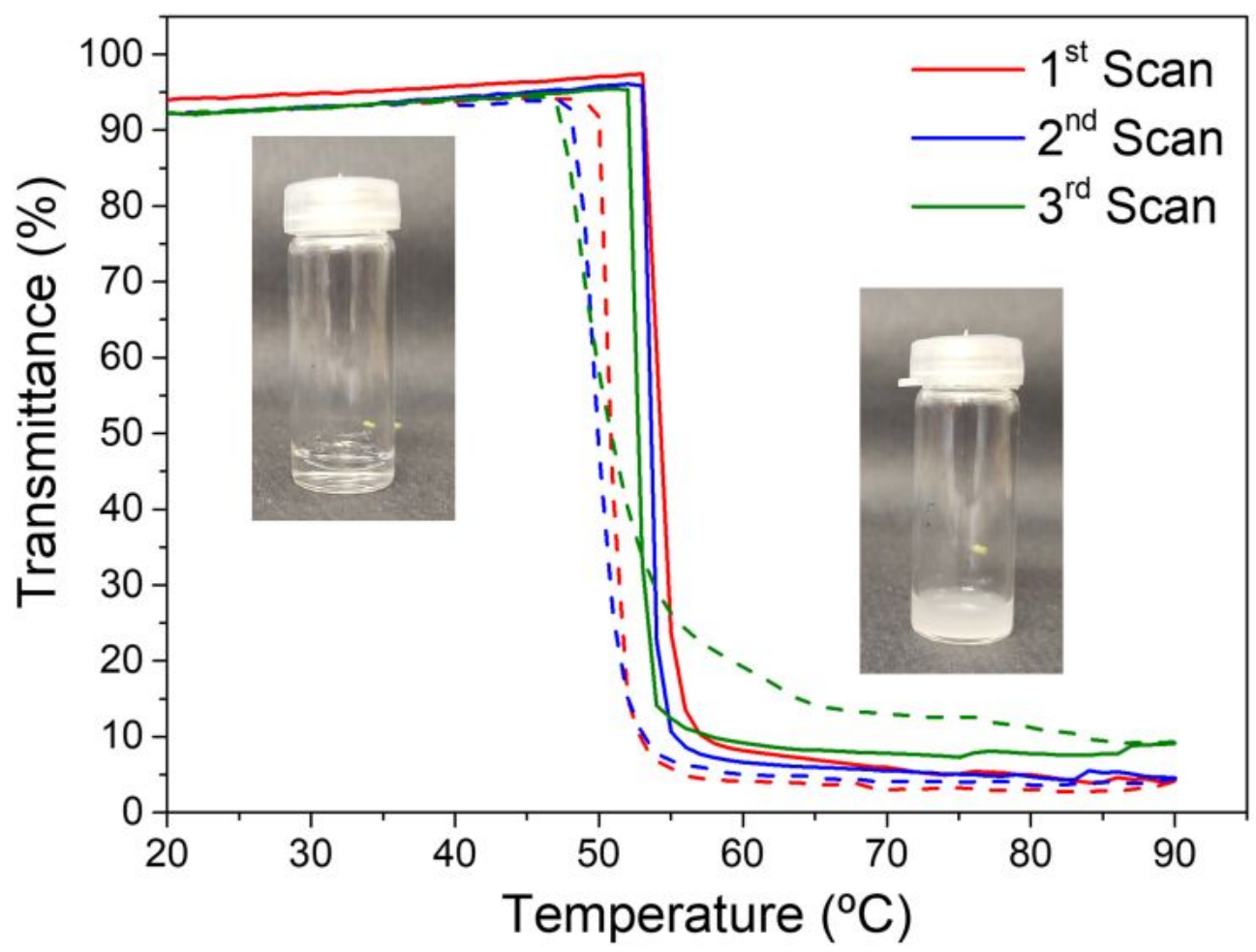

Figure S26. Temperature-dependent transmittance (650 nm) curves of **PEG$_{45}$-*b*-d16U** dispersions in water at a 1.0 mg mL^{-1} polymer concentration upon repeated heating (solid line) and cooling (dashed line).

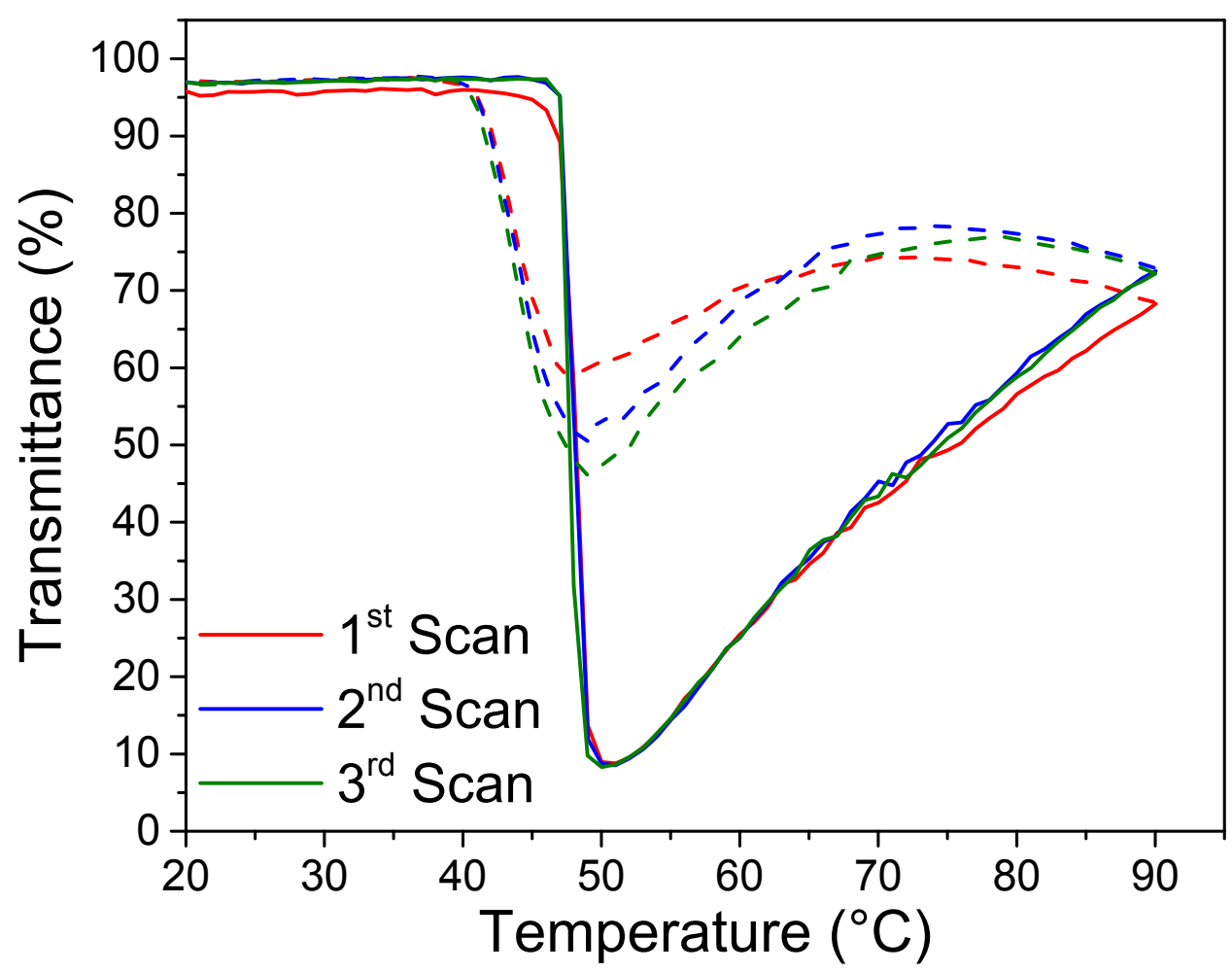

Figure S27. Temperature-dependent transmittance curves of **PEG$_{45}$-*b*-d16U** dispersion in PBS at a 1.0 mg mL^{-1} concentration upon repeated heating (solid line) and cooling (dashed line).

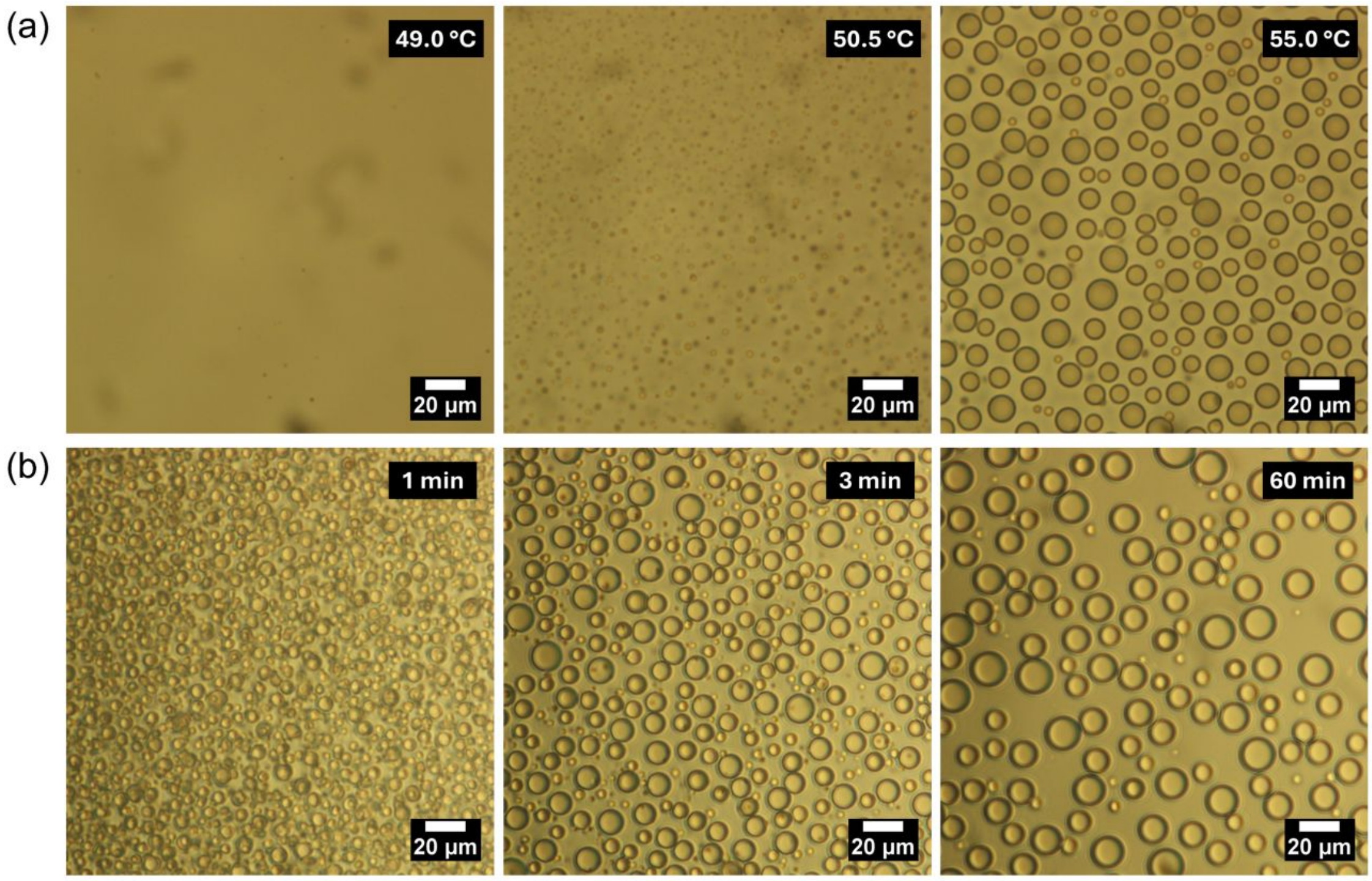

Figure S28. (a) Optical microscopy photographs of **PEG$_{45}$-*b*-d16U** at 10.0 mg mL^{-1} upon heating from 48 ˚C to 65 ˚C at 0.5 ˚C min^{-1} and (b) maintained at 60 ˚C for varying times keeping the temperature at 60 ˚C for different times.

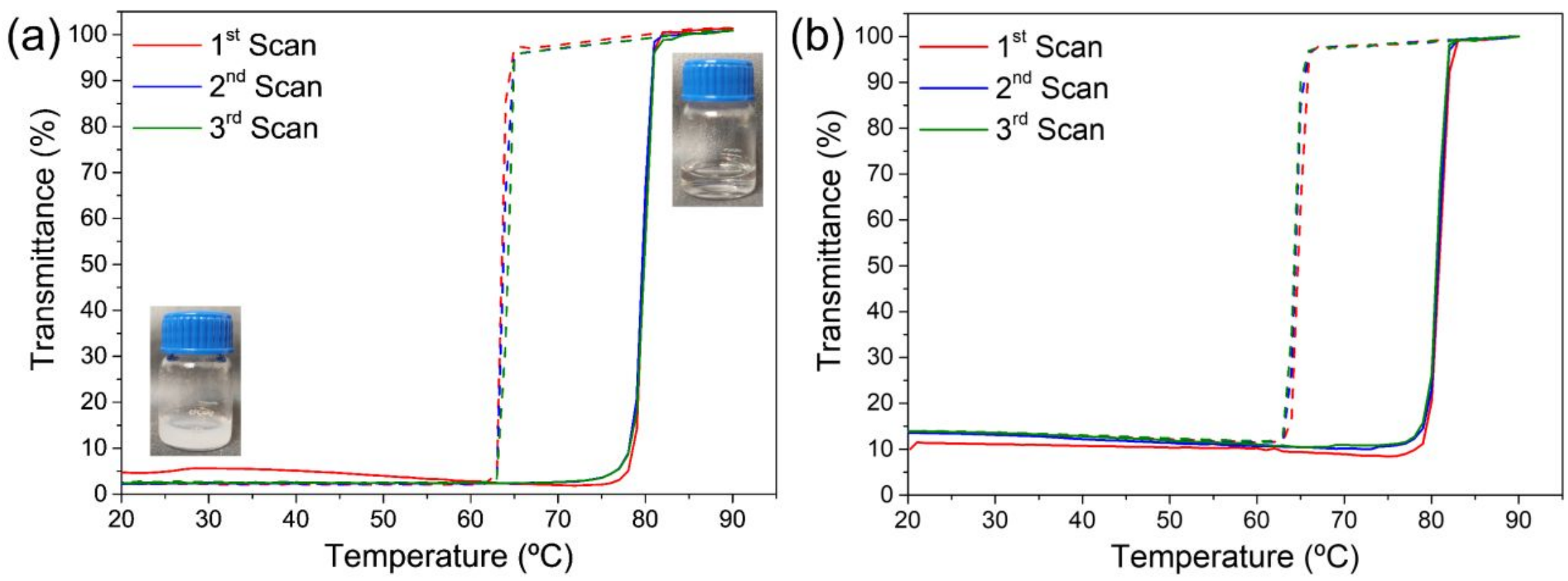

Figure S29. Temperature-dependent transmittance (650 nm) of **C$_6$-d16GA** dispersions (1.0 mg mL^{-1}). (a) Transmittance curves in water upon repeated heating (solid line) and cooling (dashed line). (b) Transmittance curves in PBS upon repeated heating (solid line) and cooling (dashed line).

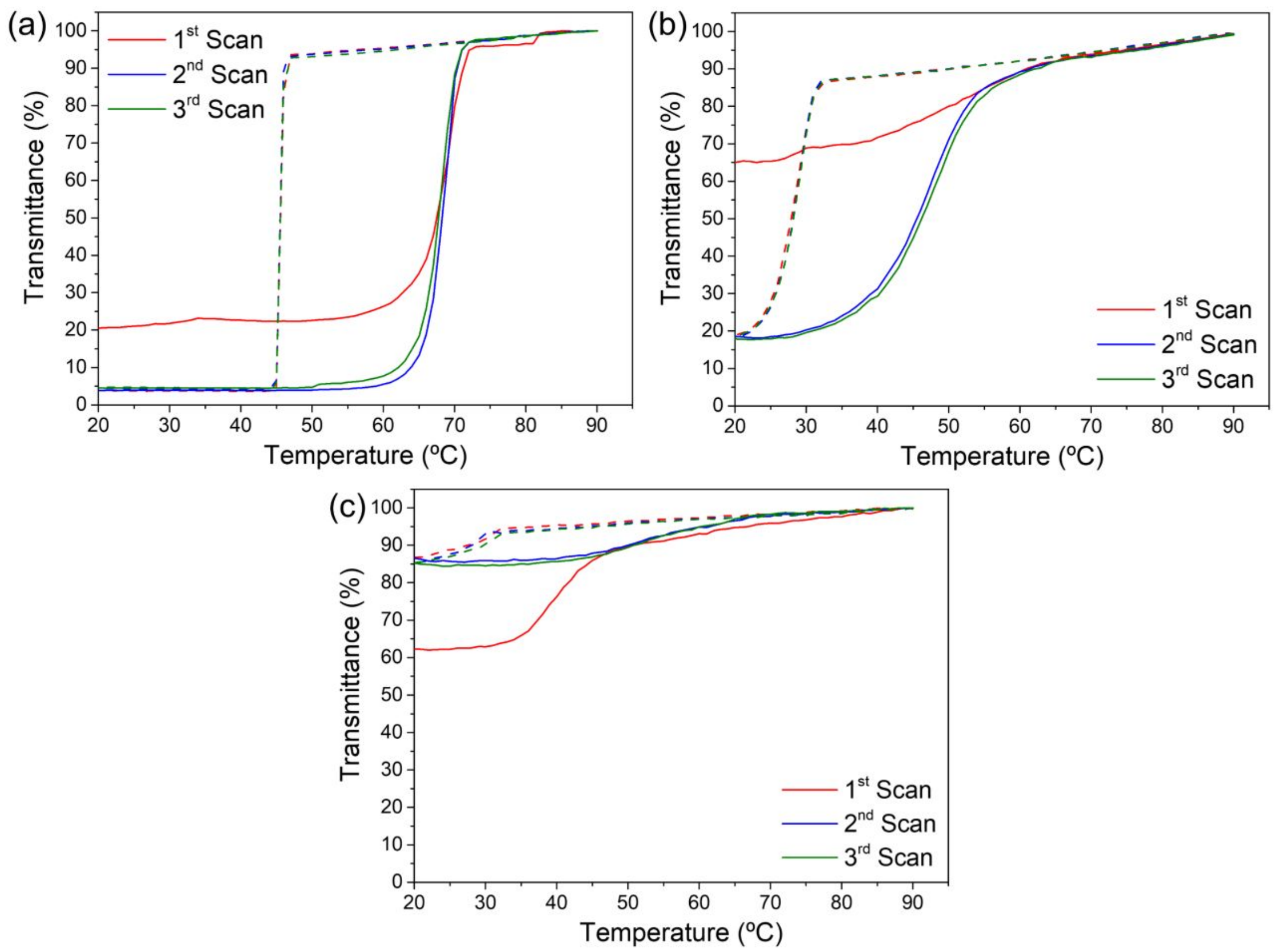

Figure S30. Temperature-dependent transmittance (650 nm) curves of (a) **PEG$_{45}$-*b*-d16GA**, (b) **PEG$_{113}$-*b*-d16GA** and (c) **PEG$_{226}$-*b*-d16GA** dispersions in water at a 1.0 mg mL^{-1} polymer concentration upon repeated heating (solid line) and cooling (dashed line).

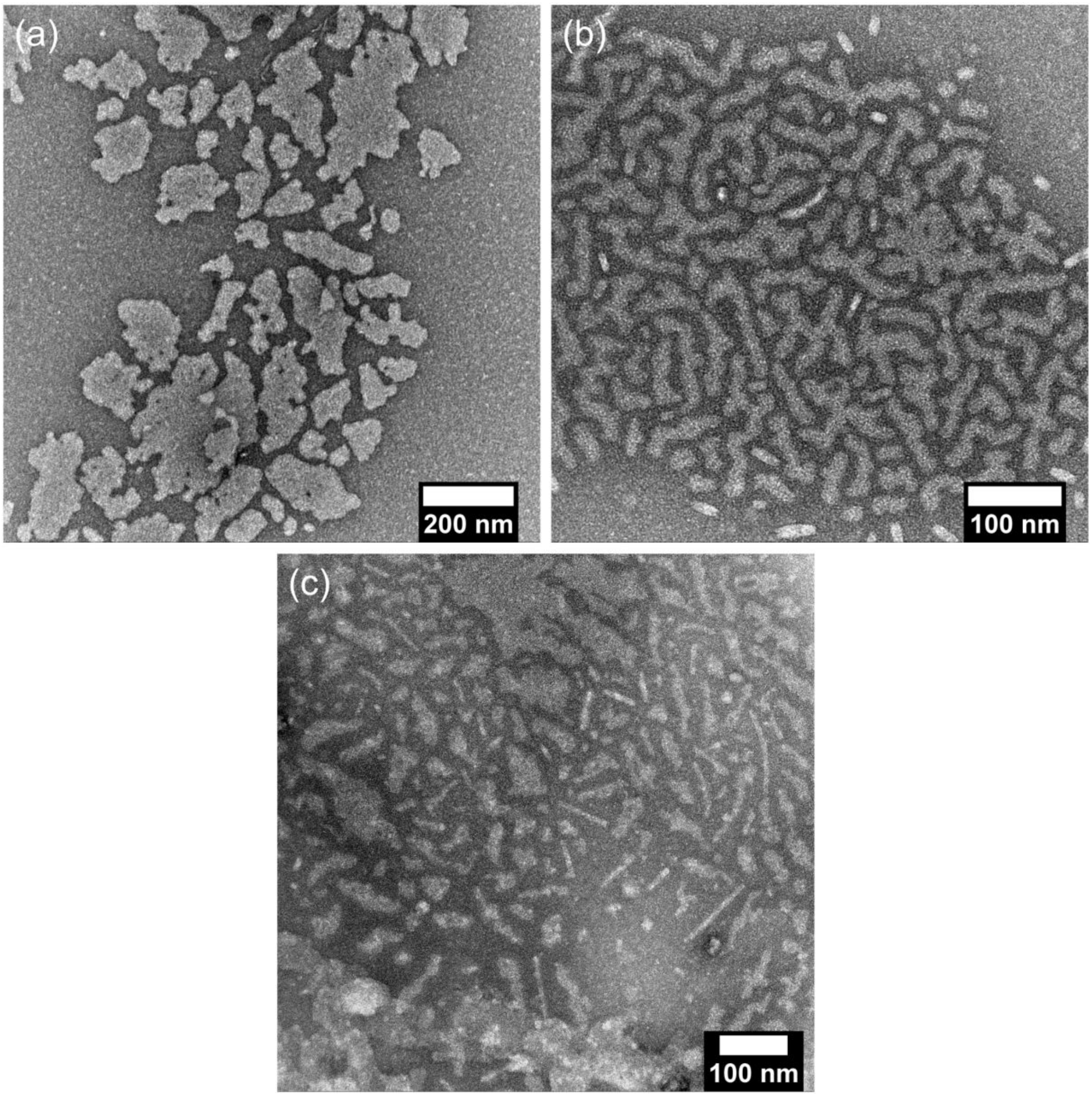

Figure S31. TEM images of (a) **PEG$_{45}$-*b*-d16GA**, (b) **PEG$_{113}$-*b*-d16GA** and (c) **PEG$_{226}$-*b*-d16GA** self-assemblies in water after first heating/cooling cycle at a 1.0 mg mL^{-1} polymer concentration.

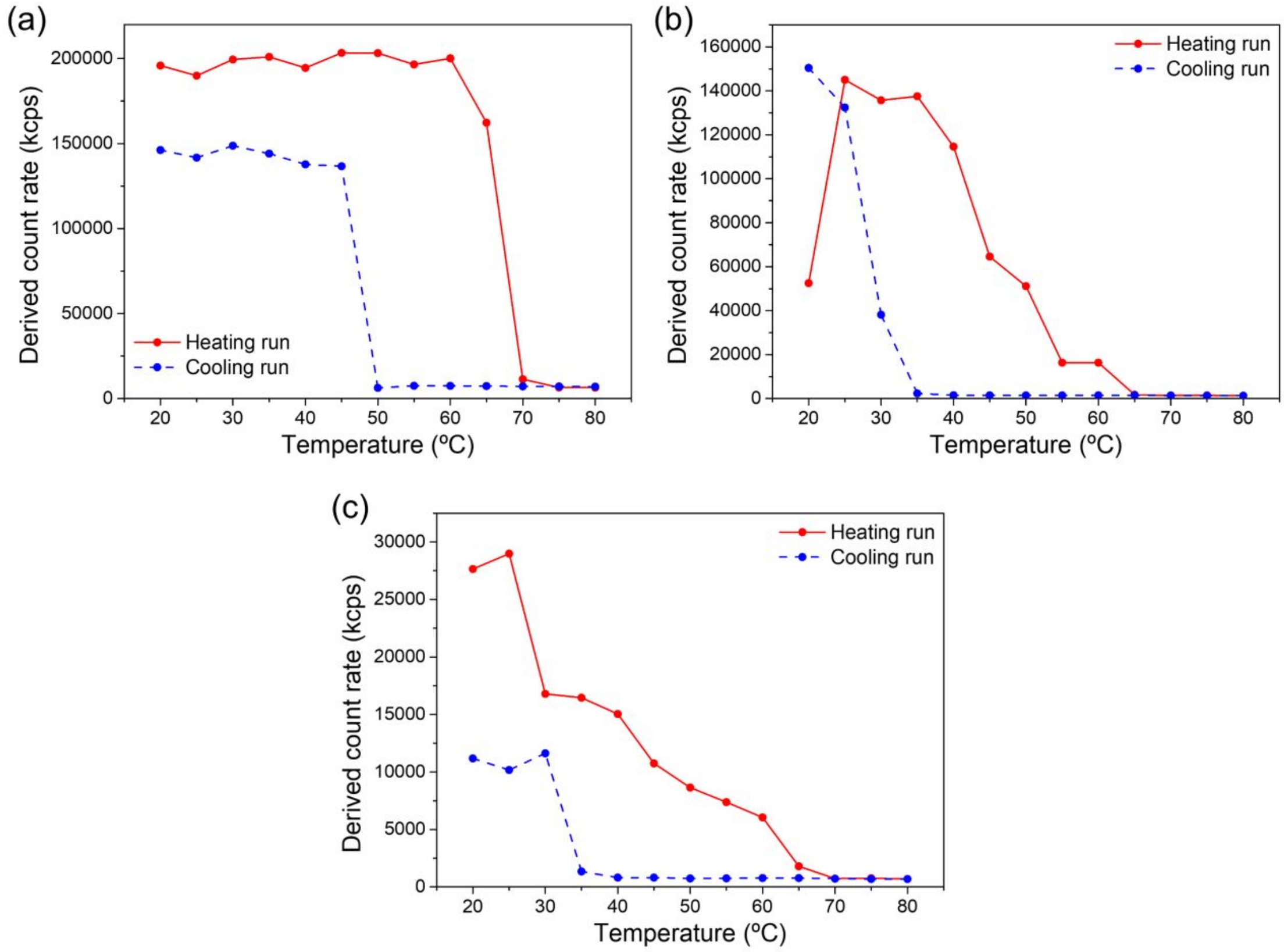

Figure S32. Temperature evolution of derived count rate (kcps) for (a) **PEG$_{45}$-*b*-d16GA**, (b) **PEG$_{113}$-*b*-d16GA** and (c) **PEG$_{226}$-*b*-d16GA** aqueous dispersions at a 1.0 mg mL^{-1} polymer concentration upon heating (solid line) and cooling (dashed line).

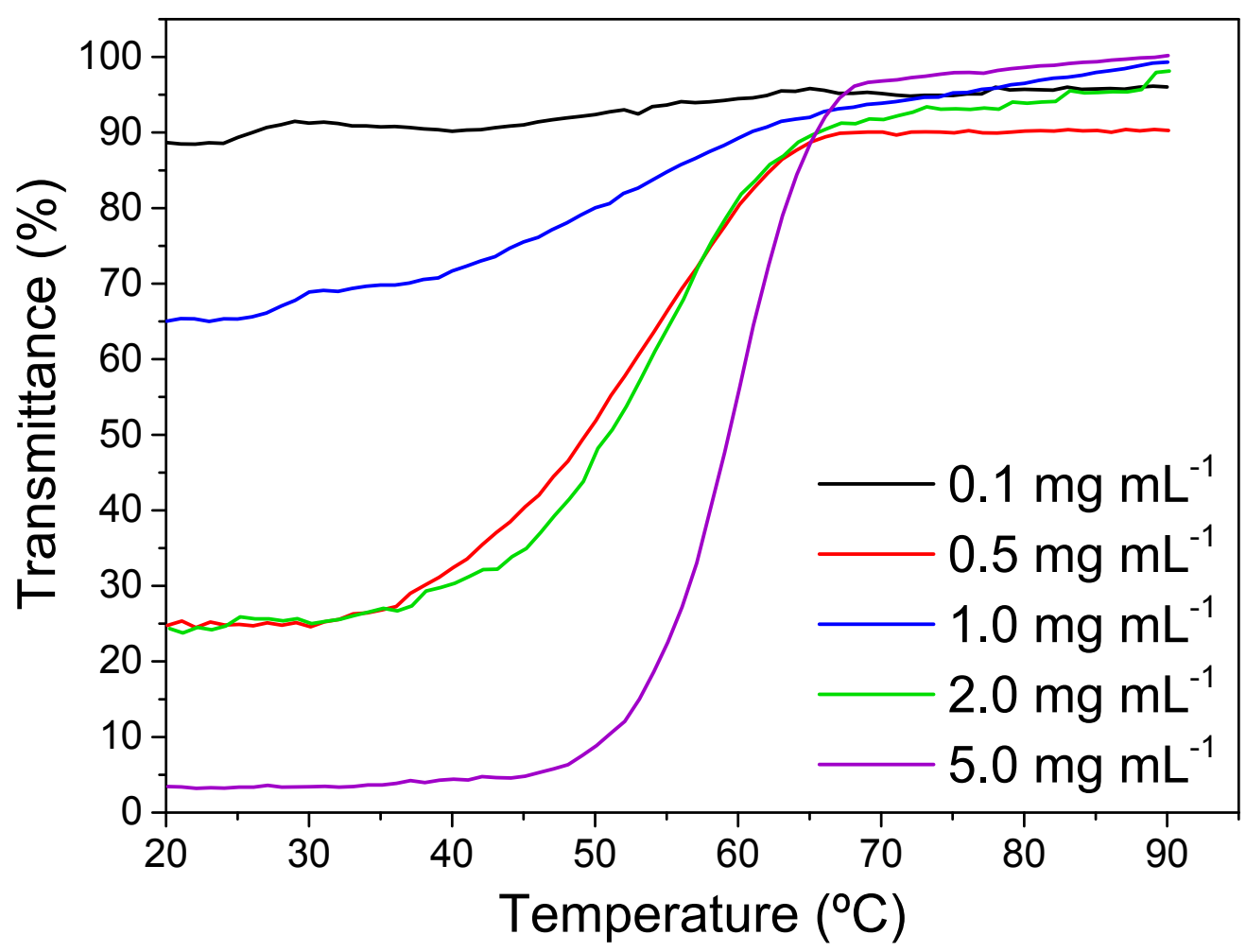

Figure S33. Temperature-dependent transmittance curves of **PEG$_{113}$-*b*-d16GA** dispersions in water at different polymer concentrations registered upon the first heating.

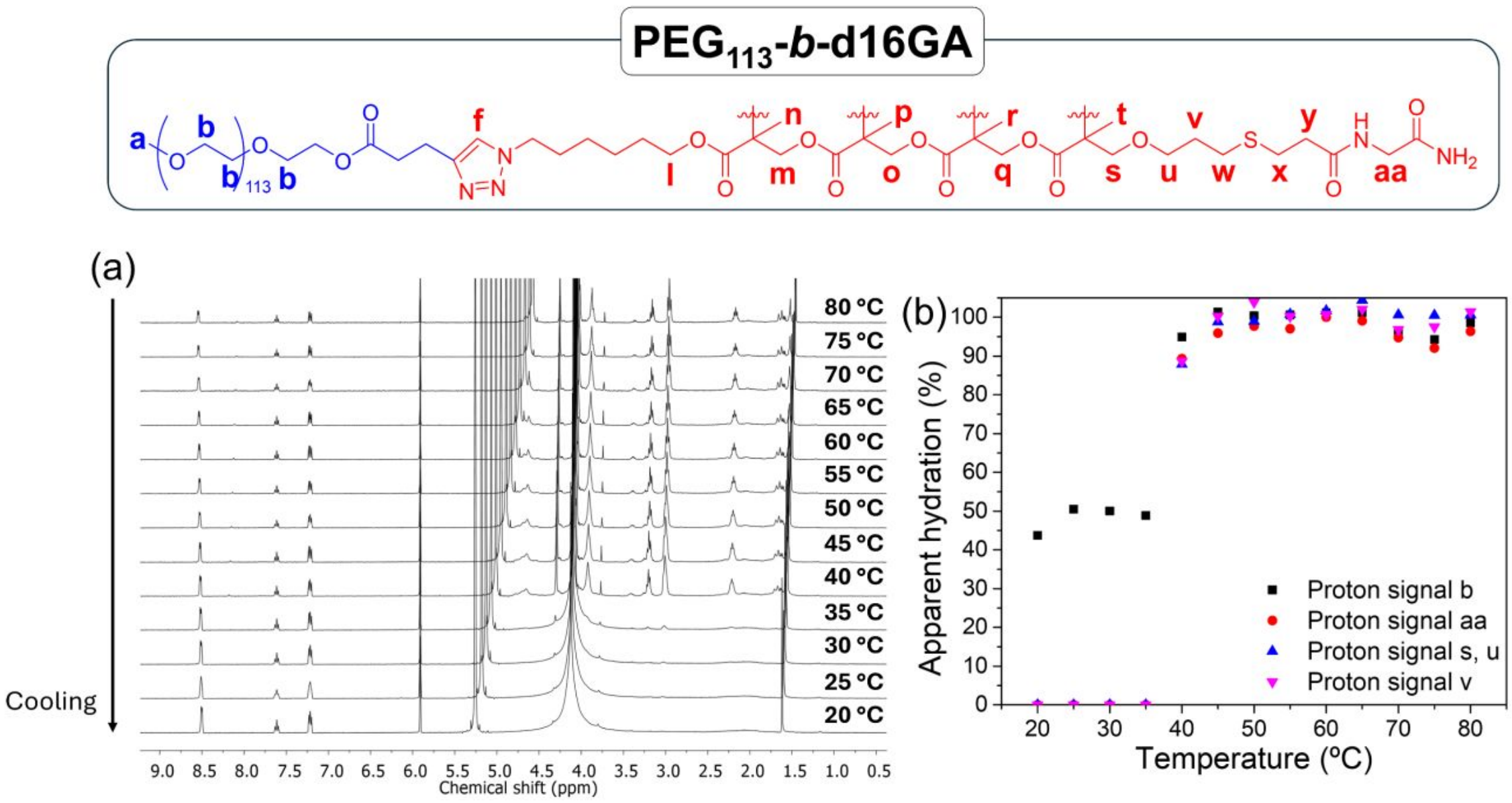

Figure S34. (a) ^{1}H NMR (400 MHz, D_2O) spectra of **PEG$_{113}$-*b*-d16GA** at 5.0 mg mL^{-1} upon cooling. (b) Temperature-dependent evolution of the NMR normalized intensities in D_2O relative to normalized intensities in DMSO-d_6 of **PEG$_{113}$-*b*-d16GA** upon cooling.

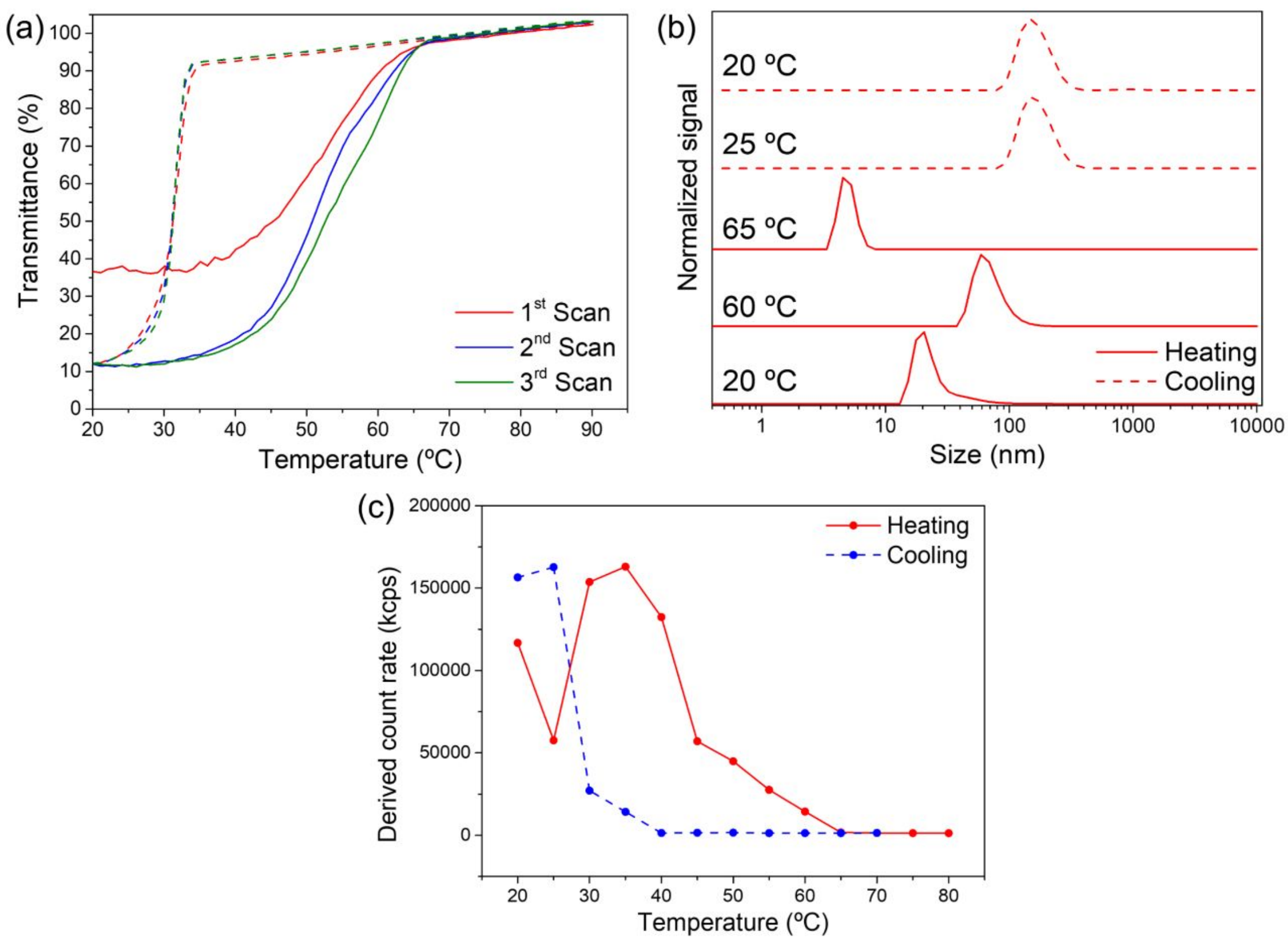

Figure S35. (a) Temperature-dependent transmittance curves of **PEG$_{113}$-*b*-d16GA** dispersion in PBS at a 1.0 mg mL^{-1} concentration upon repeated heating (solid line) and cooling (dashed line). (b) Temperature-dependent DLS number size distributions of **PEG$_{113}$-*b*-d16GA** upon heating (solid line) and cooling (dashed line). (c) Temperature evolution of derived count rate (kcps) for **PEG$_{113}$-*b*-d16GA** upon heating (solid line) and cooling (dashed line). Samples prepared in PBS at concentration of 1.0 mg mL^{-1}

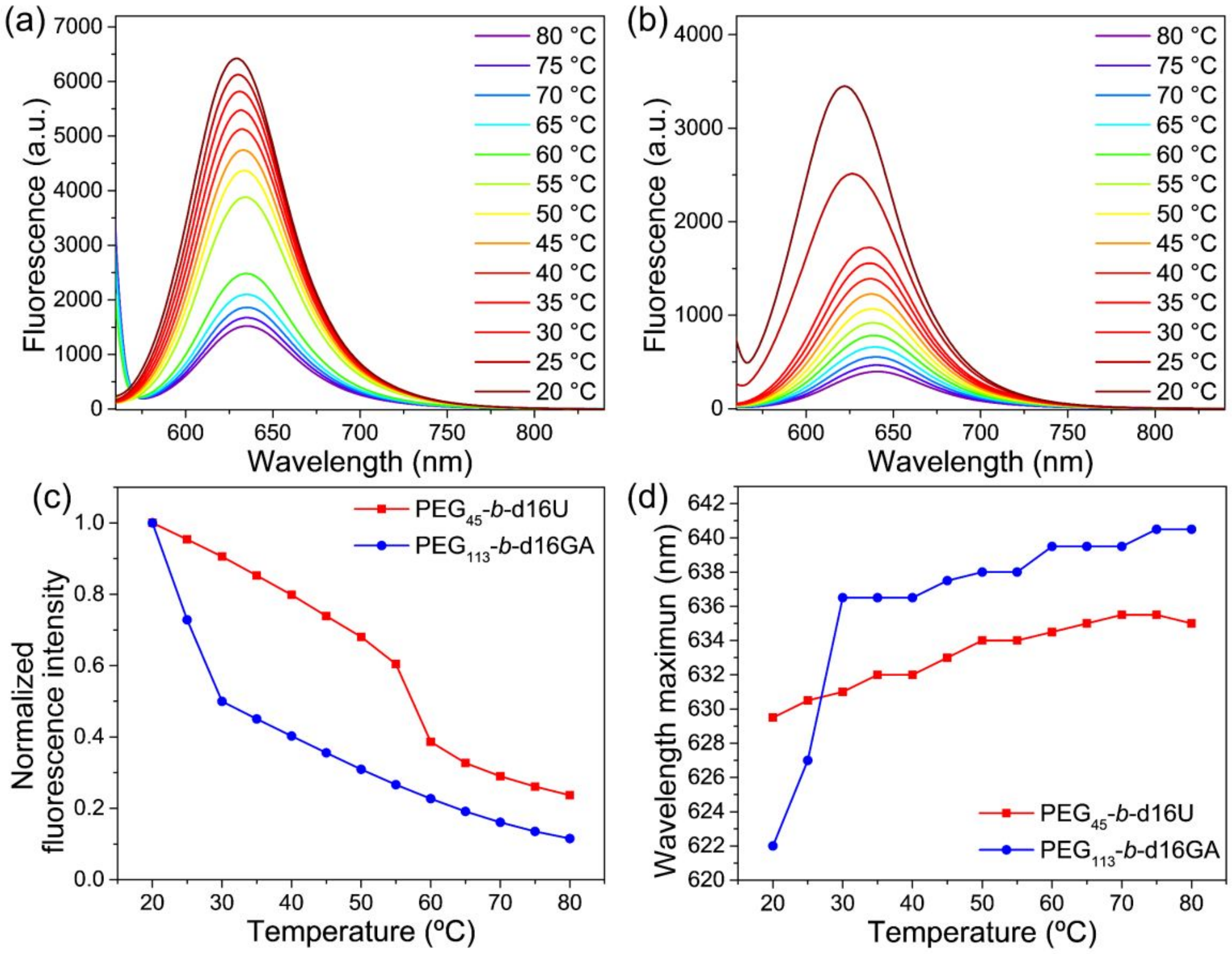

Figure S36. Variable temperature emission spectra at 1.0 mg mL^{-1} of (a) **PEG$_{45}$-*b*-d16U** and (b) **PEG$_{113}$-*b*-d16GA** dispersion loaded with 1.0×10^{-6} M of Nile Red (λexc. = 550 nm) upon cooling. (c) Temperature-dependent evolution of the normalized maximum fluorescence intensity of Nile Red upon cooling. (d) Temperature-dependent evolution of wavelength maximum of Nile Red upon cooling.

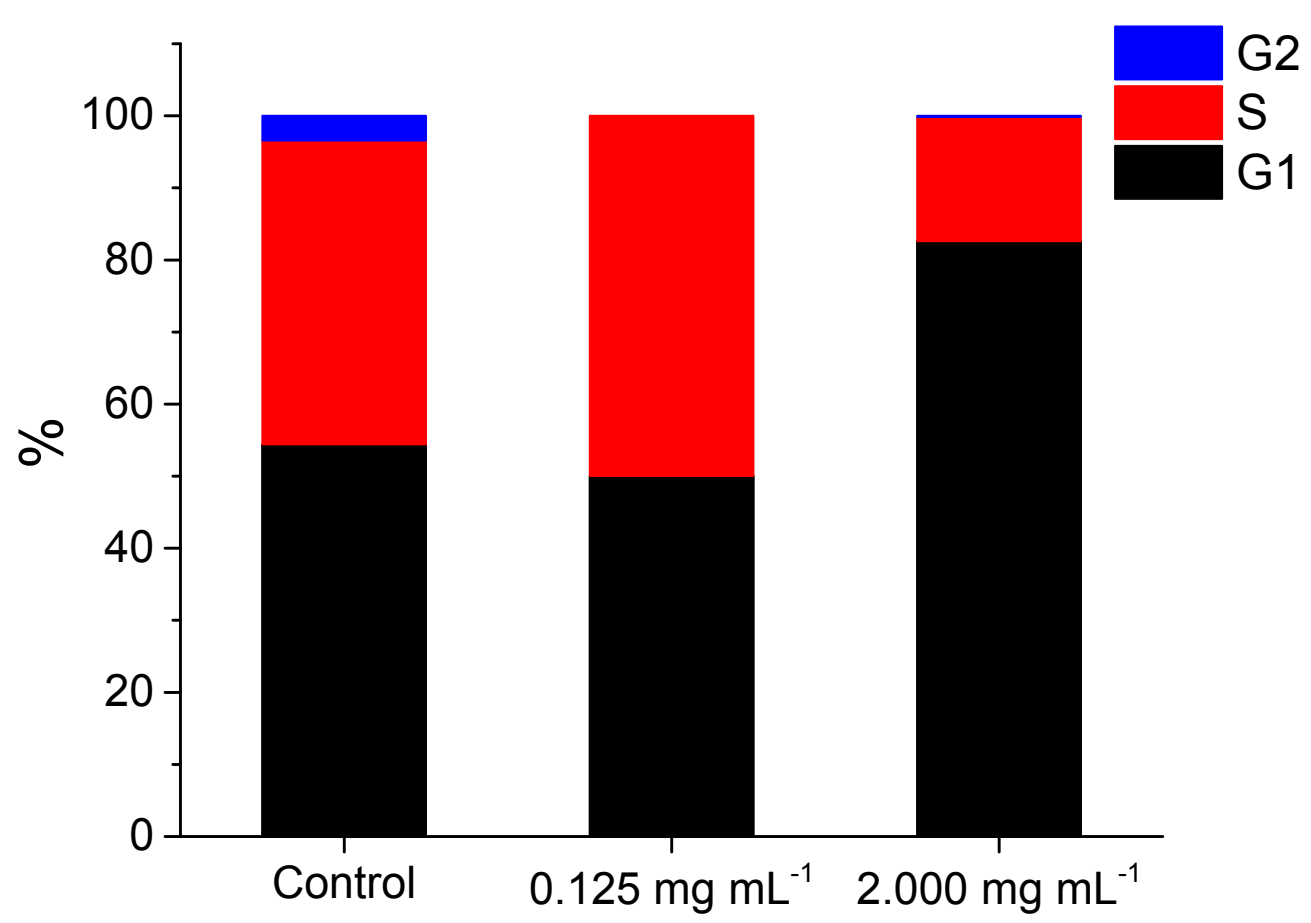

Figure S37. Distribution of cell cycle phases in J774A.1 macrophages exposed to **PEG_{113}-*b*-d16GA** self-assemblies at different polymer concentrations.

References

(1) Martin-Martin, J.; Abad, M.; Lopez De Pariza, X.; Ezquerra, T. A.; Nogales, A.; Sardon, H.; Sebastián, V.; Oriol, L.; Piñol, M. Degradable Ureido-Polycarbonate Block Copolymers with a Complex UCST Thermoresponse. *Macromol. Rapid Commun.* **2025**, 2500029. https://doi.org/10.1002/marc.202500029.

(2) del Barrio, J.; Oriol, L.; Alcalá, R.; Sánchez, C. Azobenzene-Containing Linear−Dendritic Diblock Copolymers by Click Chemistry: Synthesis, Characterization, Morphological Study, and Photoinduction of Optical Anisotropy. *Macromolecules* **2009**, *42* (15), 5752–5760. https://doi.org/10.1021/ma9003133.

(3) Martín-Martín, J.; Bescós-Ramo, S.; Terrel, A.; Arruebo, M.; Piñol, M.; Yus, C.; Oriol, L. Antibiotic-like Properties of Cationic Linear-Dendritic Macromolecules Having Guanidinium Peripheral Groups. *Materials Today Chemistry* **2026**, *51*, 103342. https://doi.org/10.1016/j.mtchem.2025.103342.

(4) Mildner, D. F. R.; Hall, P. L. Small-Angle Scattering from Porous Solids with Fractal Geometry. *J. Phys. D: Appl. Phys.* **1986**, *19* (8), 1535–1545. https://doi.org/10.1088/0022-3727/19/8/021.

(5) Doucet, M.; Butler, P.; Kienzle, P.; Cho, J. SasView Version 6.1.0, 2023.

(6) Feigin, L. A.; Svergun, D. I. *Structure Analysis by Small Angle X-Ray and Neutron Scattering*; Plenum Press: New York, 1987.

(7) Glatter, O.; Kratky, O. *Small Angle X-Ray Scattering*; Academic Press: London, 1982.
